# A systematic review and meta-analysis of psychological and behavioural responses in human-agent vs. human-human interactions

**DOI:** 10.1038/s44271-026-00466-z

**Published:** 2026-05-05

**Authors:** Jianan Zhou, Fleur Corbett, Joori Byun, Talya Porat, Nejra van Zalk

**Affiliations:** 1 Dyson School of Design Engineering, Imperial College London, London, UK; 2 Independent Researcher, London, UK

**Keywords:** Human behaviour, Computational science

## Abstract

Interactive intelligent agents are being integrated across society. Despite achieving human-like capabilities, humans’ responses to these agents remain poorly understood, with research fragmented across disciplines. We conducted a systematic synthesis comparing a range of psychological and behavioural responses in matched human-agent vs. human-human dyadic interactions. A total of 162 eligible studies (146 contributed to the meta-analysis; 468 effect sizes) were included in the systematic review and meta-analysis, which integrated frequentist and Bayesian approaches. Our results indicate that individuals exhibited less prosocial behaviour and moral engagement when interacting with agents vs. humans. They attributed less agency and responsibility to agents, perceiving them as less competent, likeable, and socially present. In contrast, individuals’ social alignment (i.e., alignment or adaptation of internal states and behaviours with partners), trust in partners, personal agency, task performance, and interaction experiences were generally comparable when interacting with agents vs. humans. We observed high effect-size heterogeneity for many subjective responses (i.e., social perceptions of partners, subjective trust, and interaction experiences), suggesting context-dependency of partner effects. By examining the characteristics of studies, participants, partners, interaction scenarios, and response measures, we also identified several moderators shaping partner effects. Overall, functional behaviours and interaction experiences with agents can resemble those with humans, whereas fundamental social attributions and prosocial/moral concerns lag in human-agent interactions. Agents are thus afforded instrumental value on par with humans but lack comparable intrinsic value, providing implications for the development of interactive intelligent agents.

## Abstract (web summary)

This systematic review and meta-analysis found that functional behaviours and interaction experiences with intelligent agents can resemble those with humans, whereas social attributions and prosocial/moral concerns lag in human-agent interactions.

## Introduction

Interactive intelligent agents, such as chatbots, virtual humans, and robots, are increasingly embedded across professional and personal domains, including healthcare, education, business, and leisure^1^. Recent breakthroughs in artificial intelligence (AI) have advanced these systems towards, and in some cases beyond, human-level performance on specific tasks^2–5^. Leading technology labs like Google’s DeepMind suggest no inherent technical limit to these systems achieving human-level capabilities across various domains^6^. Evolving from narrow applications to broader competence, these systems move beyond reactive “tools” or “assistants” to proactive “agents”: autonomous computational systems capable of goal-directed behaviour, interaction with the environment, and task execution with minimal human involvement^7–10^. Human interaction with these agents fundamentally differs from traditional human-computer interaction^11^. Agents are assuming roles primarily reserved for humans, serving as companions, collaborators, advisors, and even opponents^12–15^. Scenarios such as cooperation, competition, and strategic encounter, originally confined to human-human interaction, now emerge in human-agent interaction^16–19^. These developments are blurring long-standing boundaries between humans and machines.

As people engage with intelligent agents in socially complex settings requiring psychological attunement^20–22^, a key question emerges: can agents’ human-like performance evoke the same psychological and/or behavioural responses—the way individuals feel, think and act within an interaction^23^—as those with human partners? This knowledge gap calls for rigorous investigation. For example, individuals may or may not maintain comparable *task performance* when collaborating with agents as they do with humans. Their sense of *personal agency* may vary during agent interaction. Core *social responses*, such as alignment, trust, and morality, may be inherently tied to human presence or may be elicited by agents displaying human-like behaviour. Insights into the transferability of human psychology and behaviour from human-human interaction to human-agent interaction are critical to inform responsible agent design and regulation^24–27^. It is thus important to compare a range of psychological and behavioural responses in human-agent vs. human-human interactions, where the task performance of agent and human partners is matched, and to further explore the conditions under which similarities and differences emerge.

Empirical evidence on human responses in human-agent vs. human-human interactions is fractured across disciplinary silos and methodological traditions. The rise of interactive intelligent agents has attracted scholars beyond computer science and robotics, including psychology, communication, business, and marketing, spurring a proliferation of related studies^28^. Existing research exhibits inconsistent constructs, divergent methodologies, and isolated investigations. Specifically, similar underlying constructs have been operationalised using different indicators and measures. Taking trust towards partners as an example, it has been assessed using multiple indicators, including perceived trustworthiness, trust intention, self-reported trust, and behavioural measures^29–32^. Even when nominally assessing the same indicator, studies use various instruments that capture different aspects and levels of abstraction^33^. Research paradigms also vary considerably. Many studies examine participant responses through direct interaction with agent or human partners or through vignette-based designs in which participants imagine interacting with partners and report hypothetical responses^34–36^. Other studies rely on passive evaluations of partners or their generated outputs within an “interaction” framing^37,38^. Varying experimental control, such as mismatched partner behaviour or task performance, can further confound the interpretation of response comparability^39–41^. Moreover, many investigations remain isolated as one-off demonstrations, with limited follow-up, replication, or systematic extension to establish cumulative evidence^42,43^. These practices have contributed to largely mixed empirical findings^44,45^. For instance, some studies report no significant difference in subjective trust between agent and human partners^46^, whereas others reveal significant differences^47^ or qualitatively distinct trust responses^48^. It remains unclear whether such inconsistencies stem from research designs, agent types, interaction tasks, or participant demographics.

The mixed empirical evidence is mirrored by a diversity of theoretical perspectives. For example, the Computers Are Social Actors (CASA) paradigm and its predecessor, the Media Equation, propose that individuals respond to intelligent agents with minimal human-like cues as they would to humans, suggesting that social responses in human-agent interaction mirror those in human-human interaction^49,50^. This proposition of equivalence, however, is challenged by other frameworks. The Threshold Model of Social Influence draws a key distinction: when behaviour appears equally realistic, individuals show stronger deliberate social responses when they believe they are with humans rather than agents in virtual settings—though automatic, low-level reactions remain unchanged across both^51^. Theories of anthropomorphism shift focus to the attribution of human-like characteristics to agents’ real or imagined behaviour^52,53^. It has been suggested that anthropomorphising behaviour (i.e., the observable ways in which individuals respond to agents as they would to humans) should be studied in human-agent interaction without presuming response equivalence to human-human interaction^54^. Theories focusing on social perceptions of agents provide additional insights. For example, the Modality-Agency-Interactivity-Navigability (MAIN) model proposes that perceived agency (human vs. algorithm) activates different cognitive heuristics influencing perceived credibility^55^. The Social Presence Theory examines the degree to which agents are perceived as real social actors^56^, with a meta-analysis showing how social cues in agents increase perceived social presence^57^. The Uncanny Valley Theory introduces a critical caveat regarding human-like design, warning that near-human realism with subtle imperfections evokes eeriness and discomfort^58^. This abundance of existing theoretical frameworks underscores the need for a cross-disciplinary, systematic understanding of human responses in human-agent vs. human-human interactions, and for identifying factors influencing their similarities or differences.

Five previous reviews have addressed related topics. One narrative review argued that humans engage with agents in ways resembling interaction with other humans, including relationship building, suggesting that similarities outweigh differences and that theories of human-human interaction apply to human-agent interaction^25^. By contrast, another narrative review cautioned against equating these two interactions, arguing that current agents lack key social affordances underpinning theories of human-human interaction and urging the development of models specific to human-agent interaction^27^. An integrative review comparing trust across both interactions proposed similar developmental processes but differences in expression and calibration, and suggested narrowing gaps between perceptions of humans and agents to improve user trust^59^. Nevertheless, these reviews have two primary limitations. They prioritised argument-driven theoretical integration over evidence-driven systematic synthesis. Also, they lacked focus on literature directly comparing responses between these two interactions, which, even when revealing response similarities, only metaphorically support the Media Equation^60^.

One meta-analysis systematically compared responses in human-agent and human-human interactions by specific partner types (virtual agents vs. avatars) and found that avatars exerted stronger social influence than agents^45^. While insightful, this review collapsed responses (e.g., subjective perceptions, affect, task performance, physiological measures) into uniform so-called measures of social influence and pooled them in one meta-analysis. Although most psychological and behavioural responses in human-agent and human-human interactions fall under the umbrella of social responses^61^, treating them as a monolithic entity obscures conceptual distinctions. Another meta-analysis similarly synthesised diverse responses under the construct of persuasion and found that AI agents did not significantly differ from humans in overall persuasion outcomes^44^. Its operationalisation of persuasion encompassed a broader range of responses than persuasion in the conventional sense. For example, some included studies assessed evaluative or experiential responses (e.g., trustworthiness, credibility, pleasantness, affinity, and communication competence)^62–65^. In addition, many responses were measured in paradigms where participants evaluated AI- or human-generated outputs without engaging in interaction with a communicative partner. Accordingly, existing reviews have yet to offer a holistic picture of how various types of human responses compare between human-agent and human-human interactions.

## The current review

To address prior limitations, we conducted a systematic review and meta-analysis of individuals’ psychological and behavioural responses in dyadic interactions with agent vs. human partners. The partners were functionally equivalent (i.e., performance-matched) to ensure comparability. We quantified the effects of partner type (agent vs. human; hereafter, *partner effects*) on specific responses and explored moderators of these partner effects. Specifically, we aimed to answer four research questions:

- RQ1: Which psychological and behavioural responses have been investigated in studies comparing human-agent and human-human interactions?
- RQ2: Which specific response types differ between human-agent and human-human interactions?
- RQ3: Which specific response types are similar between human-agent and human-human interactions?
- RQ4: To what extent do study, participant, partner, interaction, and response characteristics moderate partner effects on different response types?

As discussed above, multiple theoretical perspectives seek to explain human responses to intelligent agents. These perspectives offer overlapping yet partially conflicting predictions regarding the extent to which humans engage with agents in ways resembling interaction with other humans. Consequently, the current review employed a theory-integrative approach without relying on a single deductive theoretical lens. Rather than presuming uniform equivalence or systematic divergence, we treated response comparability as an empirical question to be investigated across psychological and behavioural constructs. In addition to quantifying the extent of similarity and difference across distinct response types, we also aimed to identify empirical boundary conditions under which theoretical predictions of equivalence or divergence are supported.

Aligning with a human-centred perspective, we treated “agent” as a functional metaphor rather than a strict technical term^7^. A computational system was considered an intelligent *agent* if it assumed a human-equivalent role while matching the task performance of its human counterpart, irrespective of technical implementation. Agent architectures may include traditional machine learning, rule-based algorithms, Wizard-of-Oz setups, or more recent generative AI. In addition, the term “interaction” is central to human-computer and human-agent interaction research, yet remains overloaded and ambiguous^66^. It has been understood in various ways: as an experiential stream of subjective expectations, experiences, and memories^67^; as a system’s disposition for interaction from a design perspective^68,69^; or as a process involving mutual exchanges^70^. Notably, we define *interaction* as the engagement between participants and partners for some purpose within certain contexts, involving reciprocal information exchange or unidirectional transmission with at least one party taking an active role. This requires participatory engagement, where participants act as interactants rather than observers in real-time or hypothetical (i.e., vignette-based imaginative) scenarios. Studies in which participants only evaluated AI or human partners, or their generated content, were not considered to involve interaction. Given critiques of understanding interaction as mutual exchanges in human-computer interaction^66^, we also included unidirectional interactions—for example, participants speak while partners listen or vice versa—provided partners are not presented as passive prerecorded stimuli but possessing interactability (i.e., the disposition and readiness to engage)^71^.

**Fig. 1:**
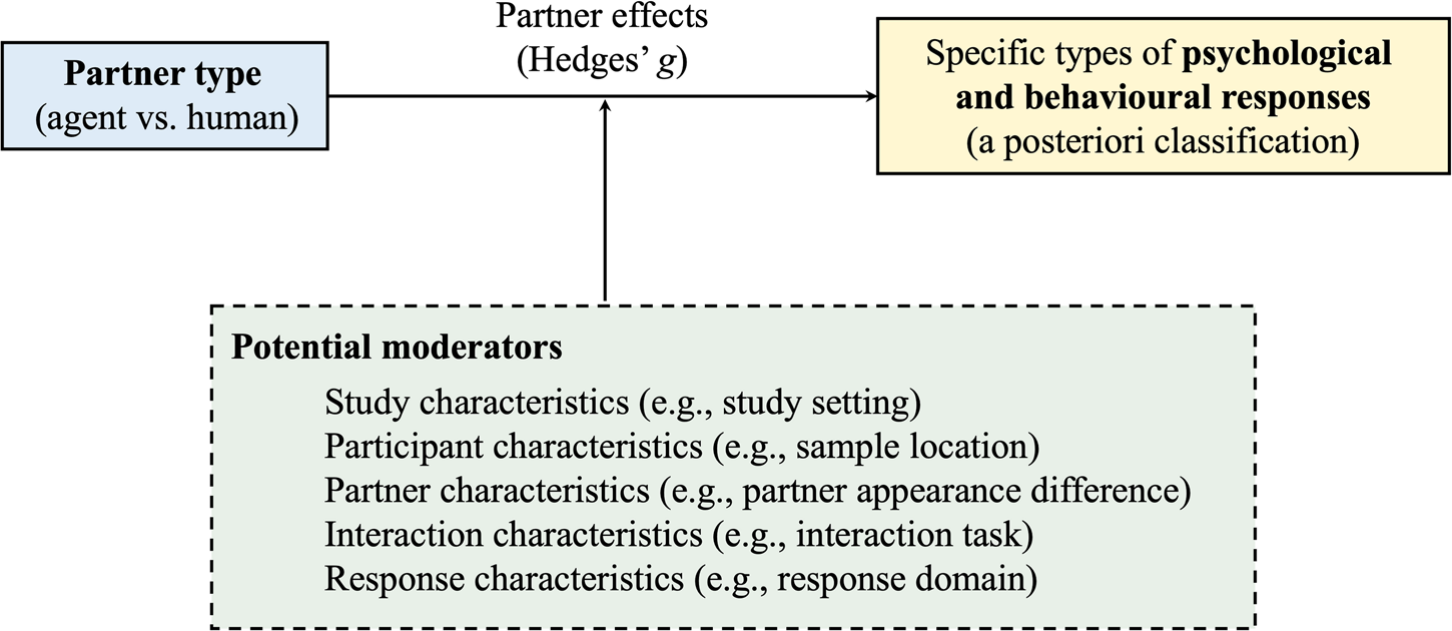
Review conceptual framework. Partner type (agent vs. human) was examined as the independent variable. Psychological and behavioural responses extracted from individual studies were classified a posteriori into distinct response types and meta-analysed separately. Partner effects were quantified as standardised mean differences (Hedges’ *g*) between human-agent and human-human interactions for different response types. Five categories of potential moderators were examined to explore sources of heterogeneity in partner effects.

Furthermore, mixed empirical evidence on human responses in human-agent vs. human-human interactions implies the existence of moderating effects. We chose potential moderators based on prior related meta-analyses examining heterogeneity in human responses. Meta-analytic work comparing virtual agents and avatars in social influence examined moderators such as agency operationalisation (human- or algorithm-controlled), task nature (cooperative, competitive, or neutral), and response measure (subjective or objective)^45^. Another work comparing AI and human communicators in persuasion examined moderators related to participant sociodemographics, study setting (lab, online, or field), communication direction (unidirectional or bidirectional), and response domain (behaviour, perception, attitude, or intention)^44^. Four meta-analyses on the effects of agent anthropomorphism and social cues on human responses examined characteristics of studies (e.g., study setting, publication year), samples (e.g., age, gender, location), agents (e.g., appearance, voice, embodiment, physical or virtual form), and contexts (e.g., task type, structured or unstructured interaction)^57,72–74^. Drawing on this body of work and the Media Are Social Actors (MASA) paradigm, which posits that agent features, individual differences, and contextual factors shape responses to agents^75^, we explored moderators spanning study, participant, partner, interaction, and response characteristics. The conceptual framework of the current review is visualised in Fig. 1.

## Methods

### Literature search and eligibility criteria

This systematic review and meta-analysis followed the Preferred Reporting Items for Systematic Reviews and Meta-Analyses (PRISMA) 2020 statement. It was not preregistered and had no formal protocol. The PRISMA checklist is available in Supplementary Table 1. We conducted systematic searches in February 2024 across four literature databases: Scopus (which covers IEEE Xplore^76^), Web of Science, ACM Digital Library, and PsycInfo. We searched titles, abstracts, and keywords using search strings comprising two components. The first component identified various types of interactive intelligent agents, while the second combined general and specific terms to capture a wide range of topics in human-human interaction research, including common interpersonal and social phenomena that have been studied in human-agent interaction. The searches focused on empirical research articles published from 2000 and employing quantitative or mixed methods (extracting only quantitative data) and were restricted to peer-reviewed journal or conference papers written in English. The full search strings can be found in Supplementary Table 2. Grey literature was not searched due to challenges in locating studies and the lack of a reliable method for assessing quality.

We defined the eligibility criteria via the PECO (Population, Exposure, Comparison, and Outcome) framework. Eligible studies were on healthy adult participants (P) that investigated both dyadic human-agent (E) and human-human (C) interactions and made direct, parallel comparisons of human responses (O) in these two conditions. Interactive intelligent agents could be either physical or virtual, embodied or disembodied. Participants actively engaged in research tasks that involved (perceived) real-time or hypothetical interactions with partners, whom participants believed to be either an agent or human. Additionally, participants received comparable treatment across human-agent and human-human interaction conditions; that is, the interaction task, dynamics, and partner role and performance were consistent across both conditions, except for the partner’s voice and appearance. When voice and appearance were matched between agent and human partners, the only difference lay in the perceived identity of the interaction partner, isolating the identity effects on human responses. However, as voice and appearance were often used to enhance the believability of the manipulated partner identity, variations in these two aspects were permitted. Lastly, human responses were examined in terms of participants’ individual-level psychological and behavioural responses during or after the interactions.

An example study^77^ is presented to illustrate the type of studies included in this review. In this study, participants delivered an impromptu speech and received feedback from either a robot or a human evaluator. To ensure functional equivalence, evaluator behaviour was matched across conditions: both robot and human evaluators followed an identical script consisting of praise and constructive feedback. Moreover, the human evaluator practiced the feedback delivery to match the robot’s pacing and animacy, and the physical height of both evaluators was matched. Following the interaction, participants reported their perceptions of the evaluator and the feedback received. Such a parallel experimental design enables the examination of partner effects by manipulating partner type while ensuring comparable participant treatment.

The detailed inclusion and exclusion criteria are provided in Supplementary Table 3. The study selection involved two stages: title-abstract screening and full-text screening. At each stage, the first author screened all the candidate articles, and two co-authors each independently screened a random 15% subset^78^. Any discrepancies were resolved through discussion.

### Data extraction and coding

The data extraction and coding protocol (including study details and essential statistics for effect size calculation; Supplementary Table 4) was established a priori, except that response classification and the specific levels of two moderators (i.e., interaction task and response dimension) were determined post hoc. The first author completed data extraction and coding for all eligible studies, and two co-authors each checked a random 15% subset. Any discrepancies were resolved through discussion.

Human responses examined across studies were diverse. Prior meta-analytic work has synthesised these responses under umbrella constructs (i.e., social influence^45^ and persuasion^44^), or grouped them into broad categories such as attitudes, perceptions, affect, and behaviour^73,74^. While informative, such categorisations may mask meaningful differences between conceptually distinct response types. Therefore, we performed an a posteriori classification and meta-analysed each response type separately. We initially applied a conceptual-to-empirical approach^79^, but the extracted responses did not align with the responses collected in Krpan’s taxonomy^23^. We thus shifted to an empirical-to-conceptual approach^79^, inductively classifying responses into distinct types and then iteratively grouping conceptually aligned response types into overarching themes. The response classification was completed before any meta-analyses commenced. The classification was primarily conducted by the first author, who has an interdisciplinary background in psychology, human-computer interaction, and AI-based system design. The process was supervised by two senior co-authors with expertise in psychology and human factors engineering, each of whom reviewed a random 15% subset to ensure classification reliability. In addition, we identified five categories of potential moderators to explore sources of heterogeneity in effect sizes: study characteristics, participant characteristics, partner characteristics, interaction characteristics, and response characteristics. The specific moderator variables and their coding scheme are detailed in Table 1.

**Table 1:** Moderator variables and coding scheme

Category	Moderator	Definition	Coding levels
Study characteristics	Study setting	Environment where the study was conducted	Laboratory; online; field
	Study design	Assignment of participants to human-agent and human-human interaction conditions	Between-subjects; within-subjects
	Publication year	Year the study was published	Continuous
	Publication type	Type of outlet where the study was published	Journal; conference
Participant characteristics	Sample continent	Continent where the study sample was located	Africa; Asia; Europe; North America; Oceania; South America
	Sample WEIRD	Whether the study sample was in a WEIRD or non-WEIRD country	Non-WEIRD; WEIRD
	Mean age	Mean age of participants	Continuous
	Percentage female	Percentage of female participants	Continuous
Partner characteristics	Human partner type	Type of human partner with whom participants interacted	Research team member (experimenter, research assistant, or hired actor/specialist following specific interaction scripts); participant (untrained individual); pseudo-human (ostensibly human partner controlled by algorithms); vignette-described partner (human partner and their behaviour described through vignettes)
	Agent operationalisation	How the agent partner’s behaviour was operationalised during the interaction	Autonomous (behaving independently via algorithms); Wizard-of-Oz (partially or fully controlled by hidden human operators); vignette-described
	Agent form	Whether the agent partner existed in virtual or physical form	Virtual (existing within digital interfaces); physical (tangible in the physical world)
	Agent embodiment	Whether the agent partner had a visible embodied representation	Disembodied (no visible representation, e.g., bots); embodied (visibly represented, e.g., robots and virtual humans)
	Robot appearance	Degree of human-likeness in the robot’s appearance	Non-humanoid (ABOT score 0–10); semi-humanoid (10–40); humanoid (40–70); android (70–100)
	Appearance difference	Whether the agent and human partners differed in appearance	Matched; differed
	Voice difference	Whether the agent and human partners differed in voice	Matched; differed
Interaction characteristics	Interaction realism	Whether the interaction was real-time or hypothetical	Real-time (actual or perceived real-time engagements with partners); hypothetical (vignette-based imaginative interactions)
	Interaction flow	Whether the interaction involved bidirectional or unidirectional information exchange	Bidirectional (mutual exchanges with partners); unidirectional (one-sided input)
	Interaction medium	Channel through which the interaction occurred	Face-to-face (physically co-present interaction with partners); computer-mediated (interaction with partners via a computer interface without physical co-presence); virtual reality-mediated (interaction within virtual reality environments)
	Interaction structure	Extent to which the interaction followed a predefined or scripted format	Non-structured; semi-structured; structured
	Interaction nature	Goal alignment and relational dynamics shaping exchanges between participants and partners	Neutral; cooperative; oppositional; mixed
	Interactant power symmetry	Relative power or status balance between participants and partners	Symmetrical (equal power or status, e.g., fellow game players); asymmetrical (power imbalance, e.g., customer–service employee dyad)
	Interaction task	Primary task or activity characterising the interaction	Service encounter (e.g., customer service); game interaction (e.g., economic games), instructional interaction (e.g., tutoring); communication-focused interaction (e.g., dialogues and question-answer exchanges without explicit service provision, game mechanics, or instructional objectives); motor task (e.g., physical coordination); other (unclassified)
Response characteristics	Response domain	Domain of participant responses measured in the interaction	Psychological (subjective perceptions and experiences); behavioural (observable behaviours and performance metrics)
	Measurement timing	Timing of response measurement relative to the interaction	During (e.g., in-task behaviour); after the interaction (e.g., post-interaction scale)
	Response dimension	Specific dimensions (subconstructs) of the response type	Varied by response type

Code as NA (not available) when relevant information is not reported or falls outside the predefined variable levels. Publication year was used as a proxy continuous moderator to explore potential temporal trends in partner effects, as few studies reported data collection time. Sample continent coding included Africa and South America, but no studies from these continents were identified. For sample WEIRD, WEIRD represents Western, Educated, Industrialised, Rich, and Democratic^130^. Robot appearance was coded for studies involving robots as agent partners and classified into four human-likeness levels based on the Anthropomorphic roBOT (ABOT) Database/Predictor^152^. Unlike other moderators with predefined levels, interaction task was coded inductively based on emergent themes observed across studies. Similarly, response dimension was coded where applicable; some response types were further classified into different dimensions (i.e., subconstructs), and this variation was tested as a post hoc moderator.

### Research quality assessment

We assessed the research quality of each eligible study, as its quality can bias effect sizes and higher-quality studies generally yield findings that more closely converge on the truth^80^. The first author completed quality assessment for all studies, and two co-authors each checked a random 15% subset. Any discrepancies were resolved through discussion.

Existing quality assessment tools, however, posed challenges. Widely used ones, such as the Cochrane Risk of Bias 2 (RoB-2)^81^ and Risk of Bias in Systematic Reviews (ROBIS)^82^, were developed for health-related intervention research and tailored to specific methodological designs (e.g., randomised controlled trials). These tools thus place great emphasis on assessing methodological features such as participant allocation and blinding that are less standard in human-computer interaction studies. Other popular tools are easy to apply but lack critical assessment criteria, including sample size and study design, such as the Mixed Methods Appraisal Tool (MMAT)^83^ and Joanna Briggs Institute (JBI) Critical Appraisal Checklists^84^. Alternatively, some are generalised for mixed- and multi-method studies; examples include the Quality Assessment for Diverse Studies (QuADs)^85^ and Standard Quality Assessment Criteria for Evaluating Primary Research Papers from a Variety of Fields (QualSyst)^86^, whose criteria are too broad to allow robust assessment of quantitative studies in a meta-analysis. Therefore, although developing a research quality assessment tool was not the primary goal of this review, we decided to devise a tailored checklist by adapting items from existing popular tools^81–86^ and drawing on prior work that customised quality assessment tools for their reviews^87,88^. This 23-item quality checklist is available in Supplementary Table 5.

To further test the impact of research quality on the pooled effect sizes, we accounted for its multidimensional nature rather than collapsing items into a single score^89,90^. Specifically, we computed three composite quality metrics. First, *study design rigour* reflects how thoughtfully each study was planned and designed, assessed through quality items on objectives and preregistrations, participants, and study design. Second, *data & reporting rigour* reflects how rigorously study data were handled, assessed through items on data collection, analysis, and results. Third, *broad research integrity* captures broader practices that support reproducibility and trustworthiness, assessed through items on discussion, ethics, and open science. The first two metrics directly addressed a study’s risk of bias, while the third related more to the overall research quality.

### Data analysis

All analyses were run in R v4.2.2. For each eligible study, we calculated the standardised mean difference between human-agent and human-human interaction conditions for specific human responses, serving as the effect size estimates in subsequent analyses. Hedges’ *g* was chosen over Cohen’s *d* to account for the potential bias in estimating effects with small sample sizes. Hedges’ *g* of 0.1 was interpreted as a negligible effect size, 0.2 as small, 0.5 as medium, and 0.8 as large^91^. When required statistics for calculating unadjusted effect sizes were missing or contained substantial errors, we contacted the corresponding authors to request clarification and essential statistical details or anonymised data, as per their preference. If authors did not respond or provide the requested information, we excluded those missing effects from the main analyses (and, if a study lacked all effects, we excluded that study in full). Nevertheless, when it was possible to approximate missing effects, either by estimating from reported thresholds (e.g., assuming *p* = 0.005 for *p* < 0.005), deriving statistically adjusted estimates, or imputing non-significant effects (i.e., *max*^*+*^, *max*^*–*^, and *zero*-coded^45,57^), we retained those approximated effect sizes in sensitivity analysis. Supplementary Table 6 presents all formulae for effect size calculation.

We conducted separate meta-analyses for different types of human responses, proceeding with a specific response type only when five or more studies were included^92^. We employed random-effects meta-analysis, which assumes that the different studies estimate different yet related effects^89^. The included studies differed in samples, design, and measures as expected, thus fitting this assumption. The random-effects framework accounts for both sampling error and between-study heterogeneity in effect sizes, yielding a pooled effect size that represents the mean of a distribution of true effects rather than a single fixed effect^93^. Thus, this framework allows the meta-analytic results to be generalised to a broader “population” of potential studies beyond those included in the analysis. Moreover, to accommodate dependencies between multiple effect sizes per response outcome within studies and avoid imposing the independence assumption, we employed a three-level random-effects model. Three sources of variance were identified: random sampling error (level 1), variance among effect sizes within studies (level 2), and variance among effect sizes between studies (level 3)^94^. We applied both frequentist and Bayesian approaches in the meta-analysis.

We conducted frequentist meta-analysis via the *metafor* R package^95^, employing a restricted maximum likelihood estimator to model heterogeneity and *t* distribution-based inference. We assessed effect-size heterogeneity using two indicators: (1) *Q*-statistic, which tests for the presence of heterogeneity via the significance of its p-value, and the (2) *I*^*2*^-statistic, which quantifies the magnitude of the heterogeneity. *I*^*2*^ represents the proportion of total variance among effect sizes attributable to true heterogeneity rather than sampling error, with values of 25%, 50%, and 75% interpreted as low, moderate, and high heterogeneity^96^. The presence of heterogeneity indicates the need for further moderator analysis (i.e., meta-regression) to explore potential sources of this heterogeneity. Each potential moderator was evaluated individually only when data from at least ten studies were available^89^. For categorical moderators with multiple conditions, only conditions represented by at least three studies were included^97^. Meta-regression was also employed to test whether research quality systematically impacted the pooled effect size, with three quality metrics evaluated individually. Subgroup analyses were conducted when meta-regression yielded a significant moderating effect, and were visualised using orchard plots via the *orchaRd* R package^98^. Additionally, we checked publication bias for response types with at least ten studies^89^ by visually inspecting funnel plots for asymmetry and performing Egger Sandwich tests via the *clubSandwich* R package^99^, with regression slope significance serving as a statistical indicator of asymmetry. We further conducted sensitivity analysis for outliers (effect sizes with absolute studentised deleted residuals exceeding 1.96^100^) and influential cases (identified using Cook’s distance, DFBETAS values, and hat values^95^), re-running all meta-analyses after removing these cases. This evaluated whether results were sensitive to potential deviations from underlying distributional assumptions^89^, thereby assessing the robustness of pooled effect sizes.

To complement the traditional frequentist meta-analytic approach, we also conducted Bayesian meta-analysis via the *brms* and *bridgesampling* R packages^101,102^. Bayesian analysis allows incorporation of prior information to better estimate different sources of variance and enables direct probability statements about parameters via credible intervals^103^. We pre-determined prior distributions for effect size estimates and heterogeneity parameters. For effect sizes, we used a Cauchy(0, 1/√2) distribution, which has become a default prior in the field of psychology^104^, to reflect uncertainty about both the direction and magnitude of effect sizes when comparing responses in human-agent vs. human-human interactions. For between-study variance, we used an inverse-Gamma(1, 0.15) distribution, based on between-study heterogeneity estimates from meta-analyses reported in *Psychological Bulletin* from 1990–2013^104,105^. For within-study variance, we used an inverse-Gamma(1, 0.1) distribution, reflecting a priori expectation that variance among effect sizes within studies is smaller than variance between studies. We used a Cauchy(0, 1/√2) distribution for moderator effects. We also assessed the sensitivity of Bayesian meta-analytic results to prior distributions by using the following alternatives. For effect sizes, we considered a Student-*t*(3, 0, 1) prior, a weakly informative distribution that places less density at zero and more on moderate-to-large effects and has lighter tails than Cauchy(0, 1/√2). We considered half-Cauchy(0, 0.3), a commonly used weakly informative prior^106^, for between-study variance and half-Cauchy(0, 0.2) for within-study variance. Furthermore, we performed Markov Chain Monte Carlo diagnostics to assess model validity: potential scale reduction statistics $\left(\hat{R}\le 1.01\right)$ for convergence^107^, and effective sample sizes (ESS ≥ 1,000) for all parameters for sampling efficiency^101^. In the Bayesian approach, prior distributions constituted explicit modelling assumptions; sensitivity analyses and convergence diagnostics indicated that these assumptions were appropriate for our data.

In addition, the Bayesian approach provides formal measures (i.e., Bayes factors; BF_10_) of the strength of evidence for the study hypothesis (H_1_) relative to the null hypothesis (H_0_), thereby indicating when pooled effects should be interpreted with caution^91^. Specifically, BF_10_ < 1 indicates the data are more supportive of H_0_ than H_1_, with values of 1/3–1 indicating ambiguous evidence, 1/10–1/3 substantial evidence, 1/30–1/10 strong evidence, 1/100–1/30 very strong evidence, and < 1/100 decisive evidence in support of H_0_; BF_10_ = 1 indicates perfect ambiguity; BF_10_ > 1 indicates the data are more supportive of H_1_, with values of 1–3, 3–10, 10–30, 30–100, and > 100 indicating ambiguous, substantial, strong, very strong, and decisive evidence in support of H_1_, respectively^108^.

### Ethics statement

This systematic review and meta-analysis is based on data extracted from previously published studies. Where necessary, additional anonymised data were requested and obtained ethically and legally from the original study authors. No new data were collected from human participants. Thus, no ethical approval was required in accordance with the institutional ethical guidelines.

## Results

**Fig. 2:**
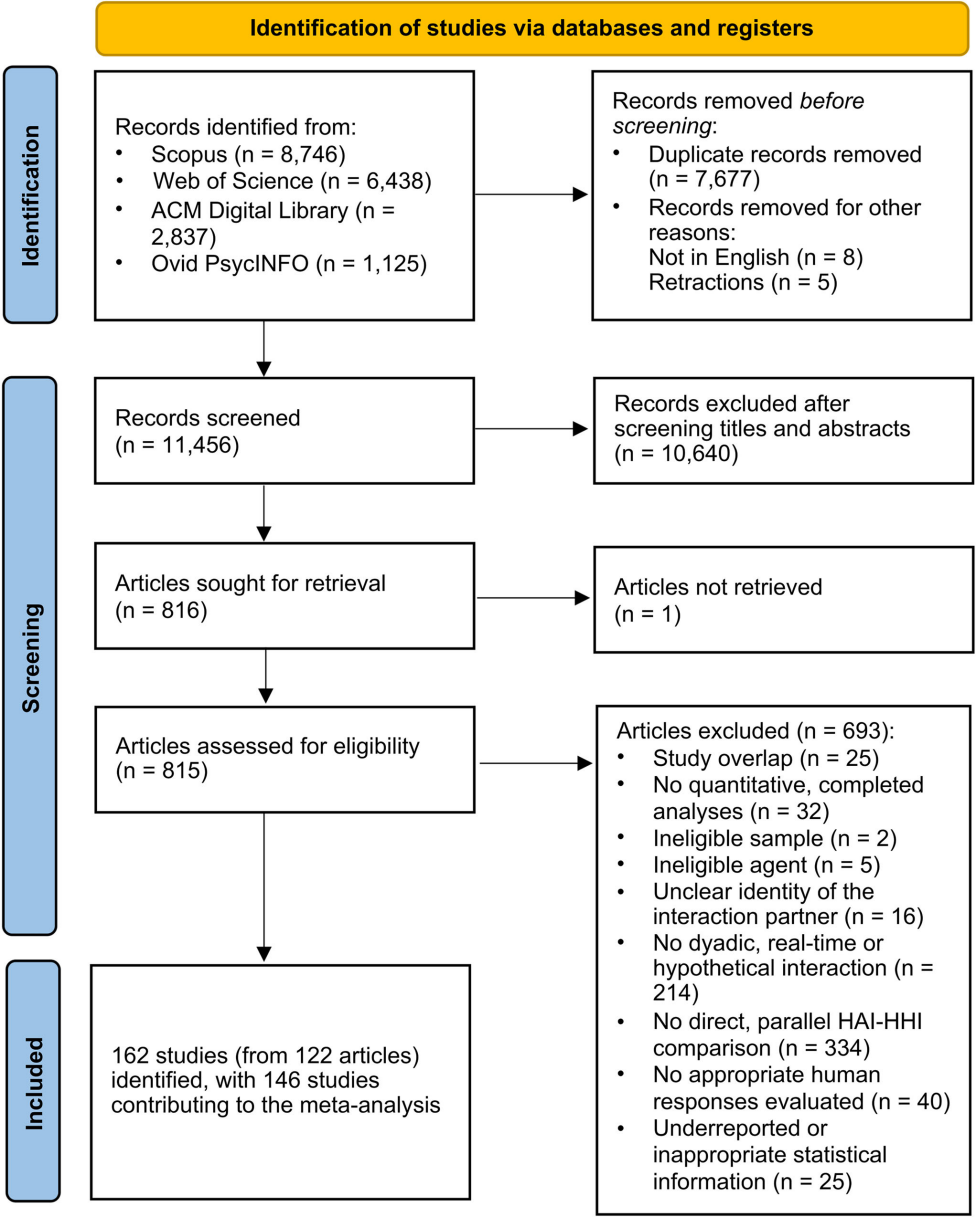
PRISMA flowchart. Two articles^165,166^ reported the same underlying study while analysing different human responses, and were treated as one study in our meta-analysis. Articles^167,168^ likewise reported the same study and were treated as one. There was one article^169^ describing a single investigation but collecting and analysing data separately for Chinese and US samples; to keep sample independence, we treated these as two studies. Another article^170^ reported Japanese and US samples, which we also treated as two studies. In addition, of the 25 articles excluded for underreported or inappropriate statistical information, 16 articles (22 studies) allowed deriving approximated effect sizes for some or all responses examined. These approximated effect sizes were included in the sensitivity analyses in the supplementary information.

The study selection process is summarised in the PRISMA flowchart (Fig. 2). After title-abstract screening (11,456 records) and full-text screening (815 articles), 162 studies (from 122 articles) met our eligibility criteria. Key characteristics of eligible studies are provided in Supplementary Table 7. These studies investigated a broad set of psychological and behavioural responses, which we classified into distinct response types and meta-analysed separately. Sixteen studies examined rare responses with insufficient data for meta-analysis^92^, and these were narratively synthesised in Supplementary Table 8. The final quantitative synthesis included 146 studies (from 112 articles), yielding 468 effect sizes (3.21 per study).

Integrating frequentist and Bayesian approaches, we conducted a series of meta-analyses comparing individuals’ psychological and behavioural responses in dyadic interactions with performance-matched agent vs. human partners. Studies included in the meta-analyses were published between 2003 and 2024 across 112 publications across diverse outlets. Most publications (85) were journal articles, with *Computers in Human Behavior* (10) and *International Journal of Social Robotics* (8) among the more frequently represented outlets. The remaining 27 appeared in conference proceedings, with the ACM International Conference on Intelligent Virtual Agents (IVA; 4) and the IEEE International Conference on Robot and Human Interactive Communication (RO-MAN; 4) being relatively common venues.

Of 146 included studies, the average sample size was 162.80 (*SD* = 163.13, median = 116.17, range = 8–945). For seven studies that reported only total sample sizes and included conditions beyond human-agent and human-human interactions, we assumed equal condition sizes^109,110^; removing these yielded a similar average of 164.60 (*SD* = 166.21, median = 117). Using sample-size weighting, the mean participant age was 31.62 years (range = 18.60–78.79), and the mean percentage of female participants was 56.87% (range = 0–100%). The study sample was geographically diverse, while Western countries and East Asia were disproportionately represented. Most studies were conducted in the USA (50) and China (18), followed by Japan (12), Germany (8), Italy (8), and the UK (5). Other locations included France (2), Finland (2), and several countries represented by one study each (Australia, Belgium, Canada, New Zealand, Singapore, South Korea, Sweden, and the Netherlands). Six studies involved multi-country samples, and 27 studies did not report location information.

### RQ1: Which psychological and behavioural responses have been investigated in studies comparing human-agent and human-human interactions?

We identified 23 types of human responses, each with at least five studies for meta-analysis^92^. They were grouped into six themes: prosociality and morality, social perceptions of interaction partners, trust in interaction partners, social alignment with interaction partners, personal agency and task performance, and interaction experiences. An overview of the response types, grouped under these themes and with detailed conceptualisations, is provided in Table 2.

**Table 2:** Overview of the meta-analysed response types

Response theme	Response	Conceptualised As
Prosociality and morality	Prosocial behaviour	Voluntary actions that are intended to help or benefit others^111^.
	Moral engagement	Psychological and behavioural commitment to moral standards in a given context, demonstrating direct or indirect responsiveness to the needs and interests of others^112,113^.
Social perceptions of interaction partners	Perceived social presence	Subjective experience of being present with a real social partner, with whom one can exchange thoughts and emotions^56,153^.
	Perceived likeability	Positive evaluation of the partner based on their affiliative capacity and social attractiveness^115,116^.
	Perceived competence	Evaluation of the partner’s intelligence and domain-specific knowledge, or their effectiveness and efficiency in task performance^115,117^.
	Agency attribution	Explicit, reflective process of ascribing the partner the capacity to intentionally initiate events, and recognising them as the cause of their own decisions or actions^118^.
	Responsibility attribution	Process of assigning responsibility, with credit or blame, for an event or action to the partner^119^. It is closely related to agency attribution, which lays the foundation: the partner needs to be perceived as having agency to cause an outcome before being assigned responsibility for it^154,155^.
Trust in interaction partners	Behavioural trust	Observable actions of voluntarily accepting vulnerability based on positive expectations of the partner, such as risk-taking behaviour or reliance on the partner under uncertainty^156,157^.
	Subjective trust	Psychological state comprising the intention to accept vulnerability based on positive expectations of the partner, such as their ability, benevolence, or integrity^120,157^.
Social alignment with interaction partners	Social alignment	Voluntary alignment of internal states and behaviours with the partner, including self-other integration (merging identities and perspectives), advice taking (incorporating partner insights), synchrony (adapting verbal and nonverbal behaviours), and proximity regulation (regulating interpersonal distances)^121^.
Personal agency and task performance	Perceived self-agency	Subjective experience of controlling one’s own body and external events, which leads one to feel responsible for what their decisions or actions cause^158^.
	Self-disclosure	Process of revealing personal information, such as one’s own thoughts, feelings, and experiences, with the partner^159^.
	Strategic economic behaviour	Deliberate choices of actions in economic games where one, recognising the interdependence of actions, anticipates and reacts to the partner’s actions by weighing how their choices would affect both personal and partner outcomes. It can be affected by multiple factors, including not only payoff maximisation, but also social preferences (e.g., reciprocity, trust, and fairness) or even intuitions^123,160^.
	Objective task performance	Measurable and quantifiable assessments regarding one’s performance of specific tasks.
Interaction experiences	Perceived partner relational qualities	Subjective evaluations of the relational attributes the partner exhibits during interaction, including perceived rapport (closeness and mutual connection), perceived interactivity (active engagement and responsiveness), perceived empathy and supportiveness (understanding of and support for one’s perspectives and feelings), and perceived customer orientation (commitment to meeting one’s needs)^124^.
	Affective valence	Hedonic tone (i.e., unpleasantness–pleasantness) of the emotional experience when interacting with the partner^161^.
	Affective arousal	Activation level (i.e., calmness–excitement) of the emotional experience when interacting with the partner^161^.
	Interaction satisfaction	Overall evaluation of how well the interaction with the partner meets one’s needs and expectations^125^.
	Future interaction intention	Degree to which one would like to interact with the partner again in the future^162^.
	Perceived interaction naturalness	Degree to which one perceives their interaction with the partner as natural.
	Perceived interaction enjoyment	Degree to which one feels they have enjoyed interaction with the partner.
	Subjective workload	Perception and emotional experience of the overall effort invested in performing specific tasks^163^.
	Subjective task engagement	Perception and emotional experience of involvement and investment in performing specific tasks^164^.

To derive response types and themes, we performed a posteriori classification of the diverse human responses investigated across studies. After a conceptual-to-empirical approach^79^ to classification development failed to align with extracted responses (Supplementary Note 1), we adopted an empirical-to-conceptual approach^79^, allowing the classification scheme to arise directly from the dataset. We reviewed the descriptions and measures of all human responses extracted from the studies and inductively classified them into distinct response types. Conceptually aligned response types were further grouped into six identified themes, with a residual category for unclassified responses.

### RQ2: Which specific response types differ between human-agent and human-human interactions?

**Fig. 3:**
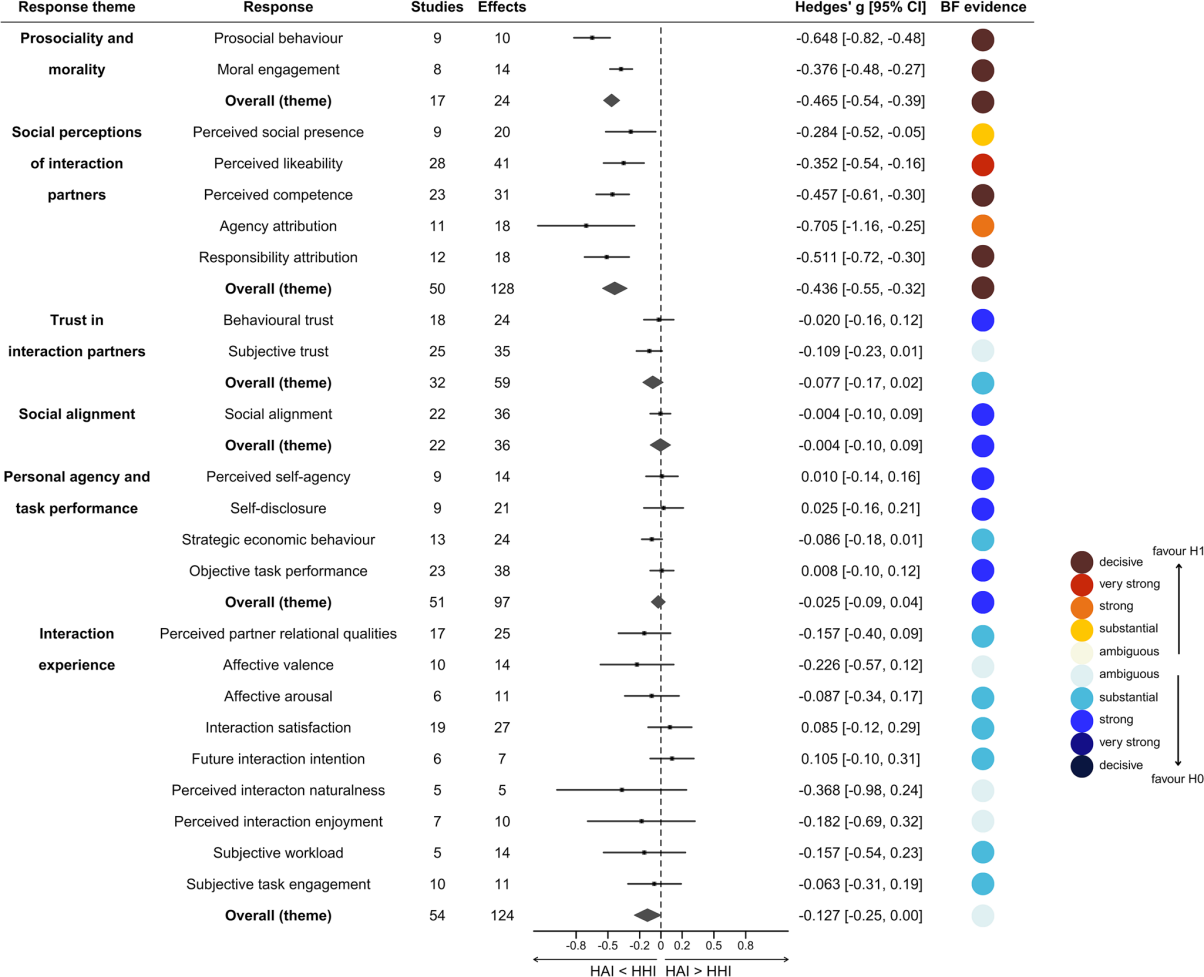
Forest plot visualising pooled effect sizes for different response types. Only response types with five or more studies were included in the meta-analysis and are shown in this plot. Horizontal bars represent 95% CIs. Response types with bars that do not cross the dashed vertical “line of no effect” indicate existing significant differences between human-agent and human-human interactions. Ticks on the x-axis denote conventional thresholds for small (*g* = 0.2), medium (*g* = 0.5), and large (*g* = 0.8) effects. The pooled effect sizes shown in this plot were derived from frequentist meta-analyses, with the accompanying strength of Bayesian evidence indicated by coloured circles. To aid interpretation, effect sizes were also pooled at the theme level and shown as diamonds. For the response theme comprising one response type (i.e., social alignment), the response-level estimate also represents the theme-level pooled effect. Full theme-level meta-analytic results are presented in Supplementary Table 17.

Meta-analyses revealed notable differences in prosociality and morality, as well as in social perceptions of partners, between human-agent and human-human interactions. Detailed results are summarised in Table 3, with pooled effect sizes visualised in Fig. 3.

**Table 3:** Meta-analytic results for different response types in human-agent vs. human-human interactions

Response theme	Response	Hedges’ *g*	95% CI	*t*	*t_p*	*Q*	*Q_p*	*I*^*2*^ (%)	BF_10_	*k*	*m*
Prosociality and morality	Prosocial behaviour	−0.648	[−0.82, −0.48]	−8.66	< 0.001	10.83	0.288	21.78	6003.065	9	10
	Moral engagement	−0.376	[−0.48, −0.27]	−8.19	< 0.001	4.04	0.991	0	1725.850	8	14
Social perceptions of interaction partners	Perceived social presence	−0.284	[−0.52, −0.05]	−2.81	0.023	48.72	< 0.001	68.19	3.495	9	20
	Perceived likeability	−0.352	[−0.54, −0.16]	−3.78	< 0.001	443.07	< 0.001	88.39	41.417	28	41
	Perceived competence	−0.457	[−0.61, −0.30]	−6.21	< 0.001	148.84	< 0.001	77.56	10296.030	23	31
	Agency attribution	−0.705	[−1.16, −0.25]	−3.43	0.006	229.52	< 0.001	94.62	16.554	11	18
	Responsibility attribution	−0.511	[−0.72, −0.30]	−5.44	< 0.001	92.77	< 0.001	81.56	512.117	12	18
Trust in interaction partners	Behavioural trust	−0.020	[−0.16, 0.12]	−0.30	0.769	47.98	0.002	60.83	0.077	18	24
	Subjective trust	−0.109	[−0.23, 0.01]	−1.88	0.073	115.41	< 0.001	76.87	0.358	25	35
Social alignment	Social alignment	−0.004	[−0.10, 0.09]	−0.08	0.937	52.90	0.027	33.11	0.052	22	36
Personal agency and task performance	Perceived self-agency	0.010	[−0.14, 0.16]	0.16	0.880	18.51	0.139	37.53	0.084	9	14
	Self-disclosure	0.025	[−0.16, 0.21]	0.31	0.766	28.03	0.109	44.89	0.089	9	21
	Strategic economic behaviour	−0.086	[−0.18, 0.01]	−1.93	0.078	32.82	0.084	32.26	0.246	13	24
	Objective task performance	0.008	[−0.10, 0.12]	0.15	0.884	70.60	< 0.001	46.39	0.062	23	38
Interaction experiences	Perceived partner relational qualities	−0.157	[−0.40, 0.09]	−1.36	0.194	159.82	< 0.001	91.66	0.320	17	25
	Affective valence	−0.226	[−0.57, 0.12]	−1.49	0.171	84.45	< 0.001	86.33	0.410	10	14
	Affective arousal	−0.087	[−0.34, 0.17]	−0.87	0.422	17.81	0.058	46.56	0.168	6	11
	Interaction satisfaction	0.085	[−0.12, 0.29]	0.86	0.400	281.59	< 0.001	94.73	0.154	19	27
	Future interaction intention	0.105	[−0.10, 0.31]	1.32	0.245	14.10	0.028	57.34	0.212	6	7
	Perceived interaction naturalness	−0.368	[−1.24, 0.50]	−1.18	0.304	73.40	< 0.001	96.97	0.585	5	5
	Perceived interaction enjoyment	−0.182	[−0.69, 0.32]	−0.88	0.411	83.97	< 0.001	89.06	0.355	7	10
	Subjective workload	−0.157	[−0.54, 0.23]	−1.13	0.323	32.49	0.002	69.04	0.320	5	14
	Subjective task engagement	−0.063	[−0.31, 0.19]	−0.57	0.585	20.85	0.022	58.66	0.168	10	11

For simplicity, this table presents results primarily from frequentist meta-analysis. BF_10_ is presented to provide complementary Bayesian evidence for H_1_ over H_0_. All frequentist estimates are consistent with Bayesian estimates. Full Bayesian results are presented in Supplementary Table 16. Specifically, Hedges’ *g*, 95% CI, *t*-value and associated *p*-value were estimated via random-effects meta-analysis. *Q* is Cochrane’s *Q*-statistic for testing heterogeneity. *I*^*2*^ is the proportion of total variance attributable to true heterogeneity rather than sampling error. *k* is the number of studies including in meta-analysis. *m* is the number of effect sizes included.

#### Prosociality and morality

*Prosocial behaviour* refers to voluntary actions intended to benefit others^111^, with frequentist meta-analysis revealing a medium-to-large partner effect. Individuals behaved significantly less prosocially when interacting with agent vs. human partners, for example by sharing less in dictator games or adapting less to the partner’s perspective in joint tasks. *Moral engagement*, the psychological and behavioural commitment to moral standards^112,113^, showed a small-to-medium partner effect. Individuals engaged significantly less morally with agent vs. human partners, with reduced moral acts, intentions, or feelings of guilt.

#### Social perceptions of interaction partners

On average, *perceived social presence*—the sense of the partner being “there” and socially real—showed a small partner effect, with agents perceived as significantly less socially present than human partners. For two core dimensions of social perception^114^, *perceived likeability* reflects affective evaluations of the partner^115,116^ (e.g., warmth, friendliness), while *perceived competence* captures evaluations of capability^115,117^ (e.g., intelligence, effectiveness). Both showed small-to-medium partner effects, with agents perceived as significantly less likeable and competent than human partners. The agent disadvantage was greater for higher-order social constructs: a medium-to-large partner effect on *agency attribution*—capacity for intentional action^118^—and a medium effect on *responsibility attribution*—accountability for outcomes^119^, with agents attributed significantly less agency and responsibility.

Bayesian meta-analytic results were consistent with frequentist estimates. Bayesian support was decisive for partner effects on prosocial behaviour and moral engagement. Regarding social perceptions, support was substantial for partner effects on perceived social presence, strong for agency attribution, very strong for perceived likeability, and decisive for perceived competence and responsibility attribution.

### RQ3: Which specific response types are similar between human-agent and human-human interactions?

Meta-analyses revealed general response similarities between human-agent and human-human interactions across four response themes: trust, social alignment, perceived agency and task performance, and interaction experiences. Detailed results are provided in Table 3 and Fig. 3.

#### Trust in interaction partners

Trust is “a willingness to be vulnerable” in the absence of the ability to monitor the trustee^120^, encompassing both behavioural and psychological aspects. Frequentist meta-analyses revealed no significant differences in *behavioural trust* (e.g., risk-taking or reliance on the partner under uncertainty) and *subjective trust* (e.g., perceived trustworthiness or reliability) towards agent vs. human partners.

#### Social alignment with interaction partners

*Social alignment* refers to the voluntary alignment of internal states and behaviours with the partner^121^, such as self-other integration and behavioural synchrony. Like behavioural trust, it is critical for interaction coordination^121^ and showed no significant difference with agent vs. human partners.

#### Personal agency and task performance

*Perceived self-agency* and *self-disclosure* are self-oriented processes^122^ during interaction. Self-agency is the sense of control over one’s own body and external events, while self-disclosure involves sharing personal information. Both showed no significant differences during agent vs. human interactions. Moreover, *strategic economic behaviour*—deliberate choices in economic games while acknowledging the interdependence of players’ actions^123^—did not differ significantly by partner type. *Objective task performance*—quantifiable assessments of task execution (e.g., accuracy or response time)—also did not differ significantly.

#### Interaction experiences

On average, *perceived relational qualities* of agent vs. human partners—subjective evaluations of relational attributes the partner exhibits during interaction^124^, such as rapport and empathy—did not differ significantly. Likewise, no significant differences were found for *affective valence* (e.g., unpleasantness–pleasantness) and *arousal* (e.g., calmness–excitement). *Interaction satisfaction*—overall evaluation of how well the interaction meets one’s needs and expectations^125^—did not differ significantly by partner type, nor did *future interaction intention* (e.g., willingness to engage again), *perceived naturalness*, or *enjoyment of interaction*. For subjective task experience, *subjective workload* (e.g., perceived effort) and *task engagement* (e.g., enjoyment or immersion in the task) did not differ significantly.

Bayesian meta-analytic results were consistent with frequentist estimates. However, Bayesian evidence was ambiguous for the absence of partner effects on subjective trust, affective valence, perceived interaction naturalness, and enjoyment. For other response types, evidence for the absence of partner effects was clearer. Bayesian support was strong for behavioural trust, social alignment, perceived self-agency, self-disclosure, and objective task performance, and substantial for strategic economic behaviour and most interaction experiences, including perceived partner relational qualities, interaction satisfaction, future interaction intention, affective arousal, subjective workload, and task engagement.

### RQ4: To what extent do study, participant, partner, interaction, and response characteristics moderate partner effects on different response types?

We conducted univariate meta-regressions exploring characteristics of studies, participants, partners, interactions, and responses as moderators (see Methods for moderator coding). Of 23 response types meta-analysed, moderator analyses were not conducted for partner effects on prosocial behaviour, moral engagement, perceived self-agency, self-disclosure, strategic economic behaviour, and affective arousal due to non-significant heterogeneity in effect sizes (*Q*-test *p* < 0.05). Despite significant effect-size heterogeneity, the limited number of studies (*k* < 10)^89^ precluded moderator analyses for perceived social presence, future interaction intention, perceived interaction naturalness and enjoyment, and subjective workload. Therefore, we conducted moderator analyses for 12 response types: perceived likeability, perceived competence, agency attribution, responsibility attribution, behavioural trust, subjective trust, social alignment, objective task performance, perceived partner relational qualities, affective valence, interaction satisfaction, and subjective task engagement.

For simplicity, this section only presents results for six response types under the themes of “social perceptions of interaction partners” and “interaction experiences” (Tables 4 and 5), with moderators both reaching frequentist significance and receiving at least substantial Bayesian support (*p* < 0.05; BF_10_ > 3). Full results are provided in Supplementary Table 9.

**Table 4:** Meta-regression and subgroup analysis results for social perceptions of interaction partners

Response	Meta-regression	Subgroup analysis
Perceived likeability	Appearance difference: *F* = 7.48, *p* = 0.011; BF_10_ = 4.559	
	Interaction task: *F* = 5.16, *p* = 0.007; BF_10_ = 4.839	
Perceived competence	Agent form: *F* = 13.42, *p* = 0.001; BF_10_ = 16.598	
	Appearance difference: *F* = 12.49, *p* = 0.002; BF_10_ = 19.646	
	Interaction medium: *F* = 12.08, *p* = 0.002; BF_10_ = 14.841	
Agency attribution	Interaction medium: *F* = 7.09, *p* = 0.026; BF_10_ = 4.331	
Responsibility attribution	Study setting: *F* = 11.34, *p* = 0.007; BF_10_ = 4.271	
	Interaction realism: *F* = 11.34, *p* = 0.007; BF_10_ = 4.271	

Moderator analyses were conducted for response types with significant effect-size heterogeneity and at least ten available studies. Potential moderators were tested individually. For categorical moderators with multiple conditions, only conditions represented by at least three studies were included. For simplicity, this table presents results only for moderators that both reached frequentist significance and received at least substantial Bayesian support (*p* < 0.05; BF_10_ > 3). Full results for all tested moderators are available in Supplementary Table 9. For meta-regression results, *F*-value is from random-effects meta-regression. BF_10_ represents the Bayesian evidence for H_1_ (i.e., presence of a moderating effect) over H_0_ (i.e., absence of a moderating effect). For follow-up subgroup analyses, Hedges’ *g* and 95% CI were estimated via random-effects meta-analyses and visualised via orchard plots. *k* is the number of studies including in the analysis. *m* is the number of effect sizes included.

**Table 5:** Meta-regression and subgroup analysis results for interaction experiences

Response	Meta-regression	Subgroup analysis
Perceived partner relational qualities	Agent operationalisation:*F* = 11.37, *p* = 0.001; BF_10_ = 71.227	
	Human partner type:*F* = 10.75, *p* = 0.002; BF_10_ = 45.469	
	Interaction realism:*F* = 23.50, *p* < 0.001; BF_10_ = 298.645	
	Response dimension:*F* = 6.78, *p* = 0.006; BF_10_ = 3.334	
Interaction satisfaction	Interaction nature:*F* = 40.31, *p* < 0.001;BF_10_ = 15137.576	

The same note as in Table 4 applies.

#### Social perceptions of interaction partners

For *perceived likeability*, appearance difference (matched vs. differed) and interaction task (service encounter vs. game vs. instructional interaction vs. communication-focused) moderated the partner effect. Agents were perceived as significantly less likeable than human partners (a medium partner effect) when appearance differed, but this was non-significant when appearances matched. Moreover, agents were significantly less likeable than human partners in service encounters and games (medium and high partner effects, respectively), but this was non-significant in instructional interactions and communication-focused interactions.

For *perceived competence*, appearance difference (matched vs. differed), agent form (physical vs. virtual), and interaction medium (face-to-face vs. computer-mediated) moderated the partner effect. Agents were perceived as significantly less competent than human partners (a medium-to-large partner effect) when appearance differed, but this was non-significant when appearances matched. The agent disadvantage manifested across conditions of agent form and interaction medium but was significantly greater for physical agents than virtual agents (medium-to-large vs. small) and greater in face-to-face than computer-mediated interactions (medium-to-large vs. small).

For *agency attribution*, interaction medium (face-to-face vs. computer-mediated) moderated the partner effect. Agents were attributed significantly less agency than human partners (a large partner effect) in face-to-face interactions; this partner effect was small-to-medium while non-significant in computer-mediated interactions.

For *responsibility attribution*, study setting (online vs. lab) and interaction realism (hypothetical vs. real-time)—which were perfectly co-linear—moderated the partner effect. In online studies with hypothetical interaction, agents were attributed significantly less responsibility than human partners (a medium-to-large partner effect), whereas lab studies with real-time interaction yielded a non-significant partner effect.

#### Interaction experiences

For *perceived partner relational qualities*, agent operationalisation (vignette-described vs. Wizard-of-Oz vs. autonomous), human partner type (vignette-described partner vs. research team member vs. algorithm-controlled pseudo-human), and interaction realism (hypothetical vs. real-time) moderated the partner effect. Perfect multicollinearity occurred among moderator conditions due to a subset of studies that asked participants to imagine interacting with partners (i.e., hypothetical interaction) through vignettes. This subset yielded a medium-to-large partner effect, reflecting methodological contribution to agent disadvantage. Studies with real-time interaction yielded a non-significant partner effect, regardless of agent operationalisation or human partner type. Additionally, response dimension moderated this partner effect. Studies measuring perceived customer orientation showed that agents were perceived as significantly less customer-oriented than human partners (a medium-to-large partner effect), whereas studies of other relational qualities—perceived rapport, interactivity, and empathy—yielded non-significant partner effects.

For *interaction satisfaction*, interaction nature (oppositional vs. cooperative) moderated the partner effect. In oppositional interactions, satisfaction was significantly higher with agent vs. human partners (a medium partner effect), whereas in cooperative interactions, this was non-significant.

In brief, no participant characteristics demonstrated robust moderating effects (i.e., effects reaching frequentist significance with at least substantial Bayesian support). Nevertheless, significant moderators of partner effects on specific response types were identified across other categories: study characteristics (study setting), response characteristics (response dimension), partner characteristics (appearance difference, agent form, agent operationalisation, and human partner type), as well as interaction characteristics (interaction task, medium, realism, and nature).

### Publication bias, research quality, and sensitivity analysis

For response types with at least ten studies^89^, we evaluated publication bias via funnel plots and Egger Sandwich tests^126^. Visual inspection of funnel plots showed fairly symmetric distributions for most response types (Supplementary Fig. 1), which were also supported by non-significant Egger tests (Supplementary Table 10). Thus, the risk of publication bias was minimal. There were two exceptions: social alignment (Egger *p* = 0.018, with its funnel showing a missing left wedge and right-skewed clustering of smaller studies) and objective task performance (Egger *p* = 0.038, despite a relatively symmetric funnel). Nevertheless, given near-zero pooled effect sizes and mostly non-significant individual effects for both response types, the observed asymmetry likely reflected small-study effects (i.e., small samples are associated with greater random error and yield larger yet mostly non-significant effect estimates with wide confidence intervals, resulting in apparent asymmetry) rather than true publication bias. Research quality was assessed via a tailored checklist, yielding three quality metrics—study design rigour, data & reporting rigour, and broad research integrity—for each study (Supplementary Table 11). Meta-regressions showed no systematic impact of research quality on our results, though modest influences of specific quality metrics cannot be ruled out; details are provided in Supplementary Table 12. Finally, three sensitivity checks—incorporating approximated effect sizes, removing outliers and influential cases, and using alternative Bayesian priors—confirmed that our main meta-analytic results were robust across analytic decisions; details are provided in Supplementary Tables 13–15.

## Discussion

This review synthesised empirical evidence on similarities and differences in individuals’ psychological and behavioural responses when interacting with performance-matched agent vs. human partners. We conducted separate random-effects meta-analyses for 23 response types, followed by univariate meta-regressions for 12 response types with significant effect-size heterogeneity and at least ten available studies.

### RQ1: Which psychological and behavioural responses have been investigated in studies comparing human-agent and human-human interactions?

In total, we identified 23 types of psychological and behavioural responses across six themes, reflecting the breadth of human responses examined in human-agent and human-human interaction research. However, the distribution of response types across studies was uneven. Perceived likeability and competence, subjective trust, social alignment, and objective task performance were among the most frequently investigated response types. In contrast, certain interaction experiences, including affective arousal, future interaction intention, perceived interaction naturalness and enjoyment, and subjective workload, were compared less frequently in human-agent vs. human-human interactions.

Responses within the themes of “social perceptions of interaction partners” and “interaction experiences” were particularly diverse, with many lacking sufficient data for meta-analysis. They were measured using heterogeneous instruments that captured different aspects of constructs at varying levels of abstraction, contributing to wider confidence intervals in pooled effect sizes. For example, interaction satisfaction has been assessed with measures ranging from single- to multi-item scales, raising concerns about their psychometric comparability. This aligns with a previous review that identified a continued tendency to develop bespoke questionnaires rather than adopt validated ones^127^. Notably, less empirical research has examined “negative” human responses, despite large-scale self-report evidence showing a range of negative responses to agents^23^. Greater attention to responses such as hostility, blame, and distrust is needed, as real-world interactions are not uniformly positive, and these reactions may be distinct from, rather than the inverse of, positive responses.

### RQ2: Which specific response types differ between human-agent and human-human interactions?

Our meta-analyses found that individuals were less prosocial and morally engaged when interacting with agents than with human partners. These results quantitatively substantiate and extend a systematic review showing increased selfishness and rationality when playing behavioural economic games with computer vs. human partners^128^, suggesting that reduced prosocial behaviour and moral engagement with agents generalises beyond economic paradigms to broader interaction contexts.

Compared to human partners, agents were on average perceived as possessing fewer social attributes: they were rated as less likeable and competent, and attributed less agency and responsibility. Agents were also perceived as having lower social presence. However, this partner effect was small and sensitive to how missing non-significant effects were handled, necessitating further research to reach robust conclusions regarding perceived social presence. This pattern aligns with prior meta-analytic findings that agent anthropomorphism was more strongly associated with user perceptions such as likeability and intelligence than with social presence^72^. Overall, our review provides quantitative support for theoretical accounts that current intelligent agents lack key social affordances necessary to cultivate fully human-like relationships^27^.

### RQ3: Which specific response types are similar between human-agent and human-human interactions?

Alongside response differences, we identified key response similarities between human-agent and human-human interactions. Specifically, individuals demonstrated social alignment and behavioural trust with agent and human partners comparably. Subjective trust likewise showed no significant difference in frequentist meta-analysis, whereas weak Bayesian support for the null effect leaves open the possibility of a modest partner effect. Previous quantitative syntheses demonstrated positive effects of anthropomorphism on trust attitudes towards agents^73,74^. However, these syntheses included many studies in which participants evaluated agents without interacting with them. Trust formed in evaluative paradigms may reflect initial impressions rather than trust perceptions and behaviours that develop through interaction. Our work extends this literature by demonstrating that trust does not reliably differ between two interactions, consistent with a prior review proposing that trust in agent and human partners develops through similar underlying processes^59^. In addition, individuals exhibited comparable self-agency, self-disclosure, and strategic economic behaviour in both interactions. Objective task performance was likewise comparable, in line with a meta-analysis of agent anthropomorphism showing no significant effect on participants’ task performance^74^.

Most interaction experiences were on average similar between partner types. Bayesian support for no partner effects was ambiguous for affective valence and perceived interaction naturalness and enjoyment. Therefore, modest partner effects for these three responses cannot be ruled out. Echoing this uncertainty, prior meta-analyses regarding affective valence have shown mixed findings: one synthesis reported a significant effect of agent anthropomorphism on overall affective valence^74^, while others found significant associations with positive affect but not negative affect^72,73^.

### RQ4: To what extent do study, participant, partner, interaction, and response characteristics moderate partner effects on different response types?

Many subjective responses (i.e., social perceptions of partners, subjective trust, and interaction experiences) exhibited high effect-size heterogeneity, indicating that the average effects from meta-analyses should not be interpreted as universal, with true partner effects being context-dependent. Across examined social attributes, agents were on average perceived less positively than human partners, but several moderators shaped these partner effects. Specifically, the partner effect on perceived likeability disappeared when partner appearance matched (vs. differed) or in instructional/communication-focused interaction (vs. game/service encounter) tasks. The partner effect on perceived competence disappeared when partner appearance matched (vs. differed) and was reduced when agents were virtual (vs. physical) or in computer-mediated (vs. face-to-face) interactions. Meta-analyses on agent anthropomorphism and social cues also reported positive associations with user perceptions, including likeability and intelligence^72–74^, suggesting that these agent disadvantages relate to design cues rather than inherent agent limitations. In addition, partner effects on agency and responsibility attribution disappeared in computer-mediated (vs. face-to-face) interactions and in lab-setting, real-time (vs. online-setting, hypothetical) interactions, respectively.

Given the limited studies for many interaction experiences, moderators were identified only for interaction satisfaction and perceived partner relational qualities. Interaction satisfaction was higher with agents than with human partners in oppositional (vs. cooperative) interactions. Agents were perceived as having lower relational qualities than human partners in hypothetical, vignette-described (vs. real-time) interactions, or when focusing on customer orientation (vs. interactivity/rapport/empathy). Of note, rapport did not differ significantly between agent and human partners in our review. This adds to existing mixed meta-analytic evidence, with one synthesis finding no significant association between anthropomorphism and rapport with agents^72^, while another finding a significant effect of human-like social cues on rapport^73^. For many other tested moderators, evidence regarding their influence was inconclusive—for example, some reached frequentist significance but with weak Bayesian support (e.g., measurement timing for subjective trust). These inclusive results highlight the need for further research to clarify variations in these subjective responses. Overall, we believe the partner effects on these responses should be interpreted by considering both the average effects and the strong effect-size heterogeneity.

### Limitations

These findings should be interpreted considering the review limitations. First, despite all studies being peer-reviewed, research quality varied greatly, with three quality metrics averaging only moderate levels. While we found no systematic impact of research quality on results, missing or inconsistent statistics and methodological details in some studies could add noise to certain meta-analytic estimates. Second, our response classification was derived inductively from the variables and measures reported in the included studies and thus cannot capture the full spectrum of human responses possible in interactions with agent and/or human partners. Third, we restricted moderator analyses to univariate models. Multivariate models, even Bayesian regularised meta-regression^129^, would be uncertain due to a lack of sufficient studies to accommodate multiple moderators simultaneously. Additionally, many subjective responses exhibited high effect-size heterogeneity, which our meta-regressions only partly explained. Measurements used across studies varied in format, reliability, and validity, but they were too diverse to be fully captured in our moderator coding, potentially obscuring nuanced moderating effects.

Fourth, our findings are limited by the scope of this review. We focused on responses in dyadic interactions, so generalising our partner-effect findings to multi-party scenarios requires caution. We analysed individual-level responses directly related to the interaction, whereas dyad-level responses and those reflecting downstream outcomes were beyond the scope. Moreover, most included studies investigated one-time interactions in controlled lab or online settings; responses in sustained, naturalistic interactions remain unclear. Future work should revisit partner effects using more sophisticated agents capable of long-term interaction. This was partly due to earlier technological constraints, with agents in most included studies relying on traditional machine learning, rule-based algorithms, or Wizard-of-Oz setups. Although our inclusion of “intelligent agent” was technology-agnostic, ranging from traditional technologies to generative AI, none of the studies based on generative AI that we initially identified met our eligibility criteria. Nonetheless, we expect that the growing prevalence of generative AI will spur new comparisons of interactions with generative AI agents vs. humans, enabling re-examination and extension of the current findings. In addition, our review covered only general adult populations and was dominated by WEIRD samples^130^, with limited non-WEIRD representation mainly from East Asia. Future reviews should target younger populations and include broader non-WEIRD populations by synthesising non-English studies.

### Methodological strengths

Despite its limitations, this review demonstrates several methodological strengths. It is a comprehensive meta-analysis comparing a broad range of individuals’ psychological and behavioural responses when interacting with performance-matched agent vs. human partners. We rigorously classified the diverse responses using an empirical-to-conceptual approach^79^. We initially sought to apply a conceptual-to-empirical approach by mapping extracted responses onto an existing taxonomy of psychological and behavioural responses to robots. However, this top-down strategy did not adequately capture the diversity and specificity of responses identified from the included studies. We therefore shifted to an empirical-to-conceptual approach, allowing the classification scheme to emerge from the data. This methodological transition reflects the evolving and interdisciplinary nature of human-agent interaction research. While such agility can introduce uncertainty, particularly in synthesis work, we believe the data-driven approach enabled a more faithful representation of the responses studied and better supported subsequent meta-analytic decisions.

In addition, alongside frequentist analyses, we conducted Bayesian meta-analyses to confirm result consistency. The Bayesian approach also provided complementary insights: Bayes factors quantified the strength of evidence for effect presence/absence, helping distinguish null effects from underpowered results and flag ambiguous cases where pooled effects warrant caution. Sensitivity checks—excluding outliers and influential cases, incorporating approximated effect sizes, and testing alternative Bayesian priors—confirmed the robustness of our main results across analytic decisions. We further tested many potential moderators to explore sources of significant effect-size heterogeneity. Finally, recognising that existing research appraisal tools were less suited to our review, we developed a tailored checklist to assess research quality while capturing its multidimensional nature. The checklist covered quality criteria related to objectives and preregistration, participants, study design, data collection and analysis, results, discussion, as well as ethics and open science. However, certain procedural indicators of research validity (e.g., manipulation checks, inter-rater reliability, and research team consensus procedures) were not incorporated as standalone assessment items. These indicators were not consistently applicable across the included studies or were implemented in varied ways with insufficient reporting detail, limiting their suitability for categorical quality assessment in the current review. Nevertheless, assessment of research validity would likely benefit from considering these indicators, which future reviews may incorporate as reporting practices become more standardised.

### Implications

This review has significant theoretical implications. By synthesising empirical evidence across human-computer interaction, social robotics, psychology, communication, and business studies, we established a cross-disciplinary, systematic understanding of similarities and differences in human responses between human-agent and human-human interactions. Our results confirmed that social responses should be considered as distinct constructs rather than a monolithic entity^61^. Among these social responses, behavioural trust and social alignment showed convergence across interactions, whereas social perceptions, prosociality, and morality diverged. These findings provide partial support for both the Media Equation^49^ and the Threshold Model of Social Influence^51^ and refine their scope by demonstrating that social equivalence varies across response types. In addition, the Threshold Model distinguishes between automatic and deliberate processing underlying human responses^51^, but cognitive processing likely shifts dynamically between these modes during interaction. Although outside the scope of this review, theorising may be expanded to incorporate the temporal nature of human responses.

The review also provides insight into cue-based frameworks, such as the Modality-Agency-Interactivity-Navigability (MAIN) model^55^ and the Media Are Social Actors (MASA) paradigm^75^, by identifying empirical boundary conditions for partner effects in interaction. Specific partner and interaction characteristics moderated partner effects on social perceptions and interaction experiences, while showing limited moderating influence on other response types. Our review suggests that theorising the influence of social cues may benefit from differentiating response types and attending to research paradigms (e.g., interaction-based or evaluative). Moreover, by spanning diverse agent morphologies (e.g., robots, virtual humans, conversational agents), we found that partner effects on most responses, such as trust and social alignment, were consistent regardless of agent embodiment (disembodied vs. embodied) or form (physical vs. virtual). This indicates that trust and social alignment, as core collaborative mechanisms^121,131^, may operate in a morphology-agnostic manner. Prior meta-analyses provide mixed but broadly convergent evidence. Whether robots were embodied or depicted did not significantly moderate the effect of anthropomorphism on attitudinal outcomes, including trust^74^. Embodiment was also not found to moderate cue-related effects on trust, but physical presence did^57^. By contrast, a meta-analysis directly comparing physically co-located and virtually represented robots found no significant difference in trust^132^. Therefore, trust-related responses tend to be relatively robust across agent embodiment and form.

Furthermore, our review benchmarks human responses in human-agent vs. human-human interactions prior to generative AI. Looking ahead, as agents powered by large language models (LLMs) become increasingly integrated into everyday life, sustained and personalised interactions with these agents may attenuate perceived deficits in social attributes. However, this convergence may not extend to prosociality and morality. Reduced prosocial behaviour and moral engagement in human-agent interaction may reflect judgements about moral standing and intrinsic value—issues linked to an entity’s ontological identity^133^. As agents become more humanlike and autonomous, questions surrounding identity could become more, rather than less, salient. For example, research has found that although LLM-generated messages make participants feel emotionally supported, this support is devalued once the source is identified as AI rather than human^134^. We also found that functional behaviours and interaction experiences with agents resemble those with humans. Generative AI may not only maintain this parity but, in certain contexts, shift it. These agents deploy superhuman capabilities while interacting without evoking ego threat or social evaluation concerns^135^, potentially increasing users’ willingness to trust and align with them. A recent study shows that participants can distinguish between LLM- and lawyer-generated legal advice yet still prefer the LLM’s advice^136^, indicating that trust in AI agents may exceed human-human baselines in some domains. Overall, our review provides a benchmark for future research on interaction with generative AI agents, enabling researchers to detect potential shifts in human psychology and behaviour as agent technologies evolve.

Our findings have implications for the development of interactive intelligent agents. First, we found that behavioural trust, social alignment, perceived self-agency, and objective task performance were comparable in human-agent and human-human interactions. That is, when assuming human-equivalent roles with matched performance, agents elicit behavioural trust, facilitate effective interaction, and preserve user agency and performance. This indicates that well-performing agents are afforded instrumental value on par with humans, making them promising collaborators in goal-directed tasks. To move beyond instrumental parity towards successful collaboration, our review also reveals key design considerations. Specifically, we found that agents were attributed less responsibility than humans, whereas collaboration typically assumes shared responsibility^137^, raising concerns that individuals paired with agents may be overburdened with accountability. This necessitates designing robust accountability structures and clear human-agent communication. Another design consideration arises from our review’s focus on agents with human-level performance, which may promote reciprocity and safeguard user agency. However, there are scenarios where leveraging agents’ superhuman capabilities and granting them primary control may be advantageous. Recent AI advances have enabled these agents to surpass humans in both knowledge-based tasks^2,4,5^ and situational judgment tasks^138^. Thus, agent design should strategically calibrate agent capabilities to task demands while ensuring transparency and explainability.

Second, we found reduced prosocial behaviour and moral engagement with agent vs. human partners. Agents were also perceived as less competent, likeable, socially present, and attributed less agency and responsibility. From a moral psychology perspective, our findings reflect reduced moral standing for agents, with weaker attributions of both moral agency (capacity to be a source of moral action) and moral patiency (capacity to be a subject of moral concern and obligations)^139,140^. These deficits suggest that agents are not afforded intrinsic value^140^ to the same extent as humans. While prior research found that participants compensated an ostracised agent during a Cyberball game^141^, mirroring behaviour in human-human ostracism, the compensation effect for agents was smaller than that observed for human targets^142,143^. Accordingly, agents receive some intrinsic value, but to a meaningfully lesser extent. Echoing this interpretation, a meta-analysis of agent anthropomorphism found no significant effect on empathy towards agents^74^, suggesting that apparent human-likeness alone is insufficient to increase empathic concern. These patterns also reveal a distinction between scholarly arguments that artificial agents could warrant moral consideration^133^ and empirical evidence that lay individuals interacting with current agents do not afford them equivalent moral standing and intrinsic value. This warrants caution in morally sensitive domains like healthcare, education, and social services, where decisions directly affect welfare and vulnerability^144,145^, and insufficient prosocial and moral consideration may be consequential. For instance, in AI-mediated tutoring, students may feel less moral obligation to engage honestly or respectfully with the system, potentially increasing dishonest behaviours or disregarding learning guidance. Developers should ensure system benefits outweigh risks of diminished user prosociality and morality and retain human oversight for critical decisions. Design efforts should be put in promoting prosociality/morality in human-agent interaction, potentially leveraging psychological levers like psychological flexibility^146^ and gratitude^147^. Operational practices, including robust accountability frameworks, incident logging^148^, and harm mitigation protocols, should be embedded throughout development cycles.

Whereas agents were generally perceived as possessing fewer social attributes, high heterogeneity in these partner effects highlights important design opportunities. Our review identified moderating effects of several agent and interaction characteristics, indicating that these agent disadvantages are malleable through strategic design and deployment. For example, partner effects on perceived likeability and competence disappeared when appearances were matched (vs. differed). This suggests that calibrating agent appearance relative to human counterparts can improve users’ impressions, which may be particularly relevant when agents serve in customer-facing roles and act as cues that shape corporate brand perceptions^149^. Additionally, the partner effect on agency attribution disappeared in computer-mediated (vs. face-to-face) interactions, suggesting that the interaction medium can be leveraged to shape users’ mental models of agents in line with design intent—whether as intentional actors (using computer-mediated interfaces) or as tools with limited agency (face-to-face to anchor expectations and prevent over-reliance). The partner effect on responsibility attribution disappeared in real-time (vs. hypothetical) interactions. Agent actions, decisions, and role boundaries should therefore be clearly conveyed through interaction to guide appropriate responsibility attribution. High heterogeneity was also observed in partner effects across interaction experiences, though moderators were identified for only two response types. Regarding perceived partner relational qualities, the partner effect was more pronounced in hypothetical (vs. real-time) interactions and when focused on customer orientation (vs. interactivity/rapport/empathy). This highlights the importance of designing agents to convey relational qualities through direct interaction rather than relying on users’ abstract expectations. Moreover, designers should explicitly signal user-oriented intent through agent behaviour during customer interactions, such as by prioritising user goals and demonstrating alignment with user interests. Finally, interaction satisfaction was higher with agents than with humans in oppositional (vs. cooperative) interactions, suggesting opportunities to design agents as assessors, negotiators, or challengers in scenarios where human opponents may trigger discomfort, evaluation anxiety, or interpersonal friction.

## Conclusion

This systematic review and meta-analysis provides a comprehensive understanding of similarities and differences in human responses between human-agent and human-human interactions. Across 23 types of psychological and behavioural responses, we found that individuals exhibited less prosocial behaviour and moral engagement when interacting with agents vs. humans. They also attributed less agency and responsibility to agents, perceiving them as less competent, likeable, and socially present. In contrast, individuals’ social alignment, trust in partners, personal agency, task performance, and interaction experiences were generally comparable when interacting with agents vs. humans. These findings indicate that agents are afforded instrumental value on par with humans yet lack comparable intrinsic value. Furthermore, we identified several moderators across study, participant, partner, interaction, and response characteristics that shaped these partner effects. Overall, this review offers important theoretical insights into human-agent interaction and practical implications for the development of interactive intelligent agents.

## Supplementary information

Transparent Peer Review file

Supplementary information for “A systematic review and meta-analysis of psychological and behavioural responses in human-agent vs. human-human interactions”.

## Data Availability

The dataset supporting the findings of this study, including calculated effect sizes, moderator values, research quality ratings for individual studies, and associated study materials, is available in the Open Science Framework: 10.17605/OSF.IO/4X26R^150^.

## Code availability

All R code used to calculate effect sizes for individual studies and to perform the frequentist and Bayesian meta-analyses is available via the Open Science Framework: 10.17605/OSF.IO/4X26R^151^.
