# Supplementary material for: A systematic review and meta-analysis of psychological and behavioural responses in human-agent vs. human-human interactions
Source: Commun Psychol. 2026 May 5;4:102. doi: 10.1038/s44271-026-00466-z (PMC13351021; doi:10.1038/s44271-026-00466-z)
Supplement: Supplementary file 2 — Supplementary information for “A systematic review and meta-analysis of psychological and behavioural responses in human-agent vs. human-human interactions”. [file 44271_2026_466_MOESM2_ESM.pdf]

Supplementary information for “A systematic review and meta-analysis of psychological and behavioural responses in human-agent vs. human-human interactions”

Jianan Zhou[1*], Fleur Corbett[1], Joori Byun[2], Talya Porat[1], Nejra van Zalk[1]

[1]Dyson School of Design Engineering, Imperial College London, London, United Kingdom
[2]Independent Researcher, London, United Kingdom

[*]Correspondence to: Jianan Zhou
jianan.zhou22@imperial.ac.uk

Contents

Supplementary Table 1. The PRISMA checklist....3
Supplementary Table 2. Search strings....6
Supplementary Table 3. Eligibility criteria....9
Supplementary Table 4. Data extraction and coding table....11
Supplementary Table 5. Research quality assessment tool....13
Supplementary Table 6. Effect size calculation formulae....15
Supplementary Table 7. Characteristics of the eligible studies....19
Supplementary Table 8. Responses with insufficient data for meta-analysis....35
Supplementary Table 9. Univariate meta-regressions and subgroup analyses for different response types....40
Supplementary Table 10. Egger Sandwich tests....46
Supplementary Table 11. Research quality assessment of individual studies....47
Supplementary Table 12. Impact of research quality....51
Supplementary Table 13. Meta-analyses incorporating approximated effect sizes....53
Supplementary Table 14. Outliers and influential cases....57
Supplementary Table 15. Bayesian meta-analyses under different priors....59
Supplementary Table 16. Bayesian meta-analyses for different response types in human-agent vs. human-human interactions....63
Supplementary Table 17. Meta-analytic results for response themes in human-agent vs. human-human interactions....65
Supplementary Fig. 1. Funnel plots....67
Supplementary Note 1. Response classification....70
Supplementary References....71

Supplementary Table 1. The PRISMA checklist

Section and Topic	Item #	Checklist item	Location where item is reported
**TITLE**			
Title	1	Identify the report as a systematic review.	p.1
**ABSTRACT**			
Abstract	2	See the PRISMA 2020 for Abstracts checklist.	p.1
**INTRODUCTION**			
Rationale	3	Describe the rationale for the review in the context of existing knowledge.	p.1-5
Objectives	4	Provide an explicit statement of the objective(s) or question(s) the review addresses.	p.3-4
**METHODS**			
Eligibility criteria	5	Specify the inclusion and exclusion criteria for the review and how studies were grouped for the syntheses.	p.5-6, Supplementary Table 3
Information sources	6	Specify all databases, registers, websites, organisations, reference lists and other sources searched or consulted to identify studies. Specify the date when each source was last searched or consulted.	p.5
Search strategy	7	Present the full search strategies for all databases, registers and websites, including any filters and limits used.	p.5, Supplementary Table 2
Selection process	8	Specify the methods used to decide whether a study met the inclusion criteria of the review, including how many reviewers screened each record and each report retrieved, whether they worked independently, and if applicable, details of automation tools used in the process.	p.6
Data collection process	9	Specify the methods used to collect data from reports, including how many reviewers collected data from each report, whether they worked independently, any processes for obtaining or confirming data from study investigators, and if applicable, details of automation tools used in the process.	p.6-7
Data items	10a	List and define all outcomes for which data were sought. Specify whether all results that were compatible with each outcome domain in each study were sought (e.g. for all measures, time points, analyses), and if not, the methods used to decide which results to collect.	p.6, Table 2, Supplementary Note 1
	10b	List and define all other variables for which data were sought (e.g. participant and intervention characteristics, funding sources). Describe any assumptions made about any missing or unclear information.	p.6, Table 1, Supplementary Table 4
Study risk of bias assessment	11	Specify the methods used to assess risk of bias in the included studies, including details of the tool(s) used, how many reviewers assessed each study and whether they worked independently, and if applicable, details of automation tools used in the process.	p.6-7, Supplementary Table 5
Effect measures	12	Specify for each outcome the effect measure(s) (e.g. risk ratio, mean difference) used in the synthesis or presentation of results.	p.7

Section and Topic	Item #	Checklist item	Location where item is reported
Synthesis methods	13a	Describe the processes used to decide which studies were eligible for each synthesis (e.g. tabulating the study intervention characteristics and comparing against the planned groups for each synthesis (item #5)).	p.5-6
	13b	Describe any methods required to prepare the data for presentation or synthesis, such as handling of missing summary statistics, or data conversions.	p.7, Supplementary Table 6
	13c	Describe any methods used to tabulate or visually display results of individual studies and syntheses.	p.8, p.20 (OSF)
	13d	Describe any methods used to synthesize results and provide a rationale for the choice(s). If meta-analysis was performed, describe the model(s), method(s) to identify the presence and extent of statistical heterogeneity, and software package(s) used.	p.7-9
	13e	Describe any methods used to explore possible causes of heterogeneity among study results (e.g. subgroup analysis, meta-regression).	p.8
	13f	Describe any sensitivity analyses conducted to assess robustness of the synthesized results.	p.7-8
Reporting bias assessment	14	Describe any methods used to assess risk of bias due to missing results in a synthesis (arising from reporting biases).	p.8
Certainty assessment	15	Describe any methods used to assess certainty (or confidence) in the body of evidence for an outcome.	n.a.
**RESULTS**			
Study selection	16a	Describe the results of the search and selection process, from the number of records identified in the search to the number of studies included in the review, ideally using a flow diagram.	p.9, Fig. 2
	16b	Cite studies that might appear to meet the inclusion criteria, but which were excluded, and explain why they were excluded.	n.a. (exclusion reasons noted in Fig. 2)
Study characteristics	17	Cite each included study and present its characteristics.	p.9, Supplementary Table 7
Risk of bias in studies	18	Present assessments of risk of bias for each included study.	p.20 (OSF), Supplementary Table 11
Results of individual studies	19	For all outcomes, present, for each study: (a) summary statistics for each group (where appropriate) and (b) an effect estimate and its precision (e.g. confidence/credible interval), ideally using structured tables or plots.	p.20 (OSF)
Results of syntheses	20a	For each synthesis, briefly summarise the characteristics and risk of bias among contributing studies.	p.10-13, Supplementary Table 12
	20b	Present results of all statistical syntheses conducted. If meta-analysis was done, present for each the summary estimate and its precision	p.10-12,

Section and Topic	Item #	Checklist item	Location where item is reported
		(e.g. confidence/credible interval) and measures of statistical heterogeneity. If comparing groups, describe the direction of the effect.	Table 3, Supplementary Table 16
	20c	Present results of all investigations of possible causes of heterogeneity among study results.	p.12-13, Tables 4-5, Supplementary Table 9
	20d	Present results of all sensitivity analyses conducted to assess the robustness of the synthesized results.	p.14, Supplementary Tables 13-15
Reporting biases	21	Present assessments of risk of bias due to missing results (arising from reporting biases) for each synthesis assessed.	p.13, Supplementary Fig. 1, Supplementary Table 10
Certainty of evidence	22	Present assessments of certainty (or confidence) in the body of evidence for each outcome assessed.	n.a.
**DISCUSSION**			
Discussion	23a	Provide a general interpretation of the results in the context of other evidence.	p.14-16
	23b	Discuss any limitations of the evidence included in the review.	p.16
	23c	Discuss any limitations of the review processes used.	p.16-17
	23d	Discuss implications of the results for practice, policy, and future research.	p.17-20
**OTHER INFORMATION**			
Registration and protocol	24a	Provide registration information for the review, including register name and registration number, or state that the review was not registered.	p.5
	24b	Indicate where the review protocol can be accessed, or state that a protocol was not prepared.	p.5
	24c	Describe and explain any amendments to information provided at registration or in the protocol.	n.a.
Support	25	Describe sources of financial or non-financial support for the review, and the role of the funders or sponsors in the review.	p.29
Competing interests	26	Declare any competing interests of review authors.	p.29
Availability of data, code and other materials	27	Report which of the following are publicly available and where they can be found: template data collection forms; data extracted from included studies; data used for all analyses; analytic code; any other materials used in the review.	p.20

Supplementary Table 2. Search strings

**Scopus: 8,746 records**
(TITLE-ABS-KEY (robot OR chatbot OR chatterbot OR "chat bot" OR "voice user interface" OR "conversational agent" OR "natural language interface" OR "intelligent personal agent" OR "intelligent agent" OR "voice agent" OR "interactive agent" OR "virtual agent" OR "ai agent" OR "embodied agent" OR "intelligent assistant" OR "intelligent personal assistant" OR "voice assistant" OR "virtual assistant" OR "ai assistant" OR "smart speaker" OR "dialog* system") AND TITLE-ABS-KEY ("social actor" OR "social being" OR "media equation" OR "anthropomorphism" OR "social interaction" OR "mindless transfer" OR "human human" OR "human to human" OR "interpersonal" OR "social response" OR "social reaction" OR "social behaviour" OR "social perception" OR "social cognition" OR "social norm" OR "social rule" OR "social script" OR "social facilitation" OR "social categor*" OR "ripple effect" OR "social identity" OR conformity OR reciprocity OR "attribution theory" OR "machine heuristic" OR {human agent} OR {human agents})) AND PUBYEAR > 1999 AND (LIMIT-TO (DOCTYPE , "cp") OR LIMIT-TO (DOCTYPE , "ar")) AND (LIMIT-TO (SRCTYPE , "j") OR LIMIT-TO (SRCTYPE , "p") OR LIMIT-TO (SRCTYPE , "k")) AND (LIMIT-TO (LANGUAGE , "English"))
**Web of Science: 6,438 records**
(TS=(robot OR robots OR chatbot* OR chatterbot* OR "chat bot*" OR "voice user interface*" OR "conversational agent*" OR "natural language interface*" OR "intelligent personal agent*" OR "intelligent agent*" OR "voice agent*" OR "interactive agent*" OR "virtual agent*" OR "ai agent*" OR "embodied agent*" OR "intelligent assistant*" OR "intelligent personal assistant*" OR "voice assistant*" OR "virtual assistant*" OR "ai assistant*" OR "smart speaker*" OR "dialog* system*") AND TS=("social actor*" OR "social being*" OR "media equation" OR "anthropomorphism" OR "social interaction*" OR "mindless transfer" OR "human human" OR "human to human" OR "interpersonal" OR "social response*" OR "social reaction*" OR "social behavio*r*" OR "social perception*" OR "social cognition*" OR "social norm*" OR "social rule*" OR "social script*" OR "social facilitation" OR "social categor*" OR "ripple effect*" OR "social identit*" OR conformity OR reciprocity OR "attribution theor*" OR "machine heuristic*" OR "human agent*")) AND (PY==("2024" OR "2023" OR "2022" OR "2021" OR "2020" OR "2019" OR "2018" OR "2017" OR "2016" OR "2015" OR "2014" OR "2013" OR "2012" OR "2011" OR "2010" OR "2009" OR "2008" OR "2007" OR "2006" OR "2005" OR "2004" OR "2003" OR "2002" OR "2001" OR "2000") AND DT==("PROCEEDINGS PAPER" OR "ARTICLE" OR "EARLY ACCESS") AND LA==("ENGLISH"))
**ACM Digital Library: 2,837 records**
"query": { (Title:(robot OR chatbot OR chatterbot OR "chat bot" OR "chat bots" OR "voice user interface" OR "voice user interfaces" OR "conversational agent" OR "conversational agents" OR "natural language interface" OR "natural language interfaces" OR "intelligent personal agent" OR "intelligent personal agents" OR "intelligent agent" OR "intelligent agents" OR "voice agent" OR "voice agents" OR "interactive agent" OR "interactive agents" OR "virtual agent" OR "virtual agents" OR "ai agent" OR "ai agents" OR "embodied agent" OR "embodied agents" OR "intelligent assistant" OR "intelligent assistants" OR "intelligent personal assistant" OR "intelligent personal assistants" OR "voice assistant" OR "voice assistants" OR "virtual assistant" OR "virtual assistants" OR "ai assistant" OR "ai assistants" OR "smart speaker" OR "smart speakers" OR "dialog system" OR "dialog systems") OR Abstract:(robot OR chatbot OR chatterbot OR "chat bot" OR "chat bots" OR "voice user interface" OR "voice user interfaces" OR "conversational agent" OR "conversational agents" OR "natural language interface" OR "natural language interfaces" OR "intelligent personal

agent" OR "intelligent personal agents" OR "intelligent agent" OR "intelligent agents" OR "voice agent" OR "voice agents" OR "interactive agent" OR "interactive agents" OR "virtual agent" OR "virtual agents" OR "ai agent" OR "ai agents" OR "embodied agent" OR "embodied agents" OR "intelligent assistant" OR "intelligent assistants" OR "intelligent personal assistant" OR "intelligent personal assistants" OR "voice assistant" OR "voice assistants" OR "virtual assistant" OR "virtual assistants" OR "ai assistant" OR "ai assistants" OR "smart speaker" OR "smart speakers" OR "dialog system" OR "dialog systems") OR Keyword:(robot OR chatbot OR chatterbot OR "chat bot" OR "chat bots" OR "voice user interface" OR "voice user interfaces" OR "conversational agent" OR "conversational agents" OR "natural language interface" OR "natural language interfaces" OR "intelligent personal agent" OR "intelligent personal agents" OR "intelligent agent" OR "intelligent agents" OR "voice agent" OR "voice agents" OR "interactive agent" OR "interactive agents" OR "virtual agent" OR "virtual agents" OR "ai agent" OR "ai agents" OR "embodied agent" OR "embodied agents" OR "intelligent assistant" OR "intelligent assistants" OR "intelligent personal assistant" OR "intelligent personal assistants" OR "voice assistant" OR "voice assistants" OR "virtual assistant" OR "virtual assistants" OR "ai assistant" OR "ai assistants" OR "smart speaker" OR "smart speakers" OR "dialog system" OR "dialog systems")) AND (Title:("social actor" OR "social actors" OR "social being" OR "social beings" OR "media equation" OR "anthropomorphism" OR "social interaction" OR "social interactions" OR "mindless transfer" OR "human human" OR "human to human" OR "interpersonal" OR "social response" OR "social responses" OR "social reaction" OR "social reactions" OR "social behaviour" OR "social behaviours" OR "social perception" OR "social perceptions" OR "social cognition" OR "social cognitions" OR "social norm" OR "social norms" OR "social rule" OR "social rules" OR "social script" OR "social scripts" OR "social facilitation" OR "social category" OR "social categories" OR "social categorisation" OR "social categorisations" OR "ripple effect" OR "ripple effects" OR "social identity" OR "social identities" OR conformity OR reciprocity OR "attribution theory" OR "attribution theories" OR "machine heuristic" OR "machine heuristics" OR "human agent" OR "human agents") OR Abstract:("social actor" OR "social actors" OR "social being" OR "social beings" OR "media equation" OR "anthropomorphism" OR "social interaction" OR "social interactions" OR "mindless transfer" OR "human human" OR "human to human" OR "interpersonal" OR "social response" OR "social responses" OR "social reaction" OR "social reactions" OR "social behaviour" OR "social behaviours" OR "social perception" OR "social perceptions" OR "social cognition" OR "social cognitions" OR "social norm" OR "social norms" OR "social rule" OR "social rules" OR "social script" OR "social scripts" OR "social facilitation" OR "social category" OR "social categories" OR "social categorisation" OR "social categorisations" OR "ripple effect" OR "ripple effects" OR "social identity" OR "social identities" OR conformity OR reciprocity OR "attribution theory" OR "attribution theories" OR "machine heuristic" OR "machine heuristics" OR "human agent" OR "human agents") OR Keyword:("social actor" OR "social actors" OR "social being" OR "social beings" OR "media equation" OR "anthropomorphism" OR "social interaction" OR "social interactions" OR "mindless transfer" OR "human human" OR "human to human" OR "interpersonal" OR "social response" OR "social responses" OR "social reaction" OR "social reactions" OR "social behaviour" OR "social behaviours" OR "social perception" OR "social perceptions" OR "social cognition" OR "social cognitions" OR "social norm" OR "social norms" OR "social rule" OR "social rules" OR "social script" OR "social scripts" OR "social facilitation" OR "social category" OR "social categories" OR "social categorisation" OR "social categorisations" OR "ripple effect" OR "ripple effects" OR "social identity" OR "social identities" OR conformity OR reciprocity OR "attribution theory" OR "attribution theories" OR "machine heuristic" OR "machine heuristics" OR "human agent" OR "human agents")) }

"filter": { E-Publication Date: (01/01/2000 TO *), ACM Content: DL }
**Ovid PsycInfo: 1,125 records**
1. ((robot or robots or chatbot* or chatterbot* or "chat bot*" or "voice user interface*" or "conversational agent*" or "natural language interface*" or "intelligent personal agent*" or "intelligent agent*" or "voice agent*" or "interactive agent*" or "virtual agent*" or "ai agent*" or "embodied agent*" or "intelligent assistant*" or "intelligent personal assistant*" or "voice assistant*" or "virtual assistant*" or "ai assistant*" or "smart speaker*" or "dialog* system*") and ("social actor*" or "social being*" or "media equation" or "anthropomorphism" or "social interaction*" or "mindless transfer" or "human human" or "human to human" or "interpersonal" or "social response*" or "social reaction*" or "social behavio*r*" or "social perception*" or "social cognition*" or "social norm*" or "social rule*" or "social script*" or "social facilitation" or "social categor*" or "ripple effect*" or "social identit*" or conformity or reciprocity or "attribution theor*" or "machine heuristic*" or "human agent*")).tw.<br>2. limit 1 to ((peer reviewed journal or conference proceedings) and english language and yr="2000 -current")

Supplementary Table 3. Eligibility criteria

**Publication characteristics**		
**Type**	**Inclusion criteria**	**Exclusion criteria**
Publication date	• Articles published in 2000 and beyond.	• Articles published before 2000.
Type of publication	• Articles published in peer-reviewed journals, conferences, or workshops.<br>• Articles that included empirical research using quantitative or mixed methods (extracting only quantitative data).	• Theoretical papers, study protocols, reviews, descriptive and qualitative studies.<br>• Articles in trade journals, book chapters, panel reports, technical notes, editorials, talks, and semi-finished works (i.e., abstracts, posters, demos, and work in progress).<br>• Articles for which the full text was not retrievable and could not be obtained from the authors upon request.<br>• Articles that presented substantially overlapping research with another publication (already included in the review corpus).
Language of publication	• Articles written in English.	• Articles written in a language other than English.
**Study characteristics (PECO)**		
Population: Participants	• Studies with a research sample comprising healthy adults aged ≥18 years old.	• Studies that involved children and teenagers as participants.<br>• Studies that involved populations with specific health conditions.
Exposure: Human-agent interaction (HAI)	• Studies that researched physical (smart speakers and robots) or virtual agents (chatbots, artificial agents, and virtual humans), regardless of technique implementation.<br>• Studies that investigated HAI through direct engagement in (perceived) real-time or hypothetical dyadic scenarios. Real-time scenarios include both true real-time interactions and those simulated as real-time (e.g., partner responses strategically presented to be perceived by participants as live).<br>• Studies that investigated HAI where the agent identity of the interaction partner was known or	• Studies that researched telepresence robots, avatars representing humans, or systems assisting HHI but not as active interactants.<br>• Studies that researched smartphones, desktop/laptop computers, drones, or autonomous vehicles.<br>• Studies that investigate HAI from a third-person observational perspective (i.e., without participants directly engaging in the interaction).<br>• Studies that investigated multi-party HAI involving more than two interactants.<br>• Studies where participants were unclear about the partner's identity during the interaction.

	disclosed to participants upfront, regardless of whether this disclosure involved deception.	
Comparison: Human-human interaction (HHI)	• Studies that investigated human responses in both HAI and HHI and made direct comparisons.<br>• Studies where participants received similar treatment in HAI and HHI; that is, expect for possible voice and appearance differences, the interaction task, dynamics, and partner role and behaviour were consistent across both conditions.	• Studies that only had indirect HAI-HHI comparisons, investigating human responses in HAI with a comparison of their findings to results from previous, parallel HHI research, or an exploration of phenomena common in HHI.<br>• Studies that lacked comparisons, such as those investigating the effects of specific agent features by comparing participants' responses to agents with and without these features, and those applying HHI findings to agent design without user studies or, if user studies were conducted, without comparisons to responses in HHI.
Outcome: Human responses	• Studies that examined participants' individual-level psychological or behavioural responses during or immediately after the interaction.	• Studies that focused on dyad-level responses.<br>• Studies that focused on comparative responses (e.g., choosing between agent and human partners) without examining specific responses in both HAI and HHI.<br>• Studies that focused on physiological responses.<br>• Studies that focused on responses reflecting broader outcomes or implications beyond the direct interaction context.

Supplementary Table 4. Data extraction and coding table

Variables [information]	Extraction and coding
**Response characteristics**	
[response]	specific human responses measured
[response measure]	measure of each response
[memos]	analytic memos for post-hoc response classification
[statistics for ES calculation]	sample size, group sizes, *M*s, *SD*s, *t*-value, *p*-value, *F*-value ($df = 1$), 2×2 frequency table or proportions, reported effect size, and other statistics
res_time	response measured after the interaction = 0<br>response measured during the interaction = 1
res_domain	psychological = 0<br>behavioural = 1
reported_result	$DV_{HAI} > DV_{HHI}$ (sig) = 1<br>$DV_{HAI} = DV_{HHI}$ (non-sig) = 0<br>$DV_{HAI} < DV_{HHI}$ (sig) = -1
**Study characteristics**	
[study aim]	research aims of the study
[study conclusion]	main conclusions of the study
[outlet name]	the name of journal or conference where the study was published
pub_type	journal paper = 0<br>conference paper = 1
pub_year	publication year
study_setting	lab experiment = 0<br>online experiment = 1<br>field experiment = 2
study_design	between-subjects = 0<br>within-subjects = 1
**Participant characteristics**	
[compensation]	participant compensation method
[other demographics]	other participant demographics reported
age	the mean age of participants
per_female	the percentage of female participants
sample_continent	Africa = 0<br>Asia = 1<br>Europe = 2<br>North America = 3<br>Oceania = 4<br>South America = 5
sample_WEIRD	WEIRD = 0<br>non-WERID = 1
**Partner characteristics**	
[human partner image]	the image of the human partner shown in the study
[agent partner image]	the image of the agent partner shown in the study
human_type	research team member = 0<br>participant = 1<br>pseudo-human = 2

Variables [information]	Extraction and coding
	vignette-described partner = 3
agent_operation	autonomous = 0<br>Wizard-of-Oz = 1<br>vignetted-described = 2
agent_form	physical = 0<br>virtual = 1
agent_embod	disembodied = 0<br>embodied = 1
robot_appearance	non-humanoid (ABOT score 0 - 10) = 0<br>semi-humanoid (10 - 40) = 1<br>humanoid (40 - 70) = 2<br>android (70 - 100) = 3
appearance_diff	appearance matched = 0<br>appearance differed = 1
voice_diff	voice matched = 0<br>voice differed = 1
**Interaction characteristics**	
[interaction task]	the specific interaction task
[interaction task image]	the image of the interaction task shown in the study
[memos]	analytic memos for post-hoc task classification
[interactant role]	the participant's and partner's roles in the interaction task
int_power	symmetrical = 0<br>asymmetrical = 1
int_realism	real-time = 0<br>hypothetical = 1
int_flow	bidirectional = 0<br>unidirectional = 1
int_medium	computer-mediated = 0<br>face-to-face = 1<br>VR-mediated = 2
int_structure	non-structured = 0<br>semi-structured = 1<br>structured = 2
int_nature	neutral = 0<br>cooperative = 1<br>oppositional (competitive/conflictual) = 2<br>mixed = 3

Supplementary Table 5. Research quality assessment tool

Quality criteria	Assessment items
**Study design rigour**	
Objectives and preregistration	1. Objectives/questions/hypotheses sufficiently described?<br>2. Study pre-registered or protocol published?
Participants	3. Sample size established by a-priori power analysis?<br>4. Sample size appropriate for HAI-HHI comparison?<br>**YES:** A-priori or post-hoc power analysis was reported; or the sample size met Brysbaert's estimates[1]: 200 (100 per group) for between-subjects designs and 52 for within-subjects designs.<br>**PARTIAL**: There was insufficient information to evaluate the sample size, as no power analysis was reported. But the sample size appeared appropriate with some statistically significant results; or it met the rule of thumb (i.e., at least 20 in each group or cell of a study design).<br>**NO**: No statistically significant results were reported, and the sample size did not meet the rule of thumb.<br>5. Subject characteristics described (age and sex at minimum)?<br>6. Sampling and recruitment strategies described?<br>**YES:** Both sampling and recruitment strategies were described. Sampling strategies involved specifying the type of sampling used (e.g., representative, convenience, or snowball sampling) or describing the sample selection criteria.<br>**PARTIAL**: Only sampling or recruitment strategies were described.<br>**NO:** No strategies were described.
Study design	7. Study design evident and reasonable?<br>8. Randomisation used for participant assignment to HAI and HHI conditions?<br>**YES**: Randomisation (individually randomised parallel-group trials for between-subjects designs or randomised cross-over trials for within-subjects designs) was reported, with a specific randomisation method described.<br>**PARTIAL**: Randomisation was reported, but the method was not described; or quasi-randomisation (e.g., alternation, birth date, date of presentation, and alphabetical order) was reported.<br>**NO**: No randomisation was reported.<br>9. Participants blinded to the study's objectives/questions/hypotheses?<br>**YES**: It was explicitly stated that participants were unaware of the research purpose or were informed about a fake purpose.<br>**PARTIAL**: The study used a method implying participant blinding, like deception regarding the interaction partner's identity.<br>**NO**: There was no information related to participant blinding.<br>10. Controlled for biases and confounding, other than ensuring consistency between HAI and HHI conditions?
**Data & reporting rigour**	
Data collection and analysis	11. Data collection tools shown to be valid?<br>12. Data collection tools shown to be reliable?<br>**YES:** The data collection tools were shown to be reliable via consistent use in previous studies; for self-report scales, reliability ratings were documented.<br>**PARTIAL:** Outcome variables were measured using self-report scales from pre-existing literature, but reliability ratings were not reported; or measurements use self-built scales without reliability testing, but the specific items appeared conceptually sound and relevant to the construct being measured.<br>**NO:** Data collection tools were self-built without reliability testing, and the specific items were conceptually irrelevant or poorly defined.<br>13. Data analytic methods described and appropriate?
Results	14. Results reported accurately and in sufficient detail?<br>**YES:** Testing of each hypothesis was reported; or for each main outcome variable, unless there were specific hypotheses, all statistical effects were reported (e.g., reporting both the main effects and the interaction for a 2×2 ANOVA).<br>**PARTIAL:** Statistical effects were not reported in detail (e.g., reporting only interaction

Quality criteria	Assessment items
	effects); or there were some statistical errors.<br>**NO:** There were substantial statistical errors that compromised the study findings.<br>15. Estimates of variance for the main results or outcome variables provided?<br>***Note:*** The direct estimate of variance is sample variance (s^2); other measures derived from the variance estimate, such as standard deviation (*SD*), standard error of the mean (*SE*), and confidence interval, are also acceptable.<br>16. Effect sizes for the main results provided?<br>***Note:*** Both standardised and unstandardised effect sizes are acceptable.
**Broad research integrity**	
Discussion	17. Limitations, future research suggestions, and discussions on result generalisability provided?<br>18. Conclusions adequately supported by the results?
Ethics and open science	19. Ethics approval explicitly reported as obtained?<br>20. Participant informed consent explicitly reported as obtained?<br>21. Potential conflicts of interest explicitly reported?<br>22. Data publicly available?<br>23. Article available in open access?

Note. The items within the "study design rigour" and "data & reporting rigour" clusters are study-level criteria that directly relate to the risk of bias. The items within the "broad research integrity" cluster are article-level criteria that extend beyond the assessment of a study's risk of bias, contributing to the overall research quality. Each item was assessed on a 3-point scale: Yes (2), Partial (1), or No (0). HAI refers to human-agent interaction, and HHI refers to human-human interaction.

Supplementary Table 6. Effect size calculation formulae

1 HAI-HHI between-subjects comparison

1.1 Calculation using means, standard deviations, and group sizes

We calculated most of the effect sizes using this approach[2,3]. When these statistics in studies were presented only in figures, we used extracted them via a pixel-based approach[4] using *WebPlotDigitizer v5*[5]. Please note that statistics with subscript 1 refer to those in the HAI condition, and statistics with subscript 2 refer to those in the HHI condition.

$$d = \frac{M_1 - M_2}{\sqrt{\frac{(n_1 - 1) \times SD_1^2 + (n_2 - 1) \times SD_2^2}{n_1 + n_2 - 2}}}$$

$$V_d = \frac{n_1 + n_2}{n_1 \times n_2} + \frac{d^2}{2(n_1 + n_2)}$$

If only the total sample size was known, we employed the equal group size assumption[6,7].

$$d = \frac{M_1 - M_2}{\sqrt{\frac{SD_1^2 + SD_2^2}{2}}}$$

We then calculated Hedges' g, along with its variance, standard error (SE) and 95% confidence interval (CI) using these formulae[2].

$$g = J \times d \qquad J = 1 - \frac{3}{4(N-2) - 1}$$

$$V_g = J^2 \times V_d \qquad SE_g = \sqrt{V_g}$$

$$CI_g = [g_L = g - 1.96 \times SE_g, \quad g_U = g + 1.96 \times SE_g]$$

Combine subgroups: When *M*s and *SD*s were reported for subgroups, we combined them to calculate these statistics for the full group[2,8]. For example, when the subgroup statistics were presented in 2×2 tables, we collapsed cells under the HAI and HHI conditions, respectively, to derive the combined statistics. Below are the formulae for combining subgroups in the HAI condition.

$$n_1 = n_{11} + n_{12}$$

$$M_1 = \frac{n_{11} \times M_{11} + n_{12} \times M_{12}}{n_{11} + n_{12}}$$

$$SD_1 = \sqrt{\frac{(n_{11} - 1) \times SD_{11}^2 + (n_{12} - 1) \times SD_{12}^2 + \frac{n_{11} \times n_{12}}{n_{11} + n_{12}} \times (M_{11} - M_{12})^2}{n_{11} + n_{12} - 1}}$$

1.2 Calculation using the *t*-value and group sizes

The *t*-value could be converted from (1) the *p*-value in a one-way between-subjects ANOVA or independent sample *t*-test, or from (2) a one degree of freedom *F*-value (provided no significant interaction with another factor)[4,9].

$$d = t \times \sqrt{\frac{1}{n_1} + \frac{1}{n_2}}$$

$$V_d = \frac{n_1 + n_2}{n_1 \times n_2} + \frac{d^2}{2(n_1 + n_2)}$$

If only the total sample size was known, we employed the equal group size assumption[6,7].

$$d = 2 \times \frac{t}{\sqrt{N}}$$

We calculated Hedges' g, along with its variance, SE and 95% CI using the formulae[2] above.

- Some studies only reported threshold values for required statistics. we thus estimated effect sizes from the thresholds (e.g., $p = 0.005$ for $p < 0.005$; $t = 1$ for $t < 1$) and included these approximations in sensitivity analyses.

1.3 Calculation using the 2×2 frequency table

We calculated effect sizes for binary dependent variables using this approach[10]. Please note that *a*, *b*, *c*, and *d* are the cell frequencies of a 2×2 frequency table. If proportions and group sizes were reported, we used them to re-create the 2×2 frequency table.

$$d = log\left(\frac{a \times d}{b \times c}\right) \times \frac{\sqrt{3}}{\pi}$$

$$V_d = \left(\frac{1}{a} + \frac{1}{b} + \frac{1}{c} + \frac{1}{d}\right) \times \frac{3}{\pi^2}$$

We calculated Hedges' *g*, along with its variance, *SE* and 95% CI using the formulae[2] above.

1.4 Calculation using the reported effect size and group sizes

We used the reported effect sizes (e.g., *d* and *r*), only when statistics for the aforementioned approaches were unavailable[11]. We decided to do so for consistency, since most of the included studies did not report any effect size information.

$$d = \frac{2 \times r}{\sqrt{1 - r^2}}$$

We calculated Hedges' *g*, along with its variance, *SE* and 95% CI using the formulae[2] above.

1.5 Calculation using other statistics

Effect sizes calculated using the following approaches were statistically adjusted estimates. They were derived from multivariable models that included covariates or other predictors, and thus reflected the unique contribution of HAI vs. HHI after partialling out variance shared with other variables in the model. We only included these effect sizes in sensitivity analyses.

- Some studies using multifactor ANOVAs did not provided the required statistics, but reported the ***F*-value, *df*s, and/or partial *η*²** for the main effect of HAI vs. HHI alongside significant interactions with other factors (if no significant interaction was present, the F-value was first converted to t-value and then to a proper effect size; see 1.2).
  We calculated the effect size from these reported statistics using this approach[12,13].

$$\eta_p^2 = \frac{F \times df_{effect}}{F \times df_{effect} + df_{error}} \qquad df_{effect} = 1$$

$$d = 2 \times \sqrt{\frac{\eta_p^2}{1 - \eta_p^2}}$$

$$V_d = \frac{n_1 + n_2}{n_1 \times n_2} + \frac{d^2}{2(n_1 + n_2)}$$

  We calculated Hedges' *g*, along with its variance, *SE* and 95% CI using the formulae[2] above.

- Some studies using multivariable OLS models did not provided the required statistics, but reported the ***SD* of the outcome variable, unstandardised coefficient with its *SE*, and group sizes**.
  We calculated the effect size from these reported statistics using this approach[10].

$$d = \frac{b}{s_{pooled}} \qquad s_{pooled} = \sqrt{\frac{SD_y^2 \times (n_1 + n_2 - 1) - \frac{b^2 \times (n_1 \times n_2)}{n_1 + n_2}}{n_1 + n_2 - 2}}$$

$$V_d = \left(\frac{d \times SE_b}{b}\right)^2$$

  We calculated Hedges' *g*, along with its variance, *SE* and 95% CI using the formulae[2] above.

- Some studies using multivariable logistic models did not provided the required statistics, but reported the **odds ratio and the *SE* of the unstandardised logit coefficient**.
  The odds ratio could be converted from the unstandardised logit coefficient.
  We calculated the effect size from these reported statistics using this approach[2].

$$d = \log OR \times \frac{3}{\pi}$$

$$V_d = (SE_b)^2 \times \frac{3}{\pi^2}$$

We calculated Hedges' g, along with its variance, *SE* and 95% CI using the formulae[2] above.

2 HAI-HHI within-subjects comparison

2.1 Calculation using means, standard deviations, and sample size

Please note that statistics with subscript 1 refer to those in the HAI condition, and statistics with subscript 2 refer to those in the HHI condition.

$$d = \frac{M_1 - M_2}{\sqrt{SD_1^2 + SD_2^2 - 2 \times r \times SD_1 \times SD_2}} \times \sqrt{2 \times (1 - r)}$$

$$V_d = \left(\frac{1}{N} + \frac{d^2}{2 \times N}\right) \times 2 \times (1 - r)$$

Correlations between specific responses in the HAI and HHI conditions were typically not reported; we imputed $r = 0.50$ drawing on previous meta-analytical reports[4,9,11].

We calculated Hedges' g, along with its variance, *SE* and 95% CI using these formulae[2].

$$g = J \times d \qquad J = 1 - \frac{3}{4(N - 1) - 1}$$

$$V_g = J^2 \times V_d \qquad SE_g = \sqrt{V_g}$$

$$CI_g = \left[g_L = g - 1.96 \times SE_g, \quad g_U = g + 1.96 \times SE_g\right]$$

2.2 Calculation using the *t*-value and sample size

The *t*-value could be converted from (1) the *p*-value in a one-way within-subjects ANOVA or paired sample *t*-test, or from (2) a one degree of freedom *F*-value (provided no significant interaction with another factor)[4,9].

$$d = t \times \sqrt{\frac{2 \times (1 - r)}{N}}$$

$$V_d = \left(\frac{1}{N} + \frac{d^2}{2 \times N}\right) \times 2 \times (1 - r)$$

We calculated Hedges' g, along with its variance, *SE* and 95% CI using the formulae[2] above.

- Some studies only reported threshold values for required statistics. we thus estimated effect sizes from the thresholds (e.g., $p = 0.005$ for $p < 0.005$; $t = 1$ for $t < 1$) and included these approximations in sensitivity analyses.

2.3 Calculation using the reported effect size and sample size

We used the reported Cohen's *d*, only when statistics for the aforementioned approaches were unavailable[11]. We decided to do so for consistency, since most of the included studies did not report any effect size information. We calculated Hedges' g, along with its variance, *SE* and 95% CI using the formulae[2] above.

2.4 Calculation using other statistics

Effect sizes calculated in the following approach were included in sensitivity analyses.

- Some studies using multifactor ANOVAs did not provided the required statistics, but reported the ***F*-value, *df*s, and/or partial η^2** for the main effect of HAI vs. HHI alongside significant interactions with other factors (if no significant interaction was present, the *F*-value was first converted to *t*-value and then to a proper effect size; see 2.2).
  We calculated the effect size from these reported statistics using this approach[12,13].

$$\eta_p^2 = \frac{F \times df_{effect}}{F \times df_{effect} + df_{error}} \qquad df_{effect} = 1$$

$$d = \sqrt{\frac{\eta_p^2}{1 - \eta_p^2}}$$

$$V_d = \left(\frac{1}{N} + \frac{d^2}{2 \times N}\right) \times 2 \times (1 - r)$$

We calculated Hedges' g, along with its variance, *SE* and 95% CI using the formulae[2] above.

Effect size aggregation

Effect sizes were aggregated when appropriate[2]; for example, when studies analysed each item of a multi-item scale assessing a specific human response, we aggregated the resulting item-level effect sizes.

$$g_{agg} = \frac{1}{m} \times \left(\sum_{j=1}^{m} g_j\right)$$

$$V_{agg} = \left(\frac{1}{m}\right)^2 \times var\left(\sum_{j=1}^{m} g_j\right) = \left(\frac{1}{m}\right)^2 \times \left[\sum_{j=1}^{m} V_j + \sum_{j \neq k}(r_{jk}\sqrt{V_j V_k})\right]$$

Here, r_{jk} denoted the correlation between outcomes Y_i and Y_j. In absence of the value of this correlation, we imputed it as 0.50 drawing on previous meta-analytical reports[14,15].

Imputation for missing non-significant effects

- Some studies did not provide the required statistics, but reported **non-significant effects**. Drawing on previous meta-analytical reports[16,17], these missing effect sizes were imputed as *zero* (zero-coded) or as the maximum non-significant value (max-coded; $p = 0.05$) applied in both possible directions—max^{+} (positive) and max^{-} (negative). We also included these imputed estimates in sensitivity analyses, assuming the true effect sizes lie between these bounds.

Note. Eligible studies had both between- and within-subjects HAI-HHI comparisons. To achieve comparable effect sizes across the literature, studies with within-subjects comparisons accounted for correlations between responses in the HAI and HHI conditions that could otherwise inflate effect sizes. Accordingly, we used the formulae for d_s in studies with between-subjects comparison, which represents the standardised mean difference between two independent groups in the sample[3]; for studies with within-subjects comparisons, we applied the formulae for d_{rm}, which is comparable to d_s for repeated measures[3,18]. HAI refers to human-agent interaction, and HHI refers to human-human interaction.

Supplementary Table 7. Characteristics of the eligible studies

No	Study	HAI vs. HHI	Sample size	Age, M (SD)	% Female	Agent partner	Human partner	Interaction task	ESs calculated	ESs in meta-analysis
1	(Abril, 2023)[19] S1	B	33<br>HAI = 20<br>HHI = 13	27.25 (8.94)	66.00%	virtual human: virtually embodied	pseudo-human	document translation & production task	2	0
2	(Alarcon, 2023)[20] S1	B	131<br>HAI = NR<br>HHI = NR	25.93 (9.79)	34.00%	NAO robot: physically embodied	research team member	Checkmate game	2	2
3	(Appel, 2012)[21] S1	B	90<br>HAI1 = NR<br>HAI2 = NR<br>HHI = NR	36.26 (12.26)	54.44%	conversational agent: virtually disembodied<br>virtual human: virtually embodied	research team member	self-story telling	16	11
4	(Babel, 2021)[22] S1	B	61<br>HAI1 = NR<br>HAI2 = NR<br>HAI3 = NR<br>HHI = NR	24.00 (8.00)	77.00%	CR700/Roomba/Pepper robot: physically embodied	vignette-described partner	goal conflict simulation (the train station scenario)	9	0
5	(Babel, 2021)[22] S2	B	93<br>HAI1 = NR<br>HAI2 = NR<br>HAI3 = NR<br>HHI = NR	38.00 (17.00)	53.00%	Pepper/TIAGo/iRobot robot: physically embodied	vignette-described partner	goal conflict simulation (the kitchen scenario)	9	0
6	(Bah, 2022)[23] S1	W	11<br>HAI = 11<br>HHI = 11	NR	27.27%	Reachy robot: physically embodied	NR	cube hand-over task	5	0
7	(Bailenson, 2003)[24] S1	B	80<br>HAI = 40<br>HHI = 40	19.61 (1.92)	50.00%	virtual human: virtually embodied	pseudo-human	approach and label memorization task	2	2
8	(Bailenson, 2003)[24] S2	B	78<br>HAI = NR<br>HHI = NR	NR	50.00%	virtual human: virtually embodied	pseudo-human	personal space violation task	1	1
9	(Banks, 2020)[25] S1	B	39<br>HAI = 21<br>HHI = 18	31.90 (10.34)	19.50%	conversational agent: virtually disembodied	pseudo-human	cyber-sex conversation	8	7

No	Study	HAI vs. HHI	Sample size	Age, M (SD)	% Female	Agent partner	Human partner	Interaction task	ESs calculated	ESs in meta-analysis
10	(Bartneck, 2008)[26] S1	W	12<br>HAI1 = 12<br>HAI2 = 12<br>HAI3 = 12<br>HHI = 12	29.90	50.00%	PKD/Tron-X/AIBO robot:<br>physically embodied	pseudo-human	naming or counting object task (collaboration task)	48	0
11	(Bartneck, 2008)[26] S2	W	25<br>HAI = 25<br>HHI = 25	24.90	24.00%	AIBO robot:<br>physically embodied	pseudo-human	naming or counting object task (collaboration task)	12	0
12	(Belanche, 2020)[27] S1	B	331<br>HAI = NR<br>HHI = NR	NR	71.60%	Pepper robot:<br>physically embodied	vignette-described partner	hotel reception service simulation (with service failure and success scenarios)	1	1
13	(Belanche, 2020)[27] S2	B	229<br>HAI = 153<br>HHI = 76	NR	44.50%	HZX robot:<br>physically embodied	vignette-described partner	restaurant service failure simulation	1	1
14	(Bergmann, 2015)[28] S1	B	45<br>HAI = 23<br>HHI = 22	27.90<br>(6.68)	52.22%	virtual human:<br>virtually embodied	pseudo-human	picture naming and matching game (alignment task)	1	1
15	(Bergmann, 2015)[28] S2	B	54<br>HAI = 27<br>HHI = 27	26.41<br>(7.21)	59.26%	virtual human:<br>virtually embodied	research team member	tangram description and matching game (alignment task)	1	1
16	(Blankenda al, 2015)[29] S1	B	28<br>HAI = 14<br>HHI = 14	NR	46.43%	virtual human:<br>virtually embodied	research team member	story listening	1	0
17	(Bouquet, 2024)[30] S2	B	101<br>HAI = 69<br>HHI = 32	19.02<br>(1.65)	93.52%	Meccanoid robot:<br>physically embodied	research team member	joint go/no-go Simon task	1	1
18	(Bowman, 2019)[31] S1	B	70<br>HAI = 35<br>HHI = 35	20.52<br>(3.75)	50.50%	Jibo robot:<br>physically embodied	research team member	Castle Crashers game	7	6
19	(Bunlon, 2018)[32] S2	W	67<br>HAI = 67<br>HHI = 67	19.80<br>(1.48)	75.00%	robot hand:<br>physically embodied	pseudo-human	joint go/no-go Simon task	1	1
20	(Čaić, 2020)[33] S1	B	58<br>HAI = 36<br>HHI = 22	78.79<br>(9.85)	72.41%	Vizzy robot:<br>physically embodied	research team member	exergame assisted by an agent/human coach	4	3

No	Study	HAI vs. HHI	Sample size	Age, M (SD)	% Female	Agent partner	Human partner	Interaction task	ESs calculated	ESs in meta-analysis
21	(Caruana, 2019)[34] S1	W	20<br>HAI = 20<br>HHI = 20	24.70 (9.05)	75.00%	virtual human: virtually embodied	pseudo-human	joint attention task	8	4
22	(Chen, 2023)[35] S1	W	18<br>HAI = 18<br>HHI = 18	20.56 (2.22)	66.67%	Erica robot: physically embodied	pseudo-human	dictator game	1	1
23	(Chen, 2023)[35] S2	W	74<br>HAI = 74<br>HHI = 74	23.23 (1.28)	45.95%	Erica robot: physically embodied	pseudo-human	dictator game	1	1
24	(Chen, 2023)[35] S3	B	128<br>HAI = 64<br>HHI = 64	20.66 (1.86)	60.94%	Erica robot: physically embodied	pseudo-human	dictator game	2	1
25	(Choi, 2019)[36] S1	B	173<br>HAI = NR<br>HHI = NR<br>other = NR	35.55	41.60%	NAO robot: physically embodied	vignette-described partner	concierge restaurant recommendation simulation	2	1
26	(Cominelli, 2021)[37] S1	B	98<br>HAI = 50<br>HHI = 48	26.00	55.56%	FACE robot: physically embodied	research team member	economic trust game	1	1
27	(Cowan, 2015)[38] S1	B	42<br>HAI1 = 14<br>HAI2 = 14<br>HHI = 14	23.34 (4.19)	54.76%	conversational agent: virtually disembodied	research team member	picture naming and matching game (alignment task)	2	2
28	(Cowan, 2015)[38] S2	B	57<br>HAI1 = 19<br>HAI2 = 19<br>HHI = 19	21.30 (3.76)	52.63%	conversational agent: virtually disembodied	research team member	picture naming and matching game (alignment task)	2	2
29	(De Carolis, 2014)[39] S1	B	30<br>HAI = 15<br>HHI = 15	NR	NR	virtual human: virtually embodied	research team member	nutrition and training programme recommendation	2	0
30	(De Kleijn, 2019)[40] S1	B	90<br>HAI1 = 32<br>HAI2 = 30<br>HHI = 28	22.40 (3.22)	100.00%	Q.bo/hexapod robot: physically embodied	pseudo-human	dictator game, ultimatum game	4	4
31	(De Melo,	B	477	NR	32.60%	virtual human:	pseudo-human	iterated prisoner's dilemma	1	1

No	Study	HAI vs. HHI	Sample size	Age, M (SD)	% Female	Agent partner	Human partner	Interaction task	ESs calculated	ESs in meta-analysis
	2019)[41] S1a		HAI = 228<br>HHI = 249			virtually embodied		(recasting as an investment game)		
32	(De Melo, 2019)[41] S1b	B	468<br>HAI = 240<br>HHI = 228	NR	36.80%	virtual human: virtually embodied	pseudo-human	iterated prisoner's dilemma (recasting as an investment game)	1	1
33	(De Visser, 2016)[42] S1	W	20<br>HAI = 20<br>HHI = 20	22.00 (1.40)	40.00%	virtual human: virtually embodied	pseudo-human	TNO Trust Task: a pattern detection task including trust formation and violation	6	5
34	(De Visser, 2016)[42] S2	W	17<br>HAI = 17<br>HHI = 17	20.40 (2.43)	47.00%	virtual human: virtually embodied	pseudo-human	TNO Trust Task: a pattern detection task including trust formation and violation	6	5
35	(De Visser, 2016)[42] S3	W	20<br>HAI = 20<br>HHI = 20	20.70 (2.20)	50.00%	virtual human: virtually embodied	pseudo-human	TNO Trust Task: a pattern detection task including trust formation, violation and repair	6	5
36	(Desideri, 2019)[43] S1	W	29<br>HAI = 29<br>HHI = 29	24.61 (2.42)	58.62%	NAO robot: physically embodied	research team member	cognitive assessment task	8	6
37	(Edwards, 2019)[44] S1	B	69<br>HAI = NR<br>HHI = NR	20.43 (1.96)	52.20%	Pepper robot: physically embodied	research team member	"get-to-know you" self-disclosure task	3	2
38	(Edwards, 2021)[45] S1	B	58<br>HAI = 30<br>HHI = 28	21.65 (4.84)	63.80%	Pepper robot: physically embodied	research team member	public speaking task	5	4
39	(Frank, 2023)[46] S1	B	488<br>HAI = 245<br>HHI = 243	35.82 (11.66)	48.00%	Pepper robot: physically embodied	vignette-described partner	everyday shopping simulation	2	2
40	(Frank, 2023)[46] S2	B	352<br>HAI = 173<br>HHI = 179	36.95 (11.80)	50.60%	Pepper robot: physically embodied	vignette-described partner	everyday shopping simulation	2	2
41	(Frank, 2023)[46] S3	B	945<br>HAI = 472<br>HHI = 473	36.80 (12.00)	51.10%	Pepper robot: physically embodied	vignette-described partner	embarrassing shopping simulation	2	2
42	(Garvey, 2023)[47] S1a	B	174<br>HAI = NR<br>HHI = NR	21.20	56.00%	bot: virtually disembodied	vignette-described partner	product resale simulation (with offer expected and worse-than-expected	1	0

No	Study	HAI vs. HHI	Sample size	Age, M (SD)	% Female	Agent partner	Human partner	Interaction task	ESs calculated	ESs in meta-analysis
								scenarios)		
43	(Garvey, 2023)[47] S1b	B	299<br>HAI = NR<br>HHI = NR	41.90	53.00%	bot:<br>virtually disembodied	vignette-described partner	product resale simulation (with offer expected and better-than-expected scenarios)	1	0
44	(Garvey, 2023)[47] S2	B	698<br>HAI = NR<br>HHI = NR	21.20	59.00%	no-name robot:<br>physically embodied	vignette-described partner	Uber service simulation (with offer expected, worse-than-expected, and better-than-expected scenarios)	7	4
45	(Garvey, 2023)[47] S3a	B	403<br>HAI = NR<br>HHI = NR	34.90	64.00%	bot:<br>virtually disembodied	vignette-described partner	imagined ultimatum game (with offer expected and worse-than-expected scenarios)	1	0
46	(Giroux, 2022)[48] S1	B	128<br>HAI = NR<br>HHI = NR	40.20 (13.51)	43.80%	bot:<br>virtually disembodied	vignette-described partner	moral decision-making in service encounter simulation	2	2
47	(Giroux, 2022)[48] S2	B	303<br>HAI = NR<br>HHI = NR<br>other = NR	33.25 (9.82)	33.00%	bot:<br>virtually disembodied	vignette-described partner	moral decision-making in service encounter simulation	2	2
48	(Go, 2019)[49] S1	B	141<br>HAI = NR<br>HHI = NR	33.91 (10.09)	49.60%	conversational agent:<br>virtually disembodied	research team member	camera purchase recommendation	1	1
49	(Gonzalez-Billandon, 2019)[50] S1	W	25<br>HAI = 25<br>HHI = 25	24.50 (5.31)	65.38%	iCub robot:<br>physically embodied	research team member	mock interrogation task	2	2
50	(Gratch, 2016; Mozgai, 2017)[51,52] S1	B	93<br>HAI = 49<br>HHI = 44	NR	55.91%	virtual human:<br>virtually embodied	research team member	negotiation training (with cooperative and competitive negotiation scenarios)	7	5
51	(Guadagno, 2007)[53] S2	B	174<br>HAI = NR<br>HHI = NR	NR	51.15%	virtual human:<br>virtually embodied	pseudo-human	persuasive speech listening	2	2
52	(Guadagno,	B	38	20.20	100.00%	virtual human:	research team	self-disclosure task	1	1

No	Study	HAI vs. HHI	Sample size	Age, M (SD)	% Female	Agent partner	Human partner	Interaction task	ESs calculated	ESs in meta-analysis
	2011)[54] S1		HAI = NR<br>HHI = NR	(1.55)		virtually embodied	member			
53	(Haring, 2019)[55] S1	B	72<br>HAI1 = 24<br>HAI2 = 28<br>HHI = 20	18.60 (0.75)	48.10%	NAO/Roomba robot: physically embodied	research team member	training compliance task	8	4
54	(Harriott, 2013)[56] S2	B	36<br>HAI = 18<br>HHI = 18	25.75 (6.88)	47.22%	Pioneer 3-DX robot: physically embodied	research team member	collaborative decision-making task	10	5
55	(Hertz, 2016)[57] S1	W	62<br>HAI = 62<br>HHI = 62	19.33	64.52%	Meka robot: physically embodied	pseudo-human	Asch line judgment task	1	1
56	(Hinds, 2004)[58] S1	B	293<br>HAI1 = 98<br>HAI2 = 97<br>HHI = 98	20.51	59.00%	no-name robot: physically embodied	research team member	collaborative parts collection task	10	10
57	(Ho, 2018)[59] S1	B	98<br>HAI = NR<br>HHI = NR	22.00	68.75%	conversational agent: virtually disembodied	research team member	emotional or factual self-disclosure task	9	9
58	(Hoffman, 2015)[60] S1	B	40<br>HAI = 20<br>HHI = 20	27.00 (8.78)	67.50%	no-name robot: physically embodied	research team member	perceptual task being monitored by an agent/human	8	3
59	(Holthöwer, 2023)[61] S3	B	380<br>HAI = 195<br>HHI = 185	25.54 (8.22)	41.84%	Pepper robot: physically embodied	vignette-described partner	restaurant service simulation	1	1
60	(Holthöwer, 2023)[61] S4	B	407<br>HAI = 205<br>HHI = 202	27.48 (9.32)	44.47%	Pepper robot: physically embodied	vignette-described partner	pharmacy service simulation	2	0
61	(Hoorn, 2024)[62] S1	B	69<br>HAI1 = 24<br>HAI2 = 10<br>HAI3 = 20<br>HHI = 15	42.00 (13.58)	60.67%	Sophia/Iwaa robot: physically embodied virtual agent with Sophia shape: virtually embodied	research team member	seminar check-in	12	6
62	(Horstmann, 2021)[63] S1	B	130<br>HAI = 64	34.80 (13.16)	47.69%	virtual human: virtually embodied	research team member	tutorial (with an agent/human giving negative feedback on	13	13

No	Study	HAI vs. HHI	Sample size	Age, M (SD)	% Female	Agent partner	Human partner	Interaction task	ESs calculated	ESs in meta-analysis
			HHI = 66					participant's performance)		
63	(Huang, 2015)[64] S1	W	9<br>HAI = 9<br>HHI = 9	NR	44.44%	virtual human: virtually embodied	participant	speaking task (with an agent/human as the active listener)	1	1
64	(Jerčić, 2018)[65] S1	W	70<br>HAI = 70<br>HHI = 70	23.56 (2.34)	17.14%	robot hand: physically embodied	research team member	Tower of Hanoi serious game	8	7
65	(Jois, 2021)[66] S1	B	40<br>HAI = 20<br>HHI = 20	NR	70.00%	no-name robot: hybrid combining a physical exoskeleton with a virtual voice interface	research team member	punishment-threat sorting task	5	4
66	(Kahn, 2015)[67] S1	B	54<br>HAI = 27<br>HHI = 27	31.48 (4.26)	55.56%	Robovie-II robot: physically embodied	research team member	lab tour guide task	5	2
67	(Kalashnikova, 2023a)[68] S1	B	63<br>HAI1 = 17<br>HAI2 = 26<br>HHI = 20	NR	63.49%	smart speaker/Pepper robot: physically embodied	research team member	ecological habit conversation	8	0
68	(Kalashnikova, 2023b)[69] S1	B	71<br>HAI1 = 22<br>HAI2 = 28<br>HHI = 21	NR	64.79%	smart speaker/Pepper robot: physically embodied	research team member	ecological habit conversation	6	0
69	(Karpus, 2021)[70] S1	B	351<br>HAI = 180<br>HHI = 171	35.40 (10.50)	39.10%	bot: virtually disembodied	participant	one single-shot game of Trust	4	4
70	(Karpus, 2021)[70] S2	B	201<br>HAI = 101<br>HHI = 100	35.40 (10.50)	39.10%	bot: virtually disembodied	participant	one-shot classical prisoner's dilemma	2	2
71	(Karpus, 2021)[70] S3	B	204<br>HAI = 100,<br>HHI = 104	35.40 (10.50)	39.10%	bot: virtually disembodied	participant	one-shot Chicken game	2	2
72	(Karpus, 2021)[70] S4	B	205<br>HAI = 100<br>HHI = 105	35.40 (10.50)	39.10%	bot: virtually disembodied	participant	one-shot Stag Hunt game	2	2
73	(Karpus,	B	223	35.40	39.10%	bot:	participant	one-shot Reciprocity game	2	2

No	Study	HAI vs. HHI	Sample size	Age, M (SD)	% Female	Agent partner	Human partner	Interaction task	ESs calculated	ESs in meta-analysis
	2021)[70] S9		HAI = 120<br>HHI = 103	(10.50)		virtually disembodied				
74	(Kawai, 2023)[71] S1	W	48<br>HAI = 48<br>HHI = 48	23.50 (2.40)	50.00%	Sota robot: physically embodied	pseudo-human	repeated noncooperative game (similar to the prisoner's dilemma)	4	3
75	(Khalighinejad, 2016)[72] S1	B	64<br>HAI = 31<br>HHI = 33	23.00	70.83%	robot hand: physically embodied	participant	intentional binding task	2	2
76	(Kiilavuori, 2022)[73] S1	B	82<br>HAI = 40<br>HHI = 42	30.00 (11.70)	74.39%	NAO robot: physically embodied	research team member	eye-contact task	2	2
77	(Kiilavuori, 2021)[74] S1	W	42<br>HAI = 42<br>HHI = 42	25.38 (6.29)	69.05%	NAO robot: physically embodied	research team member	eye-contact task	2	2
78	(Kim, 2018)[75] S1/2	B	37<br>HAI = 20<br>HHI = 17	24.63 (3.03)	27.03%	NAO robot: physically embodied	research team member	initiating and responding joint attention tasks	2	1
79	(Kim, 2020)[76] S2	B	324<br>HAI = 162<br>HHI = 162	37.30	56.00%	bot: virtually disembodied	pseudo-human	skin cancer risk assessment and advice task	1	1
80	(Kim, 2020)[76] S5	B	400<br>HAI = 200<br>HHI = 200	35.64	52.00%	NAO robot: physically embodied	pseudo-human	product recommendation	2	2
81	(Kim, 2022)[77] S2	B	160<br>HAI = NR<br>HHI = NR	39.06	49.00%	bot: virtually disembodied	vignette-described partner	product recommendation	1	1
82	(Kim, 2023)[78] S1	B	111<br>HAI = NR<br>HHI = NR	20.50 (1.96)	47.00%	smart speaker: physically embodied	research team member	lottery prediction cheating task	1	1
83	(Kim, 2023)[78] S2	B	128<br>HAI = NR<br>HHI = NR	21.40 (3.84)	44.50%	bot: virtually disembodied	pseudo-human	online shopping task	1	1
84	(Kim, 2023)[78] S3	B	133<br>HAI = NR<br>HHI = NR	39.47 (11.31)	44.40%	bot: virtually disembodied	vignette-described partner	online product return simulation	4	4

No	Study	HAI vs. HHI	Sample size	Age, M (SD)	% Female	Agent partner	Human partner	Interaction task	ESs calculated	ESs in meta-analysis
85	(Krach, 2008)[79] S1	W	20<br>HAI1 = 20<br>HAI2 = 20<br>HHI = 20	24.50 (2.97)	0.00%	BARTHOC Jr./no-name robot: physically embodied	research team member	iterated prisoner’s dilemma	10	8
86	(Kulms, 2019)[80] S1	B	114<br>HAI = NR<br>HHI = NR<br>other = NR	30.49 (10.99)	41.20%	virtual human: virtually embodied	pseudo-human	cooperative 2-player puzzle game	5	5
87	(Leiño Calleja, 2023)[81] S1	B	124<br>HAI = 57<br>HHI = 67	39.28	65.32%	Pepper robot: physically embodied	vignette-described partner	shopping simulation	3	3
88	(Leiño Calleja, 2023)[81] S2	B	286<br>HAI = NR<br>HHI = NR	34.05	73.78%	Pepper robot: physically embodied	vignette-described partner	shopping simulation	3	3
89	(Leiño Calleja, 2023)[81] S3b	B	252<br>HAI = NR<br>HHI = NR	34.41	73.02%	Pepper robot: physically embodied	vignette-described partner	shopping simulation	3	3
90	(Leo, 2020)[82] S1a	B	199<br>HAI = NR<br>HHI = NR	38.00 (12.52)	74.00%	info-missing robot: physically embodied	vignette-described partner	pharmacy service simulation	2	2
91	(Leo, 2020)[82] S2	B	206<br>HAI = NR<br>HHI = NR	35.01 (10.79)	52.70%	info-missing robot: physically embodied	vignette-described partner	restaurant service simulation	2	2
92	(Liao, 2024)[83] S2	B	117<br>HAI = NR<br>HHI = NR	35.07 (12.95)	34.20%	no-name robot: physically embodied	vignette-described partner	hotel service simulation	1	1
93	(Liao, 2023)[84] S1	B	178<br>HAI = 102<br>HHI = 76	19.84 (2.23)	78.50%	conversation agent: virtually disembodied	participant	sleep advice seeking conversation (without emotional support)	5	4
94	(Liao, 2023)[84] S2	B	180<br>HAI = 89<br>HHI = 91	19.79 (2.26)	83.08%	conversation agent: virtually disembodied	participant	sleep advice seeking conversation (with emotional support)	5	4
95	(Liu, 2024)[85] S1a	B	211<br>HAI = 107<br>HHI = 104	43.40 (13.43)	49.76%	conversational agent: virtually disembodied	pseudo-human	health insurance plan recommendation	3	2

No	Study	HAI vs. HHI	Sample size	Age, M (SD)	% Female	Agent partner	Human partner	Interaction task	ESs calculated	ESs in meta-analysis
96	(Liu, 2024)[85] S1b	B	224<br>HAI = 111<br>HHI = 113	45.45 (17.63)	51.79%	conversational agent: virtually disembodied	pseudo-human	health insurance plan recommendation	3	2
97	(Lin, 2022)[86] S1	B	42<br>HAI = 21<br>HHI = 21	20.00 (1.31)	42.86%	Anki Vector robot: physically embodied	research team member	role-playing puzzle	15	13
98	(Liu, 2020)[87] S1	W	16<br>HAI = 16<br>HHI = 16	21.20 (1.20)	50.00%	smart speaker: physically embodied	research team member	modular arithmetic task (assisted by an agent/human)	2	2
99	(Maehigashi, 2022)[88] S1	B	258<br>HAI1 = 89<br>HAI2 = 86<br>HHI = 83	47.24 (10.49)	26.36%	bot:<br>virtually disembodied<br>Palro robot:<br>physically embodied	pseudo-human	calculation and emotion recognition tasks (with advice from an agent/human)	6	4
100	(Maggioni, 2023)[89] S1	B	305<br>HAI = 152<br>HHI = 153	NR	69.68%	NAO robot: physically embodied	research team member	iterated prisoner's dilemma	1	1
101	(Mell, 2017)[90] S1	B	143<br>HAI = 82<br>HHI = 61	35.13 (10.40)	36.75%	conversation agent: virtually disembodied	pseudo-human	prestige question ask & answer task	9	9
102	(Meng, 2021)[91] S1	B	211<br>HAI = NR<br>HHI = NR	20.40 (2.28)	61.60%	conversation agent: virtually disembodied	pseudo-human	stress conversation	3	1
103	(Merkle, 2019)[92] S1	B	120<br>HAI = 60<br>HHI = 60	22.50 (5.20)	43.00%	Pepper robot: physically embodied	research team member	hotel reception check-in (with service failure and success scenarios)	2	2
104	(Merritt, 2011)[93] S1	W	40<br>HAI = 40<br>HHI = 40	21.70	65.00%	bot:<br>virtually disembodied	pseudo-human	Capture the Gunner game	4	4
105	(Mirbabaie, 2021)[94] S1	B	46<br>HAI = 24<br>HHI = 22	23.10 (7.54)	84.00%	conversation agent: virtually disembodied	research team member	Critical Path Method task	3	3
106	(Naito, 2023)[95] S1	B	432<br>HAI = 217<br>HHI = 215	NR	100.00%	Robovie-II robot: physically embodied	vignette-described partner	fashion shopping simulation (with an agent/human service provider using the direct communication strategy)	4	3

No	Study	HAI vs. HHI	Sample size	Age, M (SD)	% Female	Agent partner	Human partner	Interaction task	ESs calculated	ESs in meta-analysis
107	(Naito, 2023)[95] S2	B	467<br>HAI = 218<br>HHI = 249	NR	100.00%	Robovie-II robot: physically embodied	vignette-described partner	fashion shopping simulation (with an agent/human service provider using the data-based strategy)	4	3
108	(Naito, 2023)[95] S3	B	456<br>HAI = 219<br>HHI = 237	NR	100.00%	Robovie-II robot: physically embodied	vignette-described partner	fashion shopping simulation (with an agent/human service provider using the indirect communication strategy)	4	3
109	(Ng, 2023)[96] S1	B	164<br>HAI = 87<br>HHI = 77	36.42 (10.65)	33.54%	conversation agent: virtually disembodied	pseudo-human	iterated prisoner's dilemma	3	3
110	(Nishio, 2012)[97] S1	W	21<br>HAI1 = 21<br>HAI2 = 21<br>HHI = 21	21.20 (2.56)	42.86%	Robovie-R2/ Geminoid HI-1 robot: physically embodied	research team member	truncated, multiperiod ultimatum game	4	4
111	(Numata, 2020)[98] S1	W	39<br>HAI = 39<br>HHI = 39	21.49 (1.19)	48.72%	virtual agent with chick shape: virtually embodied	pseudo-human	facial interaction task	8	6
112	(Ossadnik, 2023)[99] S1	B	153<br>HAI = 73<br>HHI = 80	23.75 (3.11)	55.50%	conversation agent: virtually disembodied	vignette-described partner	side job help-seeking simulation	2	1
113	(Palanica, 2019)[100] S1	W	46<br>HAI = 46<br>HHI = 46	33.60 (7.20)	54.35%	Pepper robot: physically embodied	research team member	verbal comprehension test	4	4
114	(Pavone, 2023)[101] S1	B	122<br>HAI = 61<br>HHI = 61	NR	66.40%	conversation agent: virtually disembodied	vignette-described partner	service failure simulation (luggage missing in air travel)	1	1
115	(Pickard, 2020)[102] S1	B	290<br>HAI1 = 58<br>HAI2 = 58<br>HAI3 = 58<br>HAI4 = 58<br>HHI = 58	21.87 (3.51)	47.59%	virtual human: virtually embodied	research team member	mock job interview	28	24
116	(Plaks, 2022)[103] S1	B	811<br>HAI = 407	35.00	NR	Pepper robot: physically embodied	pseudo-human	two-round, repeated prisoner's dilemma	5	3

No	Study	HAI vs. HHI	Sample size	Age, M (SD)	% Female	Agent partner	Human partner	Interaction task	ESs calculated	ESs in meta-analysis
			HHI = 404							
117	(Poinsot, 2022)[104] S1	B	128<br>HAI = NR<br>HHI = NR	31.45 (13.14)	38.28%	conversation agent: virtually disembodied	pseudo-human	online image recognition game	5	5
118	(Riether, 2012)[105] S1	B	106<br>HAI = NR<br>HHI = NR<br>other = NR	23.31 (2.99)	73.58%	Flobi robot: physically embodied	research team member	cognitive tasks: anagram solving, numerical distance, finger tapping and motoric tracking	4	3
119	(Roozen, 2023)[106] S1	W	425<br>HAI = 425<br>HHI = 425	30.85 (8.60)	42.40%	Pepper robot: physically embodied	vignette-described partner	shopping simulation	7	7
120	(Russo, 2021)[107] S1	B	403<br>HAI = NR<br>HHI = NR<br>other = NR	27.74 (10.01)	60.50%	bot: virtually disembodied	pseudo-human	ultimatum game	1	1
121	(Ryoo, 2024)[108] S1A	B	196<br>HAI = 91<br>HHI = 105	38.68	59.69%	info-missing robot: physically embodied	vignette-described partner	hotel reception service failure simulation	1	1
122	(Ryoo, 2024)[108] S1B	B	190<br>HAI = NR<br>HHI = NR	39.25	61.05%	info-missing robot: physically embodied	vignette-described partner	hotel reception service failure simulation	1	1
123	(Ryoo, 2024)[108] S2	B	226<br>HAI1 = NR<br>HAI2 = NR<br>HHI = NR	38.81	52.21%	no-name robot: physically embodied	vignette-described partner	restaurant service failure simulation	4	4
124	(Ryoo, 2024)[108] S3	B	233<br>HAI1 = NR<br>HAI2 = NR<br>HHI = NR	40.24	54.08%	no-name robot: physically embodied	vignette-described partner	restaurant service failure simulation	4	4
125	(Sandoval, 2016)[109] S1	B	60<br>HAI = 30<br>HHI = 30	26.50 (6.50)	35.00%	NAO robot: physically embodied	research team member	iterated prisoner's dilemma ultimatum game	6	4
126	(Sahaï, 2023)[110] S1	W	28<br>HAI = 28<br>HHI = 28	23.61 (3.52)	78.57%	Pepper robot: physically embodied	participant	joint Simon task	1	1

No	Study	HAI vs. HHI	Sample size	Age, M (SD)	% Female	Agent partner	Human partner	Interaction task	ESs calculated	ESs in meta-analysis
127	(Sciutti, 2013)[111] S1	W	8<br>HAI = 8<br>HHI = 8	31.00 (13.00)	20.00%	iCub robot: physically embodied	research team member	object transport observation	1	1
128	(Shahverdi, 2023)[112] S1	B	28<br>HAI = 14<br>HHI = 14	30.00	46.43%	Furhat robot: physically embodied	research team member	interactive storytelling	3	0
129	(Shen, 2022)[113] S1	B	223<br>HAI = 113<br>HHI = 110	19.90 (1.52)	65.02%	conversation agent: virtually disembodied	pseudo-human	text-based picture naming and matching task (collaboration task)	5	4
130	(Shin, 2023)[114] S1	B	117<br>HAI = 55<br>HHI = 62	23.30	67.50%	conversation agent: virtually disembodied	vignette-described partner	online service encounter simulation	1	1
131	(Sundar, 2019)[115] S1	B	157<br>HAI = 76<br>HHI = 81	38.29 (12.51)	46.30%	conversation agent: virtually disembodied	vignette-described partner	airline ticket booking simulation	1	1
132	(Singh, 2021)[116] S1	B	86<br>HAI = NR<br>HHI = NR	38.80 (11.60)	51.16%	Pepper robot: physically embodied	vignette-described partner	conference registration simulation	2	2
133	(Smyk, 2018)[117] S1	W	20<br>HAI = 20<br>HHI = 20	19.70 (2.34)	100.00%	no-name robot: physically embodied	research team member	reciprocal touch task	1	1
134	(Söderlund, 2020)[118] S1	B	338<br>HAI = 166<br>HHI = 172	38.48	73.08%	conversation agent: virtually disembodied	vignette-described partner	movie recommendation simulation	6	3
135	(Stock, 2018)[119] S1	B	132<br>HAI = 66<br>HHI = 66	21.80 (5.68)	47.73%	Pepper robot: physically embodied	research team member	hotel reception check-in	2	2
136	(Stock-Homburg, 2020)[120] S1	B	210<br>HAI1 = 71<br>HAI2 = 66<br>HHI = 73	39.70 (10.76)	NR	Pepper/Elenoide robot: physically embodied	research team member	HR inquiry task	4	2
137	(Tatsukawa, 2018)[121] S1	B	44<br>HAI1 = 15<br>HAI2 = 15<br>HHI = 14	20.50 (1.50)	40.91%	Geminoid-F/no-name robot: physically embodied	research team member	colour perception task (Asch-like task)	2	2

No	Study	HAI vs. HHI	Sample size	Age, M (SD)	% Female	Agent partner	Human partner	Interaction task	ESs calculated	ESs in meta-analysis
138	(Takahashi, 2014)[122] S1	W	20<br>HAI = 20<br>HHI = 20	NR	75.00%	Actroid-F robot: physically embodied	pseudo-human	repeated penny-matching game (preceded by a brief first-impression chat)	1	1
139	(Terada, 2017)[123] S2	B	85<br>HAI1 = NR<br>HAI2 = NR<br>HHI = NR<br>other = NR	21.56 (1.45)	43.53%	bear-shape/NAO robot:<br>physically embodied	pseudo-human	repeated penny-matching game	2	2
140	(Torta, 2013)[124] S1	B	138<br>HAI = NR<br>HHI = NR<br>other = NR	25.68 (11.45)	42.03%	no-name robot: physically embodied	pseudo-human	multiple (not repeated) ultimatum games	3	2
141	(Tsfasman, 2021)[125] S1	B	43<br>HAI = 22<br>HHI = 21	28.00 (10.00)	51.16%	Furhat robot: physically embodied	research team member	tutorial	16	13
142	(Tu, 2023)[126] S1	B	277<br>HAI = NR<br>HHI = NR	NR	60.60%	conversation agent: virtually disembodied	vignette-described partner	insurance transaction simulation	3	3
143	(Tuvo, 2022)[127] S1	W	30<br>HAI1 = 30<br>HAI2 = 30<br>HHI = 30	23.46 (2.37)	73.33%	iCub/Kuka robot: physically embodied	pseudo-human	Ballon Risk-Taking task (the diffusion of responsibility task)	4	2
144	(Von Der Pütten, 2010)[128] S1	B	83<br>HAI = 41<br>HHI = 42	37.27 (13.61)	50.60%	virtual human: virtually embodied	research team member	self-story telling	1	1
145	(Walliser, 2015)[129] S1	W	66<br>HAI = 66<br>HHI = 66	36.00	60.61%	Eddie robot: physically embodied	pseudo-human	investment game, ultimatum game, dictator game	5	4
146	(Wang, 2023)[130] S1	B	177<br>HAI = NR<br>HHI = NR	27.11 (5.41)	53.63%	conversation agent: virtually disembodied	vignette-described partner	online shopping simulation	2	2
147	(Wang, 2023)[130] S2	W	35<br>HAI = 35<br>HHI = 35	21.43 (2.32)	51.43%	conversation agent: virtually disembodied	vignette-described partner	ERP-modified online shopping simulation	1	1

No	Study	HAI vs. HHI	Sample size	Age, M (SD)	% Female	Agent partner	Human partner	Interaction task	ESs calculated	ESs in meta-analysis
148	(Xiao, 2023)[131] S1	B	33<br>HAI = 16<br>HHI = 17	21.32 (2.16)	60.61%	info-missing robot: physically embodied	vignette-described partner	location describing task	7	2
149	(Xiao, 2023)[131] S2	B	30<br>HAI = 15<br>HHI = 15	21.46 (2.32)	63.33%	info-missing robot: physically embodied	vignette-described partner	route describing task	7	3
150	(Xiao, 2021)[132] S1	B	64<br>HAI = NR<br>HHI = NR	22.50 (2.30)	62.50%	info-missing robot: physically embodied	vignette-described partner	spatial describing task	6	3
151	(Xu, 2018a; Xu, 2018b)[133,134] S1	B	20<br>HAI = 10<br>HHI = 10	20.30	55.00%	NAO robot: physically embodied	research team member	SuperPop VR (therapeutic game)	10	8
152	(Yu, 2012)[135] S1	B	42<br>HAI = 21<br>HHI = 21	NR	NR	no-name robot: physically embodied	research team member	joint attention task	6	0
153	(Yu, 2024)[136] S1	B	204<br>HAI = NR<br>HHI = NR	36.38 (12.06)	69.00%	conversation agent: virtually disembodied	vignette-described partner	customer service failure simulation	2	2
154	(Yu, 2024)[136] S2	B	95<br>HAI = NR<br>HHI = NR	22.24	73.00%	conversation agent: virtually disembodied	research team member	service failure encounter	1	1
155	(Yu, 2024)[136] S3	B	202<br>HAI = NR<br>HHI = NR	23.92 (2.24)	100.00%	conversation agent: virtually disembodied	research team member	service failure encounter	1	1
156	(Yu, 2024)[136] S4B	B	701<br>HAI = NR<br>HHI = NR	38.00 (12.60)	67.00%	conversation agent: virtually disembodied	vignette-described partner	customer service encounter simulation (with service failure and success scenarios)	2	2
157	(Yu, 2024)[136] S5	B	506<br>HAI = NR<br>HHI = NR	37.83 (11.54)	39.00%	conversation agent: virtually disembodied	vignette-described partner	customer service failure (with emotional or non-emotional apology) simulation	6	6
158	(Yu, 2024)[137] S1	B	550<br>HAI = 271<br>HHI = 279	30.40 (7.26)	61.00%	conversation agent: virtually disembodied	vignette-described partner	online customer service simulation	3	3

No	Study	HAI vs. HHI	Sample size	Age, M (SD)	% Female	Agent partner	Human partner	Interaction task	ESs calculated	ESs in meta-analysis
159	(Zhang, 2023)[138] S1	B	128<br>HAI = 64<br>HHI = 64	NR	NR	bot:<br>virtually disembodied	pseudo-human	four-step chess puzzle	10	10
160	(Zhou, 2022)[139] S2	B	160<br>HAI = 80<br>HHI = 80	25.72 (5.56)	55.00%	conversation agent:<br>virtually disembodied	vignette-described partner	charitable project donation simulation	3	2
161	(Zhou, 2022)[139] S3	B	200<br>HAI = NR<br>HHI = NR	21.75 (3.53)	65.50%	conversation agent:<br>virtually disembodied	vignette-described partner	charitable project donation simulation	2	2
162	(Zonca, 2023)[140] S1	B	50<br>HAI = 25<br>HHI = 25	32.96 (12.30)	56.00%	iCub robot:<br>physically embodied	pseudo-human	joint perceptual decision making task (social influence task)	4	2

Note. The "HAI vs. HHI" column indicated whether each study assigned participants to HAI and HHI conditions using a between-subjects (B) or within-subjects (W) design. The notations S1, S2, etc. refer to individual studies reported within an article. The "ESs calculated" column listed the number of measured responses for which we were able to calculate an effect size (ES), while the "ESs in meta-analysis" column shows how many of those calculated ESs were further included in the quantitative synthesis. HAI refers to human-agent interaction, and HHI refers to human-human interaction. NR = Not Reported.

Supplementary Table 8. Responses with insufficient data for meta-analysis

Responses	*k*	*m*	Narrative summary (Hedges' *g* [95% CI])
**Theme 1: Prosociality and morality**			
No rare responses			
**Theme 2: Social perceptions of interaction partners**			
Perceived positivity	3	4	(Zhou, 2022) S2[139] showed significantly lower perceived positivity of agent vs. human partners (-0.983 [-1.31, -0.66]).<br>(Appel, 2012) S1[21] showed no significant difference in perceived positivity of agent vs. human partners (-0.342 [-0.76, 0.07]).<br>(Tuvo, 2022) S1[127] showed no significant difference in perceived positivity of robot vs. human partners (-0.268 [-0.62, 0.09]), but significantly lower perceived positivity of robotic arm vs. human partners (-0.621 [-1.00, -0.24]).
Perceived negativity	1	2	(Appel, 2012) S1[21] showed no significant difference in perceived negativity of agent vs. human partners (-0.040 [-0.39, 0.32]).
Perceived safety	4	4	(Xiao, 2023) S1, S2[131], (Xiao, 2021) S1[132], and (Jois, 2021) S1[66] showed no significant differences in perceived safety of agent vs. human partners (-0.056 [-0.72, 0.61]; -0.290 [-0.99, 0.41]; -0.241 [-0.73, 0.24]; 0.386 [-0.23, 1.00]).
Perceived animacy	4	4	(Xiao, 2023) S1, S2 showed no significant differences in perceived animacy of agent vs. human partners (-0.504 [-1.18, 0.17]; -0.475 [-1.18, 0.23]).<br>(Xiao, 2021) S1[132] and (Tsfasman, 2021) S1[125] showed significantly lower perceived animacy of agent vs. human partners (-0.808 [-1.31, -0.30]; -0.680 [-1.28, -0.08]).
Perceived credibility	3	3	(Choi, 2019) S1[36] showed no significant difference in perceived credibility of agent vs. human partners (-0.248 [-0.61, 0.12]).<br>(Jerčić, 2018) S1[65] and (Edwards, 2021) S1[45] showed significantly lower perceived credibility of agent vs. human partners (-0.748 [-1.01, -0.49]; -0.936 [-1.47, -0.40]).
Perceived power	2	8	(Hoffman, 2015) S1[60] showed no significant difference in perceived authority of agent vs. human partners (-0.147 [-0.63, 0.34]).<br>(Pickard, 2020) S1[102] showed significantly lower perceived power of agent vs. human partners (-0.698 [-0.99, -0.40]).
Perceived eeriness	1	3	(Hoorn, 2024) S1[62] showed significantly greater perceived eeriness of agent vs. human partners (1.214 [0.60, 1.83]).
Perceived competitiveness	1	2	(Krach, 2008) S1[79] showed no significant difference in perceived competitiveness of agent vs. human partners (0.012 [-0.36, 0.38]).
Perceived ruthlessness	1	1	(Plaks, 2022) S1[103] showed no significant difference in perceived ruthlessness of agent vs. human partners (-0.056 [-0.18, 0.06]).
Perceived naivety	1	1	(Plaks, 2022) S1[103] showed no significant difference in perceived naivety of agent vs. human partners (-0.077 [-0.20, 0.04]).
Perceived uncertainty	1	1	(Edwards, 2019) S1[44] showed significantly lower perceived uncertainty of agent vs. human partners (-0.735 [-1.22, -0.25]).
Perceived agreeableness	1	1	(Sandoval, 2016) S1[109] showed no significant difference in perceived agreeableness of agent vs. human partners (-0.370 [-0.87, 0.13]).
Perceived openness	1	1	(Sandoval, 2016) S1[109] showed significantly lower perceived openness of agent vs. human partners (-0.751 [-1.27, -0.23]).
Experience attribution	4	4	(Kahn, 2015) S1[67], (Liao, 2023) S1, S2[84], and (Kawai, 2023) S1[71] showed significantly lower experience attribution to agent vs. human partners (-2.133 [-2.79, -1.47]; -1.192 [-1.51, -0.87]; -0.881 [-1.19, -0.58]; -1.284 [-1.66, -0.91]).
**Theme 3: Trust in interaction partners**			
Trust decline	1	2	(Maehigashi, 2022) S1[88] showed no significant difference in trust decline in the error trial when interacting with robot vs. human partners (0.242 [-0.06, 0.54]), but a significantly greater trust decline with AI bot vs. human partners (0.351

Responses	*k*	*m*	Narrative summary (Hedges' *g* [95% CI])
			[0.05, 0.65]).
**Theme 4: Social alignment with interaction partners**			
No rare responses			
**Theme 5: Personal agency and task performance**			
Self-privacy concern	4	5	(Liu, 2024) S1a[85] and (Stock-Homburg, 2020) S1 showed significantly greater self-privacy concern when interacting with agent vs. human partners (0.378 [0.11, 0.65]; 0.393 [0.11, 0.68]).<br>(Liu, 2024) S1b[85] and (Söderlund, 2020) S1[118] showed no significant differences in self-privacy concern when interacting with agent vs. human partners (0.143 [-0.12, 0.40]; -0.013 [-0.23, 0.20]).
Estimated self task performance	3	5	(Bartneck, 2008) S1, S2[26] and (Zonca, 2023) S1[140] showed no significant differences in estimated self task performance (i.e., quantitative appraisal of self task performance) when interacting with agent vs. human partners (0.323 [-0.12, 0.77]; 0.189 [-0.19, 0.57]; 0.186 [-0.36, 0.73]).
Subjective evaluation of self task performance	3	6	(Bartneck, 2008) S1, S2[26] and (Gratch, 2016) S1[51] showed no significant differences in subjective evaluation of self task performance (i.e., qualitative appraisal of self task performance) when interacting with agent vs. human partners (-0.310 [-0.76, 0.13]; -0.037 [-0.42, 0.34]; -0.066 [-0.42, 0.29]).
Objective workload	3	7	(Xiao, 2023) S1, S2[131] showed significantly lower objective workload when interacting with agent vs. human partners (-1.009 [-1.72, -0.30]; -0.769 [-1.49, -0.05]).<br>(Harriott, 2013) S2[56] showed no significant differences in several indicators of objective workload (i.e., subtask time, vector magnitude, posture variance, posture mean skewness, and posture mean kurtosis) when interacting with agent vs. human partners (0.551 [-0.10, 1.20]; 0.598 [-0.06, 1.25]; 0.301 [-0.34, 0.94]; 0.023 [-0.62, 0.66]; 0.222 [-0.42, 0.86]).
**Theme 6: Interaction experiences**			
Application of relational models	1	1	(Abril, 2023) S1[19] showed no significant difference in the application of relational models to agent vs. human partners (-0.686 [-1.39, 0.02]).
Affective dominance	1	1	(Banks, 2020) S1[25] showed no significant difference in affective dominance when interacting with agent vs. human partners (-0.236 [-0.85, 0.38]).
Perceived interaction informativeness	3	3	(Naito, 2023) S1, S2[95] showed significantly greater perceived informativeness of interaction with agent vs. human partners (0.249 [0.06, 0.44]; 0.252 [0.07, 0.43]).<br>(Naito, 2023) S3[95] showed significantly lower perceived informativeness of interaction with agent vs. human partners (-0.246 [-0.43, -0.06]).
Perceived interaction smoothness	1	1	(Tsfasman, 2021) S1[125] showed no significant difference in perceived smoothness of interaction with agent vs. human partners (-0.376 [-0.97, 0.22]).
Perceived pressure	4	4	(Bowman, 2019) S1[31], (Xu, 2018) S1[134], and (Ossadnik, 2023) S1[99] showed no significant differences in perceived pressure when interacting with agent vs. human partners (-0.258 [-0.72, 0.21]; -0.064 [-0.90, 0.78]; 0.085 [-0.23, 0.40]).
Perceived task difficulty	1	1	(Caruana, 2019) S1[34] showed significantly greater perceived task difficulty when interacting with agent vs. human partners (0.490 [0.04, 0.94]).
Perceived task usefulness	2	2	(Xu, 2018) S1[134] showed no significant difference in perceived task usefulness when interacting with agent vs. human partners (-0.100 [-0.94, 0.74]).<br>(Čaić, 2020) S1[33] showed significantly lower perceived task usefulness when interacting with agent vs. human partners (-0.727 [-1.27, -0.19]).
Subjective experimental experiences	1	5	(Blankendaal, 2015) S1[29] showed no significant differences in subjective experimental experiences when interacting with agent vs. human partners (-0.178 [-0.90, 0.54]; -0.117 [-0.84, 0.60]; 0.034 [-0.69, 0.75]; -0.055 [-0.77, 0.66]; -0.006 [-0.73, 0.71]).

Responses	*k*	*m*	Narrative summary (Hedges' *g* [95% CI])
**Unclassified responses**			
Perceived monitoring	3	4	(Riether, 2012) S1[105] showed significantly greater perceived monitoring by agent vs. human partners (0.547 [0.08, 1.02]).<br>(Lin, 2022) S1[86] showed no significant difference in perceived watching by agent vs. human partners (-0.442 [-1.04, 0.16]).<br>(Hoffman, 2015) S1[60] showed no significant difference in perceived monitoring by agent vs. human partners (0.393 [-0.22, 1.01]), but the evaluation of being monitored differed significantly (0.796 [0.16, 1.43]).
Social evaluative concern	3	4	(Chen, 2023) S3[35], (Holthöwer, 2023) S4[61], and (Lin, 2022) S1[86] showed significantly lower social evaluative concern when interacting with agent vs. human partners (-1.050 [-1.42, -0.68]; -0.401 [-0.60, -0.21]; -0.673 [-1.28, -0.06]).
Compliance (with explicit requests)	1	4	(Haring, 2019) S1[55] showed significantly lower behavioural compliance with explicit requests from agent vs. human partners (-1.978 [-2.53, -1.42]).
Estimated partner task performance	4	6	(Bartneck, 2008) S1, S2[26] and (Zonca, 2023) S1[140] show no significant differences in estimated task performance by agent vs. human partners (0.136 [-0.30, 0.57]; -0.037 [-0.42, 0.34]; 0.416 [-0.14, 0.97]).<br>(Shen, 2022) S1[113] showed significantly lower estimated task performance by agent vs. human partners (-0.431 [-0.70, -0.17]).
Subjective evaluation of partner task performance	3	5	(Bartneck, 2008) S1, S2[26] showed significantly greater satisfaction with task performance by agent vs. human partners (0.758 [0.26, 1.25]; 0.584 [0.17, 1.00]).<br>(Söderlund, 2020) S1[118] showed no significant difference in perceived effort by agent vs. human partners (-0.090 [-0.30, 0.12]).
Punishment behaviour	2	8	(Bartneck, 2008) S1[26] showed no significant differences in punishment frequency and intensity towards agent vs. human partners (-0.247 [-0.69, 0.19]; -0.441 [-0.89, 0.01]).<br>(Bartneck, 2008) S2[26] showed no significant differences in punishment frequency and intensity towards agent vs. human partners (-0.084 [-0.46, 0.30]; 0.005 [-0.37, 0.38]).
Self-reported punishment behaviour	2	8	(Bartneck, 2008) S1[26] showed no significant differences in self-reported punishment frequency and intensity towards agent vs. human partners (0.039 [-0.39, 0.47]; -0.303 [-0.74, 0.14]).<br>(Bartneck, 2008) S2[26] showed no significant differences in self-reported punishment frequency and intensity towards agent vs. human partners (0.022 [-0.36, 0.40]; -0.159 [-0.54, 0.22]).
Praise behaviour	2	8	(Bartneck, 2008) S1[26] showed no significant differences in praise frequency and intensity towards agent vs. human partners (0.141 [-0.29, 0.57]; 0.267 [-0.17, 0.71]).<br>(Bartneck, 2008) S2[26] showed no significant differences in praise frequency and intensity towards agent vs. human partners (0.358 [-0.03, 0.75]; 0.066 [-0.31, 0.45]).
Self-reported praise behaviour	2	8	(Bartneck, 2008) S1[26] showed no significant differences in self-reported praise frequency and intensity towards agent vs. human partners (-0.236 [-0.67, 0.20]; -0.068 [-0.50, 0.36]).<br>(Bartneck, 2008) S2[26] showed no significant differences in self-reported praise frequency and intensity towards agent vs. human partners (-0.212 [-0.60, 0.17]; -0.172 [-0.55, 0.21]).
Intention to acquire medicine	1	1	(Holthöwer, 2023) S4[61] showed no significant difference in intention to acquire embarrassing medicine from agent vs. human partners (-0.186 [-0.38, 0.01]).
Offer acceptance	4	9	(Garvey, 2023) S1a, S3a[47] showed no significant differences in offer acceptance from agent vs. human partners when offers met expectations (-0.183 [-0.65,

Responses	*k*	*m*	Narrative summary (Hedges' *g* [95% CI])
			0.29]; 0 [-2.16, 2.16]), but significantly greater acceptance from agent vs. human partners when offers were worse-than-expected (0.771 [0.24, 1.30]; 0.483 [0.14, 0.82]).<br>(Garvey, 2023) S1b[47] showed no significant difference in offer acceptance from agent vs. human partners when offers met expectations (0.072 [-0.30, 0.44]), but significantly lower acceptance from agent vs. human partners when offers were better-than-expected (-0.573 [-1.06, -0.08]).<br>(Garvey, 2023) S2[47] showed no significant differences in offer acceptance from agent vs. human partners when offers met expectations (-0.154 [-0.41, 0.10]) and when offers were better-than-expected (-0.245 [-0.50, 0.01]), but significantly greater acceptance from agent vs. human partners when offers were worse-than-expected (0.364 [0.11, 0.62]).
Partner conflict-resolution strategy acceptance	2	90	(Babel, 2021) S1[22] showed no significant differences in acceptance of various positive, negative, and neutral conflict-resolution strategies by CR700/Roomba/Pepper robot vs. human partners. Exceptions included: significantly lower acceptance of the positive-appeal strategy by CR700/Roomba/Pepper robot vs. human partners (-0.924 [-1.64, -0.21]; -0.837 [-1.54, -0.13]; -0.913 [-1.62, -0.20]); significantly lower acceptance of positive-empathy and positive-apologise strategies by CR700 robot vs. human partners (-0.784 [-1.42, -0.15]; -0.582 [-1.16, -0.002]); significantly lower acceptance of the negative-threat strategy by CR700/Roomba robot vs. human partners (-0.796 [-1.36, -0.23]; -0.787 [-1.35, -0.22]); significantly greater acceptance of the negative-physical-contact strategy by Roomba robot vs. human partners (0.908 [0.20, 1.62]); and significantly lower acceptance of the neutral-show-benefit strategy by CR700 robot vs. human partners (-0.772 [-1.43, -0.11]).<br>(Babel, 2021)[22] S2 showed no significant differences in acceptance of all positive, negative, and neutral conflict-resolution strategies by Roomba/Tiago/ Pepper robot vs. human partners.
Engagement behaviours	3	9	(Kahn, 2015) S1[67] showed significantly less extended verbal behaviour (i.e., responses that extend the dialogue between partners and participants "beyond minimally required ways") when interacting with agent vs. human partners (-1.285 [-1.86, -0.71]), but no significant difference in rich verbal behaviour (i.e., responses that deepen the dialogue between partners and participants "by moving beyond mere politeness, and are characterized by genuine interest and engagement") with agent vs. human partners (-0.330 [-0.86, 0.20]).<br>(Shahverdi, 2023) S1[112] showed no significant differences in backchanneling towards neutral and negative-emotion narratives when interacting with agent vs. human partners (0.373 [-0.35, 1.10]; 0.613 [-0.12, 1.35]), but significantly greater backchanneling towards positive-emotion narratives with agent vs. human partners (0.756 [0.01, 1.50]).<br>(De Carolis, 2014) S1[39] showed significantly fewer initiative behaviours when interacting with agent vs. human partners (-1.175 [-1.80, -0.55]), but no significant difference in average social moves with agent vs. human partners (-0.604 [-1.32, 0.11]).
Eye gaze	3	4	(Desideri, 2019) S1[43] and (Kim, 2018) S1[75] showed significantly more eye gaze directed at agent vs. human partners (1.314 [0.89, 1.74]; 1.699 [0.83, 2.56]).
Linguistic features	3	9	(Appel, 2012) S1[21] showed no significant difference in word count when interacting with agent vs. human partners (0.342 [-0.07, 0.76]).<br>(Meng, 2021) S1[91] showed no significant difference in the use of informal language when interacting with agent vs. human partners (-0.254 [-0.52, 0.02]), but significantly fewer netspeak words with agent vs. human partners (-0.325 [-0.60, -0.05]).<br>(Yu, 2012) S1[135] showed no significant difference in the number of utterances

Responses	*k*	*m*	Narrative summary (Hedges' *g* [95% CI])
			when interacting with agent vs. human partners (0.339 [-0.26, 0.94]), but significantly lower numbers of distinct word types, tokens, one-word naming utterances, and words per utterances with agent vs. human partners (-1.550 [-2.23, -0.87]; -1.768 [-2.47, -1.06]; -1.372 [-2.03, -0.71]; -0.960 [-1.59, -0.33]); also, this study showed significantly more one-word utterances with agent vs. human partners (1.078 [0.44, 1.71]).
Paralinguistic features	3	15	(Appel, 2012) S1[21] showed no significant difference in speech disfluencies when interacting with agent vs. human partners (-0.060 [-0.47, 0.35]). (Kalashnikova, 2023a) S1[68] showed significantly larger pitch range for both females and males when interacting with smart speaker vs. human partners (1.380 [0.46, 2.30]; 1.358 [0.22, 2.50]), and with robot vs. human partners (2.231 [1.36, 3.10]; 2.245 [0.93, 3.56]); also, this study showed significantly higher mean pitch for both females and males with robot vs. human partners (2.759 [1.80, 3.72]; 2.830 [1.37, 4.30]), but with smart speaker vs. human partners, females' mean pitch did not differ (0.325 [-0.51, 1.16]), while males' mean pitch was significantly higher (1.948 [0.70, 3.20]). (Kalashnikova, 2023b) S1[69] showed significantly shorter utterances and lower speech rate when interacting with smart speaker vs. human partners (-0.656 [-1.26, -0.05]; -1.027 [-1.65, -0.40]), and with robot vs. human partners (-0.815 [-1.39, -0.24]; -0.795 [-1.37, -0.22]); also, this study showed significantly fewer filled pauses with smart speaker vs. human partners (-0.874 [-1.49, -0.26]), but no significant difference in the use of filled pauses with robot vs. human partners (-0.227 [-0.79, 0.33]).
Kinematic features	1	5	(Bah, 2022) S1[23] showed no significant differences in time to max aperture and time to peak velocity at the grabbing phrase when interacting with agent vs. human partners (-0.448 [-1.02, 0.13]; -0.183 [-0.73, 0.37]), while significantly longer length of wrist pathway with agent vs. human partners (0.774 [0.14, 1.41]); also, this study showed significantly lower max velocity and less time to peak velocity at the dropping phrase with agent vs. human partners (-0.858 [-1.51, -0.21]; -0.722 [-1.35, -0.10]).

Note. Effect sizes (Hedges' *g* [95% CI]) for all outcomes were calculated as the unadjusted estimates of partner effects (agent vs. human partners). In some instances, the statistical significance derived from these calculated effect sizes (based on 95% CI interpretation) differed from the significance tests reported in the original studies (highlighted in orange). These discrepancies primarily arose because the original analyses employed complex models (e.g., multifactor ANOVAs, regressions, and covariate-adjusted models), applied *p*-value adjustments (e.g., Tukey HSD), or used alterative approaches (e.g., non-parametric tests), or there were potential inconsistencies in original statistical reporting or data visualisation.

Supplementary Table 9. Univariate meta-regressions and subgroup analyses for different response types

Responses	Moderators	*F*	*F_p*	*QE*	*QE_p*	I^2 (%)	BF_{10}	*k*	*m*
**Theme 1: Prosociality and morality**									
Prosocial behaviour	*non-significant heterogeneity (Q = 10.83, p = 0.288); insufficient studies (k = 9, m = 10)*								
Moral engagement	*non-significant heterogeneity (Q = 4.04, p = 0.991); insufficient studies (k = 8, m = 14)*								
**Theme 2: Social perceptions of interaction partners**									
Perceived social presence	*insufficient studies (k = 9, m = 20)*								
Perceived likeability (20 tested moderators)	Appearance difference	7.48	**0.011**	367.63	< 0.001	86.03	4.559	28	41
	Appearance differed (*k* = 17, *m* = 27): -0.539 [-0.81, -0.27]; *t* = -4.23, *p* < 0.001<br>Appearance matched (*k* = 11, *m* = 14): -0.062 [-0.27, 0.15]; *t* = -0.66, *p* = 0.524								
	Interaction task	5.16	**0.007**	227.85	< 0.001	83.74	4.839	26	38
	Service encounter (*k* = 9, *m* = 11): -0.587 [-0.87, -0.30]; *t* = -4.74, *p* = 0.001<br>Game (*k* = 4, *m* = 5): -0.825 [-1.19, -0.46]; *t* = -7.16, *p* = 0.006<br>Instructional interaction (*k* = 4, *m* = 5): 0.101 [-0.62, 0.82]; *t* = 0.44, *p* = 0.688<br>Communication-focused interaction (*k* = 9, *m* = 17): -0.130 [-0.50, 0.24]; *t* = -0.81, *p* = 0.440								
	Agent form	5.92	**0.020**	209.98	< 0.001	85.99	2.740	27	40
	Interaction realism	4.24	**0.050**	288.62	< 0.001	86.80	1.480	28	41
	Study setting	2.04	0.166	304.93	< 0.001	89.29	0.622	26	37
	Study design	1.34	0.258	443.07	< 0.001	88.38	0.495	28	41
	Publication year	0.00	0.952	420.40	< 0.001	88.79	0.103	28	41
	Sample continent	0.49	0.493	138.46	< 0.001	84.31	0.275	18	29
	Sample WEIRD	1.29	0.269	203.05	< 0.001	84.37	0.376	23	36
	Main age	1.47	0.238	302.39	< 0.001	88.16	0.232	25	38
	Percentage female	0.26	0.615	441.69	< 0.001	88.65	0.119	28	41
	Human partner type	1.14	0.337	290.52	< 0.001	87.89	0.152	28	41
	Agent operationalisation	2.29	0.122	296.34	< 0.001	87.39	0.381	28	41
	Agent embodiment	0.19	0.668	429.23	< 0.001	89.15	0.301	27	40
	Voice difference	0.97	0.330	435.49	< 0.001	88.45	0.273	28	41
	Interaction flow	0.05	0.817	443.01	< 0.001	88.75	0.269	28	41
	Interaction medium	4.02	0.056	224.17	< 0.001	87.23	1.409	26	39
	Interaction structure	0.88	0.357	439.50	< 0.001	88.50	0.404	28	41
	Interaction nature	0.86	0.363	286.47	< 0.001	88.52	0.488	24	35
	Power symmetry	0.11	0.744	440.88	< 0.001	88.77	0.326	28	41
Perceived competence (19 tested moderators)	Agent form	13.42	**0.001**	68.06	< 0.001	63.53	16.598	22	30
	Physical (*k* = 13, *m* = 16): -0.669 [-0.82, -0.52]; *t* = -9.69, *p* < 0.001<br>Virtual (*k* = 10, *m* = 14): -0.233 [-0.44, -0.02]; *t* = -2.49, *p* = 0.035								
	Appearance difference	12.49	**0.002**	73.96	< 0.001	65.32	19.646	23	31
	Appearance differed (*k* = 15, *m* = 22): -0.614 [-0.76, -0.47]; *t* = -8.91, *p* < 0.001<br>Appearance matched (*k* = 8, *m* = 9): -0.170 [-0.43, 0.09]; *t* = -1.54, *p* = 0.167								
	Interaction medium	12.08	**0.002**	72.84	< 0.001	66.88	14.841	22	30
	Computer-mediated (*k* = 13, *m* = 18): -0.275 [-0.48, -0.07]; *t* = -2.87, *p* = 0.014<br>Face-to-face (*k* = 9, *m* = 12): -0.712 [-0.86, -0.57]; *t* = -11.48, *p* < 0.001								
	Study setting	2.13	0.161	129.53	< 0.001	79.65	0.480	21	27
	Study design	0.00	0.975	148.82	< 0.001	78.40	0.212	23	31
	Publication year	0.16	0.692	148.76	< 0.001	78.37	0.105	23	31
	Sample continent	1.41	0.259	20.35	0.257	32.07	0.291	13	19

Responses	Moderators	*F*	*F_p*	*QE*	*QE_p*	I^2 (%)	BF_{10}	*k*	*m*
	Sample WEIRD	1.01	0.329	75.07	< 0.001	69.65	0.318	18	26
	Main age	1.18	0.291	135.93	< 0.001	78.25	0.154	22	30
	Percentage female	0.58	0.457	142.76	< 0.001	77.78	0.125	23	31
	Human partner type	0.74	0.491	124.60	< 0.001	78.13	0.091	21	29
	Agent operationalisation	1.34	0.285	117.75	< 0.001	75.77	0.134	23	31
	Agent embodiment	1.38	0.254	119.80	< 0.001	77.79	0.372	22	30
	Voice difference	0.86	0.361	148.71	< 0.001	78.83	0.193	23	31
	Interaction realism	3.54	0.074	123.89	< 0.001	74.92	0.836	23	31
	Interaction flow	1.11	0.304	145.82	< 0.001	77.45	0.387	23	31
	Interaction structure	2.12	0.160	140.57	< 0.001	76.71	0.507	23	31
	Interaction nature	1.44	0.246	114.12	< 0.001	78.98	0.516	20	23
	Interaction task	1.48	0.256	95.75	< 0.001	72.71	0.128	20	27
Agency attribution (13 tested moderators)	Interaction medium	7.09	**0.026**	137.70	< 0.001	91.51	4.331	11	18
	Computer-mediated (*k* = 7, *m* = 12): -0.367 [-0.79, 0.05]; *t* = -2.14, *p* = 0.07<br>Face-to-face (*k* = 4, *m* = 6): -1.254 [-2.33, -0.18]; *t* = -3.72, *p* = 0.034								
	Study setting	2.64	0.139	206.24	< 0.001	93.84	0.990	11	18
	Publication year	0.19	0.677	226.14	< 0.001	95.10	0.245	11	18
	Sample WEIRD	2.43	0.158	146.47	< 0.001	93.26	0.817	10	14
	Main age	2.88	0.124	170.27	< 0.001	93.65	0.872	11	18
	Percentage female	0.40	0.543	222.33	< 0.001	94.95	0.233	11	18
	Agent operationalisation	1.57	0.266	206.28	< 0.001	94.16	0.424	11	18
	Agent form	0.47	0.512	228.91	< 0.001	94.93	0.447	11	18
	Agent embodiment	0.18	0.680	229.43	< 0.001	95.08	0.415	11	18
	Appearance difference	1.77	0.217	188.34	< 0.001	94.25	0.808	11	18
	Interaction realism	3.11	0.112	213.60	< 0.001	93.66	0.980	11	18
	Interaction nature	1.35	0.262	205.85	< 0.001	94.45	0.543	11	18
	Power symmetry	1.90	0.201	207.86	< 0.001	94.19	0.793	11	18
Responsibility attribution (6 tested moderators)	Study setting	11.34	**0.007**	54.36	< 0.001	71.08	4.271	12	18
	Lab experiment (*k* = 3, *m* = 7): -0.188 [-0.59, 0.21]; *t* = -2.02, *p* = 0.181<br>Online experiment (*k* = 9, *m* = 11): -0.630 [-0.85, -0.41]; *t* = -6.51, *p* < 0.001								
	Interaction realism	11.34	**0.007**	54.36	< 0.001	71.08	4.271	12	18
	Real-time (*k* = 3, *m* = 7): -0.188 [-0.59, 0.21]; *t* = -2.02, *p* = 0.181<br>Hypothetical (*k* = 9, *m* = 11): -0.630 [-0.85, -0.41]; *t* = -6.51, *p* < 0.001								
	Appearance difference	6.11	**0.033**	59.27	< 0.001	74.34	2.224	12	18
	Publication year	6.56	**0.028**	61.66	< 0.001	75.33	0.894	12	18
	Percentage female	1.55	0.242	87.52	< 0.001	81.08	0.207	12	18
	Interaction medium	1.37	0.269	90.13	< 0.001	81.54	0.415	12	18
**Theme 3: Trust in interaction partners**									
Behavioural trust (16 tested moderators)	Study setting	0.73	0.406	46.78	0.002	62.00	0.195	18	24
	Study design	0.00	0.972	47.74	0.001	63.82	0.150	18	24
	Publication type	1.25	0.280	46.13	0.002	61.30	0.301	18	24
	Publication year	3.38	0.085	40.66	0.009	56.75	0.410	18	24
	Main age	0.27	0.612	36.50	0.019	51.58	0.072	17	23
	Percentage female	0.13	0.724	36.94	0.012	55.40	0.072	16	22
	Human partner type	0.79	0.471	44.14	0.002	63.04	0.071	18	24
	Agent form	0.25	0.621	44.94	0.003	62.21	0.178	18	24
	Agent embodiment	0.00	0.955	47.68	0.001	63.51	0.155	18	24
	Appearance difference	0.00	0.955	47.68	0.001	63.51	0.155	18	24
	Voice difference	0.82	0.380	46.77	0.002	62.91	0.211	18	24
	Interaction medium	0.34	0.567	46.09	0.002	62.74	0.225	18	24

Responses	Moderators	*F*	*F_p*	*QE*	*QE_p*	I^2 (%)	BF_{10}	*k*	*m*
	Interaction nature	0.19	0.825	46.65	0.001	65.22	0.036	18	24
	Power symmetry	0.76	0.395	46.34	0.002	60.92	0.216	18	24
	Interaction task	0.09	0.767	40.85	0.003	63.76	0.203	16	21
	Response dimension	2.18	0.162	33.58	0.014	56.76	0.459	16	20
Subjective trust (21 tested moderators)	Measurement timing	4.59	**0.043**	100.39	< 0.001	73.31	0.817	25	35
	Study setting	0.55	0.468	115.03	< 0.001	77.78	0.164	25	35
	Study design	0.33	0.573	112.21	< 0.001	77.35	0.172	25	35
	Publication type	1.10	0.304	110.40	< 0.001	76.68	0.261	25	35
	Publication year	0.18	0.678	115.22	< 0.001	77.88	0.072	25	35
	Sample continent	0.89	0.359	102.18	< 0.001	75.54	0.244	19	27
	Sample WEIRD	1.04	0.320	106.58	< 0.001	78.16	0.255	22	30
	Main age	2.56	0.125	93.94	< 0.001	75.60	0.196	23	32
	Percentage female	3.95	0.060	100.45	< 0.001	73.75	0.354	23	32
	Human partner type	0.76	0.531	111.81	< 0.001	78.58	0.014	25	35
	Agent operationalisation	1.42	0.246	106.85	< 0.001	77.17	0.321	23	33
	Agent form	0.17	0.682	114.39	< 0.001	77.70	0.155	25	35
	Agent embodiment	0.30	0.589	115.39	< 0.001	77.63	0.140	25	35
	Appearance difference	0.76	0.392	112.23	< 0.001	77.22	0.206	25	35
	Voice appearance	0.05	0.816	115.19	< 0.001	77.53	0.147	25	35
	Interaction realism	1.95	0.176	113.35	< 0.001	76.98	0.407	25	35
	Interaction medium	0.22	0.645	115.23	< 0.001	77.85	0.205	25	35
	Interaction nature	0.08	0.777	97.55	< 0.001	79.22	0.147	23	29
	Power symmetry	0.06	0.810	115.29	< 0.001	77.77	0.160	25	35
	Interaction task	2.08	0.154	74.40	< 0.001	75.51	0.204	21	27
	Response dimension	0.38	0.545	104.36	< 0.001	78.02	0.188	23	32
**Theme 4: Social alignment with interaction partners**									
Social alignment (24 tested moderators)	Percentage female	5.63	**0.028**	45.06	0.097	24.60	0.543	22	36
	Main age	5.94	**0.027**	33.28	0.311	11.66	0.372	18	32
	Study setting	0.00	0.963	52.00	0.025	36.48	0.105	22	36
	Study design	0.58	0.456	51.93	0.025	34.28	0.203	22	36
	Publication year	0.66	0.425	52.80	0.021	39.17	0.058	22	36
	Sample continent	0.26	0.773	28.40	0.040	49.98	0.033	17	20
	Sample WEIRD	0.02	0.890	30.06	0.037	48.30	0.158	17	20
	Human partner type	3.14	0.051	40.45	0.145	23.06	0.094	22	36
	Agent operationalisation	1.45	0.260	46.00	0.066	25.36	0.045	22	36
	Agent form	0.50	0.487	52.32	0.023	35.77	0.127	22	36
	Agent embodiment	0.00	0.974	52.89	0.021	36.29	0.111	22	36
	Robot appearance	0.01	0.919	30.21	0.049	37.40	0.167	10	21
	Appearance difference	0.02	0.886	52.85	0.021	36.21	0.110	22	36
	Voice difference	0.04	0.836	51.61	0.027	35.63	0.114	22	36
	Interaction realism	0.02	0.880	52.71	0.021	36.46	0.124	22	36
	Interaction flow	0.26	0.617	52.38	0.023	35.92	0.146	22	36
	Interaction medium	0.00	0.974	39.69	0.031	37.20	0.120	15	27
	Interaction structure	4.01	0.059	47.01	0.068	25.39	0.708	22	36
	Interaction nature	3.44	0.079	47.28	0.051	29.96	0.774	21	35
	Power symmetry	0.01	0.919	52.88	0.021	35.92	0.112	22	36
	Interaction task	0.68	0.578	31.32	0.037	48.80	0.008	20	23
	Response domain	1.37	0.256	52.13	0.024	36.87	0.196	22	36
	Measurement timing	0.10	0.751	52.89	0.020	37.17	0.112	22	36
	Response dimension	3.32	0.061	37.29	0.202	20.99	0.123	20	34

Responses	Moderators	*F*	*F_p*	*QE*	*QE_p*	I^2 (%)	BF_{10}	*k*	*m*
**Theme 5: Personal agency and task performance**									
Perceived self-agency	*non-significant heterogeneity (Q = 18.51, p = 0.139); insufficient studies (k = 9, m = 14)*								
Self-disclosure	*non-significant heterogeneity (Q = 28.03, p = 0.109); insufficient studies (k = 9, m = 21)*								
Strategic economic behaviour	*non-significant heterogeneity (Q = 32.82, p = 0.084)*								
Objective task performance (21 tested moderators)	Study setting	0.06	0.815	69.48	< 0.001	48.09	0.155	23	38
	Study design	2.21	0.152	64.82	0.002	43.75	0.352	23	38
	Publication type	0.46	0.506	68.06	0.001	46.60	0.152	23	38
	Publication year	4.00	0.058	63.87	0.003	42.26	0.393	23	38
	Sample continent	0.13	0.879	67.88	< 0.001	52.31	0.025	21	36
	Sample WEIRD	0.22	0.640	68.70	< 0.001	49.05	0.173	22	37
	Main age	0.15	0.704	47.07	0.032	38.47	0.061	20	33
	Percentage female	0.73	0.404	68.56	< 0.001	48.32	0.082	22	37
	Human partner type	0.11	0.749	69.29	< 0.001	49.52	0.146	22	37
	Agent operationalisation	0.30	0.591	68.91	< 0.001	48.45	0.175	22	37
	Agent form	0.92	0.345	52.76	0.021	38.62	0.182	22	36
	Agent embodiment	0.50	0.486	52.52	0.022	38.01	0.174	22	36
	Robot appearance	0.01	0.934	33.31	0.015	47.73	0.174	12	20
	Appearance difference	1.87	0.186	68.53	< 0.001	46.96	0.229	23	38
	Voice difference	0.17	0.681	70.60	< 0.001	48.65	0.126	23	38
	Interaction flow	0.06	0.817	70.51	< 0.001	48.07	0.139	23	38
	Interaction medium	0.14	0.715	48.50	0.040	35.81	0.121	21	35
	Interaction structure	0.14	0.716	70.45	< 0.001	47.88	0.221	23	38
	Interaction nature	1.28	0.302	49.57	0.032	37.90	0.064	22	36
	Power symmetry	0.52	0.479	69.54	< 0.001	47.96	0.155	23	38
	Interaction task	1.41	0.275	40.47	0.119	29.22	0.018	21	35
**Theme 6: Interaction experiences**									
Perceived partner relational qualities (17 tested moderators)	Agent operationalisation	11.37	**0.001**	119.90	< 0.001	80.45	71.227	17	25
	Autonomous (*k* = 4, *m* = 4): 0.024 [-0.17, 0.22]; *t* = 0.39, *p* = 0.726<br>Wizard-of-Oz (*k* = 8, *m* = 15): 0.123 [-0.14, 0.39]; *t* = 1.10, *p* = 0.308<br>Vignette-described (*k* = 5, *m* = 6): -0.709 [-1.20, -0.22]; *t* = -4.01, *p* = 0.016								
	Human partner type	10.75	**0.002**	119.33	< 0.001	81.69	45.469	16	24
	Research team member (*k* = 8, *m* = 15): 0.118 [-0.13, 0.36]; *t* = 1.15, *p* = 0.289<br>Pseudo-human (*k* = 3, *m* = 3): -0.004 [-0.32, 0.31]; *t* = -0.06, *p* = 0.958<br>Vignette-described partner (*k* = 5, *m* = 6): -0.709 [-1.20, -0.22]; *t* = -4.01, *p* = 0.016								
	Interaction realism	23.50	**< 0.001**	120.48	< 0.001	79.47	298.645	17	25
	Real-time (*k* = 12, *m* = 19): 0.081 [-0.06, 0.23]; *t* = 1.23, *p* = 0.243<br>Hypothetical (*k* = 5, *m* = 6): -0.709 [-1.20, -0.22]; *t* = -4.01; *p* = 0.016								
	Response dimension	6.78	**0.006**	58.17	< 0.001	82.88	3.334	16	24
	Perceived rapport (*k* = 3, *m* = 10): 0.171 [-0.88, 1.23]; *t* = 0.70, *p* = 0.557<br>Perceived interactivity (*k* = 6, *m* = 6): -0.003 [-0.15, 0.14]; *t* = -0.05, *p* = 0.960<br>Perceived empathy (*k* = 5, *m* = 5): -0.218 [-0.82, 0.39]; *t* = -1.00, *p* = 0.374<br>Perceived customer orientation (*k* = 3, *m* = 3): -0.873 [-1.31, -0.44]; *t* = -8.59, *p* = 0.013								
	Study setting	6.30	**0.024**	134.19	< 0.001	88.71	2.913	17	25
	Main age	6.86	**0.020**	122.71	< 0.001	89.02	2.438	16	24
	Interaction task	6.04	**0.034**	105.81	< 0.001	89.42	2.849	12	16
	Study design	0.72	0.409	154.23	< 0.001	91.84	0.343	17	25
	Publication type	2.13	0.165	150.17	< 0.001	91.09	0.674	17	25
	Publication year	1.89	0.190	146.31	< 0.001	91.05	0.346	17	25

Responses	Moderators	F	F_p	QE	QE_p	I^2 (%)	BF_{10}	k	m
	Percentage female	3.00	0.104	121.88	< 0.001	90.37	0.463	17	25
	Agent form	0.58	0.456	155.48	< 0.001	91.87	0.288	17	25
	Agent embodiment	0.03	0.856	154.38	< 0.001	93.36	0.306	16	21
	Appearance difference	0.28	0.602	156.21	< 0.001	92.01	0.263	17	25
	Voice difference	2.13	0.165	150.17	< 0.001	91.09	0.674	17	25
	Interaction medium	0.98	0.339	149.38	< 0.001	93.44	0.370	15	21
	Interaction structure	0.90	0.357	154.35	< 0.001	91.67	0.383	17	25
Affective valence (9 tested moderators)	Study setting	2.98	0.123	60.75	< 0.001	83.25	1.018	10	14
	Study design	1.24	0.298	78.90	< 0.001	86.12	0.489	10	14
	Publication year	0.11	0.752	82.00	< 0.001	87.54	0.156	10	14
	Main age	0.21	0.663	84.44	< 0.001	87.55	0.180	10	14
	Percentage female	0.08	0.780	81.77	< 0.001	87.58	0.158	10	14
	Agent form	0.22	0.652	83.33	< 0.001	87.52	0.331	10	14
	Agent embodiment	2.98	0.123	60.75	< 0.001	83.25	1.026	10	14
	Appearance difference	0.97	0.354	74.41	< 0.001	86.23	0.483	10	14
	Power symmetry	0.01	0.922	84.30	< 0.001	87.77	0.302	10	14
Affective arousal	*non-significant heterogeneity (Q = 17.81, p = 0.058); insufficient studies (k = 6, m = 11)*								
Interaction satisfaction (16 tested moderators)	Interaction nature	40.31	**< 0.001**	185.20	< 0.001	92.13	15137.576	18	26
	Cooperative (k = 15, m = 20): -0.081 [-0.29, 0.13]; t = -0.83, p = 0.421<br>Oppositional (k = 6, m = 6): 0.493 [0.23, 0.76]; t = 4.77, p = 0.005								
	Agent operationalisation	5.51	**0.032**	239.26	< 0.001	93.94	2.759	18	26
	Study setting	2.14	0.164	241.22	< 0.001	94.70	0.733	17	25
	Publication type	2.95	0.104	255.76	< 0.001	94.26	0.881	19	27
	Publication year	0.03	0.871	277.37	< 0.001	95.01	0.100	19	27
	Sample continent	1.87	0.193	136.68	< 0.001	93.59	0.293	16	22
	Sample WEIRD	1.97	0.182	140.50	< 0.001	94.15	0.560	16	22
	Main age	3.07	0.105	216.71	< 0.001	94.97	0.579	14	22
	Percentage female	1.23	0.284	195.49	< 0.001	94.58	0.190	19	27
	Human partner type	4.22	0.056	242.75	< 0.001	93.83	1.438	19	27
	Agent form	1.74	0.204	215.11	< 0.001	94.52	0.424	19	27
	Agent embodiment	0.85	0.370	218.16	< 0.001	94.70	0.300	19	27
	Appearance difference	1.74	0.204	215.11	< 0.001	94.52	0.460	19	27
	Voice difference	1.01	0.329	247.77	< 0.001	94.69	0.405	19	27
	Interaction realism	4.22	0.056	242.75	< 0.001	93.83	1.438	19	27
	Interaction medium	1.74	0.204	215.11	< 0.001	94.52	0.460	19	27
Future interaction intention	*insufficient studies (k = 6, m = 7)*								
Perceived interaction naturalness	*insufficient studies (k = 5, m = 5)*								
Perceived interaction enjoyment	*insufficient studies (k = 7, m = 10)*								
Subjective workload	*insufficient studies (k = 5, m = 14)*								
Subjective task engagement (11 tested moderators)	Study design	4.55	0.065	13.74	0.132	35.64	1.201	10	11
	Publication type	2.66	0.142	17.70	0.039	55.35	0.735	10	11
	Publication year	1.17	0.311	18.24	0.032	59.10	0.205	10	11
	Main age	1.54	0.249	19.06	0.025	58.76	0.257	10	11
	Percentage female	1.91	0.204	17.49	0.042	57.96	0.310	10	11
	Human partner type	1.56	0.247	17.81	0.037	56.84	0.499	10	11
	Agent operationalisation	0.49	0.503	19.45	0.022	61.03	0.339	10	11

Responses	Moderators	*F*	*F_p*	*QE*	*QE_p*	I^2 (%)	BF_{10}	*k*	*m*
	Agent form	1.56	0.247	17.81	0.037	56.84	0.499	10	11
	Appearance difference	1.56	0.247	17.81	0.037	56.84	0.509	10	11
	Voice difference	0.84	0.387	18.57	0.029	58.32	0.369	10	11
	Power symmetry	2.29	0.169	16.50	0.057	51.02	0.643	10	11

Note: Meta-regressions were conducted for response types with significant heterogeneity in effect sizes and at least ten studies available; potential moderators were evaluated individually. For categorical moderators with multiple levels, only levels represented by at least three studies were included[141]. BF_{10} is presented to provide complementary Bayesian evidence for H_1 (i.e., presence of a moderating effect) over H_0 (i.e., absence of a moderating effect); values marked in blue ($BF_{10} < 1/3$) indicate at least substantial evidence supporting H_0, values in orange ($BF_{10} > 3$) indicate at least substantial evidence supporting H_1, and values in grey ($1/3 \leq BF_{10} \leq 3$) indicate ambiguous evidence. *F*-value is from random-effects meta-regression using REML with Hartung-Knapp adjustment. *QE* is Cochrane's *Q*-statistic for testing residual heterogeneity. I^2 is the proportion of residual variance attributable to true heterogeneity after accounting for the moderator. *k* is the number of studies including in meta-regression; *m* is the number of effect sizes included. Furthermore, subgroup analyses were carried out only when a significant moderating effect identified by frequentist analysis was also supported by at least substantial Bayesian evidence.

Supplementary Table 10. Egger Sandwich tests

**Responses**	**Egger Sandwich test results**
**Theme 1: Prosociality and morality**	
Prosocial behaviour	*insufficient studies (k = 9, m = 10)*
Moral engagement	*insufficient studies (k = 8, m = 14)*
**Theme 2: Social perceptions of interaction partners**	
Perceived social presence	*insufficient studies (k = 9, m = 20)*
Perceived likeability	$b = 0.136$, $t(12.58) = 0.12$, $p = 0.903$
Perceived competence	$b = 0.615$, $t(9.97) = 0.75$, $p = 0.469$
Agency attribution	$b = -4.756$, $t(1.35) = -0.49$, $p = 0.692$
Responsibility attribution	$b = -4.395$, $t(3.59) = -1.03$, $p = 0.367$
**Theme 3: Trust in interaction partners**	
Behavioural trust	$b = 1.232$, $t(4.87) = 0.92$, $p = 0.403$
Subjective trust	$b = 0.480$, $t(2.47) = 0.88$, $p = 0.457$
**Theme 4: Social alignment with interaction partners**	
Social alignment	$b = 1.100$, $t(5.02) = 3.45$, $p = 0.018$
**Theme 5: Personal agency and task performance**	
Perceived self-agency	*insufficient studies (k = 9, m = 14)*
Self-disclosure	*insufficient studies (k = 9, m = 21)*
Strategic economic behaviour	$b = -0.416$, $t(6.67) = -0.43$, $p = 0.679$
Objective task performance	$b = 1.862$, $t(7.90) = 2.48$, $p = 0.038$
**Theme 6: Interaction experiences**	
Perceived partner relational qualities	$b = 2.029$, $t(5.49) = 1.32$, $p = 0.239$
Affective valence	$b = -1.904$, $t(2.09) = -1.35$, $p = 0.306$
Affective arousal	*insufficient studies (k = 6, m = 11)*
Interaction satisfaction	$b = 0.999$, $t(5.26) = 0.64$, $p = 0.550$
Future interaction intention	*insufficient studies (k = 6, m = 7)*
Perceived interaction naturalness	*insufficient studies (k = 5, m = 5)*
Perceived interaction enjoyment	*insufficient studies (k = 7, m = 10)*
Subjective workload	*insufficient studies (k = 5, m = 14)*
Subjective task engagement	$b = 1.296$, $t(3.88) = 1.04$, $p = 0.358$

Supplementary Table 11. Research quality assessment of individual studies

Research quality varied greatly across the studies; on average, study design rigour scored 11.18 (*SD* = 1.88, range = 7 – 18; max. 20), data & reporting rigour 9.39 (*SD* = 1.79, range = 4 – 12; max. 12), and broad research integrity 7.67 (*SD* = 3.04, range = 2 – 14; max. 14), indicating an overall moderate level of research quality.

No.	Study	Study design rigour	% of max. 20	Data & reporting rigour	% of max. 12	Broad research integrity	% of max. 14
1	(Alarcon, 2023) S1	10	50.00%	10	83.33%	10	71.43%
2	(Appel, 2012) S1	12	60.00%	10	83.33%	7	50.00%
3	(Bailenson, 2003) S1	12	60.00%	9	75.00%	4	28.57%
4	(Bailenson, 2003) S2	12	60.00%	9	75.00%	4	28.57%
5	(Banks, 2020) S1	13	65.00%	12	100.00%	14	100.00%
6	(Belanche, 2020) S1	10	50.00%	10	83.33%	6	42.86%
7	(Belanche, 2020) S2	9	45.00%	10	83.33%	6	42.86%
8	(Bergmann, 2015) S1	12	60.00%	12	100.00%	7	50.00%
9	(Bergmann, 2015) S2	12	60.00%	11	91.67%	7	50.00%
10	(Bouquet, 2024) S2	13	65.00%	11	91.67%	13	92.86%
11	(Bowman, 2019) S1	13	65.00%	11	91.67%	5	35.71%
12	(Bunlon, 2018) S2	15	75.00%	10	83.33%	10	71.43%
13	(Čaić, 2020) S1	10	50.00%	10	83.33%	8	57.14%
14	(Caruana, 2019) S1	11	55.00%	9	75.00%	6	42.86%
15	(Chen, 2023) S1	11	55.00%	12	100.00%	12	85.71%
16	(Chen, 2023) S2	11	55.00%	12	100.00%	12	85.71%
17	(Chen, 2023) S3	11	55.00%	11	91.67%	12	85.71%
18	(Choi, 2019) S1	9	45.00%	8	66.67%	4	28.57%
19	(Cominelli, 2021) S1	13	65.00%	7	58.33%	12	85.71%
20	(Cowan, 2015) S1	12	60.00%	10	83.33%	8	57.14%
21	(Cowan, 2015) S2	12	60.00%	10	83.33%	8	57.14%
22	(De Kleijn, 2019) S1	10	50.00%	9	75.00%	8	57.14%
23	(De Melo, 2019) S1a	12	60.00%	11	91.67%	13	92.86%
24	(De Melo, 2019) S1b	12	60.00%	11	91.67%	13	92.86%
25	(De Visser, 2016) S1	9	45.00%	10	83.33%	5	35.71%
26	(De Visser, 2016) S2	9	45.00%	8	66.67%	5	35.71%
27	(De Visser, 2016) S3	9	45.00%	8	66.67%	5	35.71%
28	(Desideri, 2019) S1	11	55.00%	12	100.00%	10	71.43%
29	(Edwards, 2019) S1	8	40.00%	11	91.67%	5	35.71%
30	(Edwards, 2021) S1	13	65.00%	12	100.00%	8	57.14%
31	(Frank, 2023) S1	12	60.00%	12	100.00%	6	42.86%
32	(Frank, 2023) S2	10	50.00%	11	91.67%	6	42.86%
33	(Frank, 2023) S3	10	50.00%	11	91.67%	6	42.86%
34	(Garvey, 2023) S2	12	60.00%	10	83.33%	5	35.71%
35	(Giroux, 2022) S1	8	40.00%	12	100.00%	8	57.14%
36	(Giroux, 2022) S2	9	45.00%	11	91.67%	8	57.14%
37	(Go, 2019) S1	12	60.00%	10	83.33%	6	42.86%

No.	Study	Study design rigour	% of max. 20	Data & reporting rigour	% of max. 12	Broad research integrity	% of max. 14
38	(Gonzalez-Billandon, 2019) S1	9	45.00%	7	58.33%	12	85.71%
39	(Gratch, 2016; Mozgai, 2017) S1	9	45.00%	7	58.33%	3	21.43%
40	(Guadagno, 2007) S2	12	60.00%	10	83.33%	6	42.86%
41	(Guadagno, 2011) S1	10	50.00%	9	75.00%	7	50.00%
42	(Haring, 2019) S1	11	55.00%	9	75.00%	10	71.43%
43	(Harriott, 2013) S2	9	45.00%	10	83.33%	2	14.29%
44	(Hertz, 2016) S1	13	65.00%	9	75.00%	10	71.43%
45	(Hinds, 2004) S1	11	55.00%	9	75.00%	5	35.71%
46	(Ho, 2018) S1	15	75.00%	9	75.00%	8	57.14%
47	(Hoffman, 2015) S1	12	60.00%	5	41.67%	7	50.00%
48	(Holthöwer, 2023) S3	11	55.00%	8	66.67%	8	57.14%
49	(Hoorn, 2024) S1	12	60.00%	9	75.00%	11	78.57%
50	(Horstmann, 2021) S1	13	65.00%	10	83.33%	14	100.00%
51	(Huang, 2015) S1	7	35.00%	4	33.33%	4	28.57%
52	(Jerčić, 2018) S1	12	60.00%	8	66.67%	10	71.43%
53	(Jois, 2021) S1	11	55.00%	8	66.67%	9	64.29%
54	(Kahn, 2015) S1	11	55.00%	8	66.67%	4	28.57%
55	(Karpus, 2021) S1	8	40.00%	8	66.67%	14	100.00%
56	(Karpus, 2021) S2	9	45.00%	8	66.67%	14	100.00%
57	(Karpus, 2021) S3	9	45.00%	8	66.67%	14	100.00%
58	(Karpus, 2021) S4	9	45.00%	8	66.67%	14	100.00%
59	(Karpus, 2021) S9	10	50.00%	8	66.67%	14	100.00%
60	(Kawai, 2023) S1	9	45.00%	8	66.67%	12	85.71%
61	(Khalighinejad, 2016) S1	12	60.00%	12	100.00%	9	64.29%
62	(Kiilavuori, 2022) S1	18	90.00%	10	83.33%	9	64.29%
63	(Kiilavuori, 2021) S1	14	70.00%	10	83.33%	11	78.57%
64	(Kim, 2018) S1/2	10	50.00%	10	83.33%	8	57.14%
65	(Kim, 2020) S2	10	50.00%	12	100.00%	7	50.00%
66	(Kim, 2020) S5	14	70.00%	12	100.00%	7	50.00%
67	(Kim, 2022) S2	13	65.00%	10	83.33%	6	42.86%
68	(Kim, 2023) S1	11	55.00%	10	83.33%	6	42.86%
69	(Kim, 2023) S2	13	65.00%	10	83.33%	6	42.86%
70	(Kim, 2023) S3	11	55.00%	8	66.67%	6	42.86%
71	(Krach, 2008) S1	10	50.00%	5	41.67%	11	78.57%
72	(Kulms, 2019) S1	9	45.00%	9	75.00%	3	21.43%
73	(Leiño Calleja, 2023) S1	9	45.00%	10	83.33%	5	35.71%
74	(Leiño Calleja, 2023) S2	10	50.00%	10	83.33%	5	35.71%
75	(Leiño Calleja, 2023) S3b	10	50.00%	10	83.33%	5	35.71%
76	(Leo, 2020) S1a	14	70.00%	8	66.67%	10	71.43%
77	(Leo, 2020) S2	15	75.00%	10	83.33%	10	71.43%
78	(Liao, 2024) S2	11	55.00%	10	83.33%	6	42.86%
79	(Liao, 2023) S1	15	75.00%	12	100.00%	8	57.14%
80	(Liao, 2023) S2	15	75.00%	12	100.00%	8	57.14%
81	(Liu, 2024) S1a	15	75.00%	11	91.67%	12	85.71%

No.	Study	Study design rigour	% of max. 20	Data & reporting rigour	% of max. 12	Broad research integrity	% of max. 14
82	(Liu, 2024) S1b	15	75.00%	11	91.67%	12	85.71%
83	(Lin, 2022) S1	11	55.00%	7	58.33%	7	50.00%
84	(Liu, 2020) S1	11	55.00%	9	75.00%	7	50.00%
85	(Maehigashi, 2022) S1	12	60.00%	9	75.00%	4	28.57%
86	(Maggioni, 2023) S1	11	55.00%	11	91.67%	8	57.14%
87	(Mell, 2017) S1	9	45.00%	5	41.67%	3	21.43%
88	(Meng, 2021) S1	12	60.00%	11	91.67%	7	50.00%
89	(Merkle, 2019) S1	8	40.00%	10	83.33%	6	42.86%
90	(Merritt, 2011) S1	10	50.00%	8	66.67%	5	35.71%
91	(Mirbabaie, 2021) S1	12	60.00%	9	75.00%	5	35.71%
92	(Naito, 2023) S1	13	65.00%	9	75.00%	10	71.43%
93	(Naito, 2023) S2	13	65.00%	9	75.00%	10	71.43%
94	(Naito, 2023) S3	13	65.00%	9	75.00%	10	71.43%
95	(Ng, 2023) S1	11	55.00%	11	91.67%	8	57.14%
96	(Nishio, 2012) S1	10	50.00%	4	33.33%	5	35.71%
97	(Numata, 2020) S1	13	65.00%	12	100.00%	11	78.57%
98	(Ossadnik, 2023) S1	11	55.00%	10	83.33%	6	42.86%
99	(Palanica, 2019) S1	10	50.00%	9	75.00%	10	71.43%
100	(Pavone, 2023) S1	11	55.00%	11	91.67%	8	57.14%
101	(Pickard, 2020) S1	11	55.00%	10	83.33%	8	57.14%
102	(Plaks, 2022) S1	14	70.00%	9	75.00%	10	71.43%
103	(Poinsot, 2022) S1	9	45.00%	10	83.33%	9	64.29%
104	(Riether, 2012) S1	13	65.00%	10	83.33%	5	35.71%
105	(Roozen, 2023) S1	9	45.00%	10	83.33%	8	57.14%
106	(Russo, 2021) S1	14	70.00%	12	100.00%	6	42.86%
107	(Ryoo, 2024) S1A	11	55.00%	10	83.33%	6	42.86%
108	(Ryoo, 2024)S1B	11	55.00%	10	83.33%	6	42.86%
109	(Ryoo, 2024) S2	9	45.00%	11	91.67%	6	42.86%
110	(Ryoo, 2024) S3	11	55.00%	9	75.00%	6	42.86%
111	(Sandoval, 2016) S1	10	50.00%	7	58.33%	7	50.00%
112	(Sahaï, 2023) S1	16	80.00%	12	100.00%	11	78.57%
113	(Sciutti, 2013) S1	9	45.00%	8	66.67%	5	35.71%
114	(Shen, 2022) S1	10	50.00%	10	83.33%	6	42.86%
115	(Shin, 2023) S1	12	60.00%	9	75.00%	8	57.14%
116	(Sundar, 2019) S1	11	55.00%	12	100.00%	4	28.57%
117	(Singh, 2021) S1	10	50.00%	6	50.00%	8	57.14%
118	(Smyk, 2018) S1	11	55.00%	10	83.33%	11	78.57%
119	(Söderlund, 2020) S1	10	50.00%	8	66.67%	3	21.43%
120	(Stock, 2018) S1	14	70.00%	9	75.00%	3	21.43%
121	(Stock-Homburg, 2020) S1	10	50.00%	7	58.33%	3	21.43%
122	(Tatsukawa, 2018) S1	10	50.00%	8	66.67%	11	78.57%
123	(Takahashi, 2014) S1	11	55.00%	8	66.67%	9	64.29%
124	(Terada, 2017) S2	11	55.00%	8	66.67%	12	85.71%
125	(Torta, 2013) S1	9	45.00%	7	58.33%	2	14.29%

No.	Study	Study design rigour	% of max. 20	Data & reporting rigour	% of max. 12	Broad research integrity	% of max. 14
126	(Tsfasman, 2021) S1	11	55.00%	6	50.00%	6	42.86%
127	(Tu, 2023) S1	12	60.00%	9	75.00%	3	21.43%
128	(Tuvo, 2022) S1	11	55.00%	5	41.67%	7	50.00%
129	(Von Der Pütten, 2010) S1	10	50.00%	10	83.33%	5	35.71%
130	(Walliser, 2015) S1	10	50.00%	8	66.67%	7	50.00%
131	(Wang, 2023) S1	12	60.00%	11	91.67%	10	71.43%
132	(Wang, 2023) S2	12	60.00%	9	75.00%	10	71.43%
133	(Xiao, 2023) S1	12	60.00%	9	75.00%	8	57.14%
134	(Xiao, 2023) S2	12	60.00%	10	83.33%	8	57.14%
135	(Xiao, 2021) S1	11	55.00%	9	75.00%	11	78.57%
136	(Xu, 2018a; Xu, 2018b) S1	7.5	37.50%	5.5	45.83%	6.5	46.43%
137	(Yu, 2024) S1	11	55.00%	11	91.67%	4	28.57%
138	(Yu, 2024) S2	11	55.00%	11	91.67%	4	28.57%
139	(Yu, 2024) S3	11	55.00%	11	91.67%	4	28.57%
140	(Yu, 2024) S4B	12	60.00%	10	83.33%	4	28.57%
141	(Yu, 2024) S5	11	55.00%	9	75.00%	4	28.57%
142	(Yu, 2024) S1	10	50.00%	9	75.00%	10	71.43%
143	(Zhang, 2023) S1	8	40.00%	5	41.67%	12	85.71%
144	(Zhou, 2022) S2	14	70.00%	10	83.33%	4	28.57%
145	(Zhou, 2022) S3	12	60.00%	10	83.33%	4	28.57%
146	(Zonca, 2023) S1	10	50.00%	9	75.00%	11	78.57%

Note. This table presents three quality metrics for included studies, alongside each metric's percentage of its maximum possible score. Specifically, "study design rigour" reflects how thoughtfully each study was planned and designed, assessed through quality items on objectives and preregistrations, participants, and study design; "data & reporting rigour" reflects how rigorously study data were handled, assessed through items on data collection, analysis, and results; "broad research integrity" captured broader practices that support reproducibility and trustworthiness, assessed through items on discussion, ethics, and open science. Detailed item-level ratings for each study are available in the OSF (https://doi.org/10.17605/OSF.IO/4X26R). For (Gratch, 2016; Mozgai, 2017) S1, it comprised two articles[51,52] that reported the same underlying study while analysing different human responses; thus, we computed the mean rating for each of the three quality metrics across those two articles. Similarly, (Xu, 2018a; Xu, 2018b) S1 comprised two articles[133,134] on the same study, and we derived this study's quality metrics by averaging the ratings from both articles.

Supplementary Table 12. Impact of research quality

Research quality of individual studies was assessed via a tailored checklist (Supplementary Table 5). Three quality metrics, i.e., study design rigour, data & reporting rigour, and broad research integrity, were calculated for each study (Supplementary Table 11). Univariate meta-regressions showed that three metrics did not significantly moderate partner effects across most response types, expect for study design rigour for social alignment and data & reporting rigour for interaction satisfaction (ps = 0.036 and 0.024; highlighted in orange below); however, both had ambiguous Bayesian evidence (BF_{10} = 0.452 and 1.590). A few non-significant moderations by quality metrics also had ambiguous Bayesian evidence (6/37; BF_{10} = 0.381 – 0.708; highlighted in orange below): Study design rigour for perceived likeability, agency attribution, and perceived partner relational qualities; data & reporting rigour for behavioural trust; broad research integrity for perceived competence and perceived partner relational qualities. This ambiguity presumably stems from the relatively small number of studies available for robust meta-regression. Overall, research quality had no systematic impact on our results, though modest influences of specific quality metrics cannot be ruled out.

**Responses**	**Meta-regression results** (Q1: study design rigour; Q2: data & reporting rigour; Q3: broad research integrity)
**Theme 1: Prosociality and morality**	
Prosocial behaviour	*insufficient studies (k = 9, m = 10)*
Moral engagement	*insufficient studies (k = 8, m = 14)*
**Theme 2: Social perceptions of interaction partners**	
Perceived social presence	*insufficient studies (k = 9, m = 20)*
Perceived likeability	**Q1:** $F(1, 26) = 2.49, p = 0.127$ ($BF_{10} = 0.381$)<br>**Q2:** $F(1, 26) = 0.26, p = 0.616$ ($BF_{10} = 0.120$)<br>**Q3:** $F(1, 26) = 1.77, p = 0.195$ ($BF_{10} = 0.250$)
Perceived competence	**Q1:** $F(1, 21) = 0.68, p = 0.418$ ($BF_{10} = 0.115$)<br>**Q2:** $F(1, 21) = 0.17, p = 0.687$ ($BF_{10} = 0.095$)<br>**Q3:** $F(1, 21) = 4.16, p = 0.054$ ($BF_{10} = 0.634$)
Agency attribution	**Q1:** $F(1, 9) = 2.83, p = 0.127$ ($BF_{10} = 0.697$)<br>**Q2:** $F(1, 9) = 0.00, p = 0.968$ ($BF_{10} = 0.196$)<br>**Q3:** $F(1, 9) = 0.00, p = 0.980$ ($BF_{10} = 0.227$)
Responsibility attribution	**Q1:** $F(1, 10) = 1.92, p = 0.196$ ($BF_{10} = 0.266$)<br>**Q2:** $F(1, 10) = 0.05, p = 0.825$ ($BF_{10} = 0.119$)<br>**Q3:** $F(1, 10) = 0.01, p = 0.912$ ($BF_{10} = 0.114$)
**Theme 3: Trust in interaction partners**	
Behavioural trust	**Q1:** $F(1, 16) = 0.08, p = 0.783$ ($BF_{10} = 0.066$)<br>**Q2:** $F(1, 16) = 3.70, p = 0.072$ ($BF_{10} = 0.420$)<br>**Q3:** $F(1, 16) = 0.25, p = 0.625$ ($BF_{10} = 0.084$)
Subjective trust	**Q1:** $F(1, 23) = 0.01, p = 0.94$ ($BF_{10} = 0.071$)<br>**Q2:** $F(1, 23) = 0.46, p = 0.503$ ($BF_{10} = 0.099$)<br>**Q3:** $F(1, 23) = 0.41, p = 0.529$ ($BF_{10} = 0.086$)
**Theme 4: Social alignment with interaction partners**	
Social alignment	**Q1:** $F(1, 20) = 5.07, p = 0.036$ ($BF_{10} = 0.452$)<br>**Q2:** $F(1, 20) = 2.13, p = 0.160$ ($BF_{10} = 0.177$)<br>**Q3:** $F(1, 20) = 1.86, p = 0.188$ ($BF_{10} = 0.113$)

**Responses**	**Meta-regression results** (Q1: study design rigour; Q2: data & reporting rigour; Q3: broad research integrity)
**Theme 5: Personal agency and task performance**	
Perceived self-agency	*insufficient studies (k = 9, m = 14)*
Self-disclosure	*insufficient studies (k = 9, m = 21)*
Strategic economic behaviour	**Q1:** $F(1, 11) = 0.85, p = 0.376$ ($BF_{10} = 0.076$)<br>**Q2:** $F(1, 11) = 0.45, p = 0.518$ ($BF_{10} = 0.076$)<br>**Q3:** $F(1, 11) = 0.00, p = 0.970$ ($BF_{10} = 0.055$)
Objective task performance	**Q1:** $F(1, 21) = 0.04, p = 0.843$ ($BF_{10} = 0.067$)<br>**Q2:** $F(1, 36) = 0.55, p = 0.464$ ($BF_{10} = 0.079$)<br>**Q3:** $F(1, 21) = 0.57, p = 0.457$ ($BF_{10} = 0.074$)
**Theme 6: Interaction experiences**	
Perceived partner relational qualities	**Q1:** $F(1, 15) = 2.71, p = 0.121$ ($BF_{10} = 0.405$)<br>**Q2:** $F(1, 15) = 0.42, p = 0.525$ ($BF_{10} = 0.146$)<br>**Q3:** $F(1, 15) = 4.18, p = 0.059$ ($BF_{10} = 0.708$)
Affective valence	**Q1:** $F(1, 8) = 0.05, p = 0.825$ ($BF_{10} = 0.162$)<br>**Q2:** $F(1, 8) = 0.32, p = 0.588$ ($BF_{10} = 0.196$)<br>**Q3:** $F(1, 8) = 0.67, p = 0.438$ ($BF_{10} = 0.245$)
Affective arousal	*insufficient studies (k = 6, m = 11)*
Interaction satisfaction	**Q1:** $F(1, 17) = 0.02, p = 0.881$ ($BF_{10} = 0.108$)<br>**Q2:** $F(1, 17) = 6.19, p = 0.024$ ($BF_{10} = 1.590$)<br>**Q3:** $F(1, 17) = 1.08, p = 0.313$ ($BF_{10} = 0.168$)
Future interaction intention	*insufficient studies (k = 6, m = 7)*
Perceived interaction naturalness	*insufficient studies (k = 5, m = 5)*
Perceived interaction enjoyment	*insufficient studies (k = 7, m = 10)*
Subjective workload	*insufficient studies (k = 5, m = 14)*
Subjective task engagement	**Q1:** $F(1, 8) = 0.54, p = 0.485$ ($BF_{10} = 0.172$)<br>**Q2:** $F(1, 8) = 1.35, p = 0.279$ ($BF_{10} = 0.260$)<br>**Q3:** $F(1, 8) = 0.00, p = 0.951$ ($BF_{10} = 0.111$)

Supplementary Table 13. Meta-analyses incorporating approximated effect sizes

We performed three sensitivity checks: (1) incorporating approximated effect sizes, (2) removing outliers and influential cases, and (3) using alternative Bayesian priors. Results remained largely consistent with the main analyses, except for deviations observed in subjective trust, perceived social presence, and certain interaction experiences. Detailed results and interpretations of these deviations are presented in Supplementary Tables 13–15. Overall, sensitivity checks confirmed that our main meta-analytic results were robust to various analytic decisions.

In the following, we first report the sensitivity analysis incorporating approximated effect sizes. We approximated missing effect sizes by estimating from thresholds, deriving statistically adjusted estimates, or imputing non-significant effects (max^{+}, max^{-}, and *zero*-coded). Overall, sensitivity analyses incorporating these approximations yielded results largely consistent with the complete-data analyses, except for perceived social presence, subjective trust, perceived partner relational qualities, and affective valence (highlighted in orange below). These four responses also had relatively high numbers of approximated effect sizes (9 - 12) compared with the other responses that showed robust findings.

In complete-data analyses, Bayesian evidence for subjective trust and affective valence was ambiguous, rendering findings for these two responses inconclusive. Sensitivity analyses incorporating approximated effect sizes further yielded conflicting results that failed to resolve this uncertainty.

For perceived social presence, the frequentist results became non-significant when all nine non-significant effects were imputed as the maximum positive value (max^{+}), but remained significant when they were imputed as *zero* or the maximum negative value (max^{-}). Bayesian evidence shifted from substantial support for H_1 to ambiguity under *zero*/max^{+} imputation, yet rose to very strong support for H_1 under max^{-} imputation. These highlighted a critical dependency of the statistical support on how missing non-significant effects were handled: Although agent partners were consistently perceived as less socially present than human partners, the strength of the conclusion depended on defensible assumptions about those missing effects.

For perceived partner relational qualities, ten approximated effect sizes were included: eight imputed non-significant effects and two statistically adjusted estimates. Frequentist results remained non-significant when the eight non-significant effects were imputed as *zero* or max^{+}, but became significant under max^{-} imputation. Bayesian evidence shifted from substantial support for H_0 to ambiguity, with this change primarily driven by one statistically adjusted estimate, i.e., -1.516 [-1.81, -1.22][142]. Notably, this statistically adjusted estimate represents an outlier, diverging significantly from both the unadjusted effects in the complete dataset and other approximated effects. Therefore, we argue that the absence of a partner effect on perceived partner relational qualities remains robust; but further empirical studies with better-resolved data are needed to resolve this outlier-driven discrepancy and explore underlying moderators.

**Responses**	**Approximated effect sizes (ES)**	**Frequentist meta-analytic results**	**Frequentist heterogeneity metrics**	**Bayesian evidence (BF_{10})**
**Theme 1: Prosociality and morality**				

Responses	Approximated effect sizes (ES)		Frequentist meta-analytic results	Frequentist heterogeneity metrics	Bayesian evidence (BF_{10})
Prosocial behaviour	1 ES[132]		-0.590 [-0.82, -0.36]<br>$t(9) = -5.75$, $p < 0.001$	$Q(10) = 22.55$, $p = 0.013$<br>$I^2 = 59.47\%$	278.585
Moral engagement	2 ES[78]		-0.393 [-0.49, -0.29]<br>$t(8) = -9.20$, $p < 0.01$	$Q(15) = 5.22$, $p = 0.990$<br>$I^2 = 0.00\%$	8394.864
**Theme 2: Social perceptions of interaction partners**					
Perceived social presence	9 ES[54,128,143,144]	*max*$^+$	-0.083 [-0.36, 0.20]<br>$t(12) = -0.64$, $p = 0.533$	$Q(28) = 115.21$, $p < 0.001$<br>$I^2 = 82.59\%$	0.170
		max$^-$	-0.327 [-0.49, -0.16]<br>$t(12) = -4.39$, $p < 0.001$	$Q(28) = 53.46$, $p = 0.003$<br>$I^2 = 55.21\%$	64.494
		zero	-0.208 [-0.39, -0.03]<br>$t(12) = -2.52$, $p = 0.027$	$Q(28) = 58.40$, $p < 0.001$<br>$I^2 = 61.89\%$	1.979
Perceived likeability	3 ES[54,143,144]	*max*$^+$	-0.283 [-0.47, -0.09]<br>$t(30) = -3.05$, $p = 0.005$	$Q(43) = 478.22$, $p < 0.001$<br>$I^2 = 89.13\%$	6.266
		max$^-$	-0.359 [-0.53, -0.18]<br>$t(30) = -4.18$, $p < 0.001$	$Q(43) = 444.32$, $p < 0.001$<br>$I^2 = 87.24\%$	136.486
		zero	-0.320 [-0.50, -0.14]<br>$t(30) = -3.69$, $p < 0.001$	$Q(43) = 450.28$, $p < 0.001$<br>$I^2 = 87.54\%$	28.516
Perceived competence	6 ES[53,78,144,145]	*max*$^+$	-0.335 [-0.50, -0.17]<br>$t(26) = -4.10$, $p < 0.001$	$Q(36) = 265.94$, $p < 0.001$<br>$I^2 = 84.93\%$	79.814
		max$^-$	-0.403 [-0.55, -0.26]<br>$t(26) = -5.82$, $p < 0.001$	$Q(36) = 180.51$, p < 0.001<br>$I^2 = 78.91\%$	7049.129
		zero	-0.381 [-0.53, -0.24]<br>$t(26) = -5.41$, $p < 0.001$	$Q(36) = 197.63$, $p < 0.001$<br>$I^2 = 79.70\%$	2450.161
Agency attribution	/		/	/	/
Responsibility attribution	/		/	/	/
**Theme 3: Trust in interaction partners**					
Behavioural trust	5 ES[20,146–148]	*max*$^+$	0.001 [-0.12, 0.12]<br>$t(22) = 0.02$, $p = 0.986$	$Q(28) = 60.74$, $p < 0.001$<br>$I^2 = 60.59\%$	0.065
		max$^-$	-0.059 [-0.17, 0.05]<br>$t(22) = -1.08$, $p = 0.293$	$Q(28) = 55.95$, $p = 0.001$<br>$I^2 = 56.69\%$	0.124
		zero	-0.021 [-0.12, 0.08]<br>$t(22) = -0.42$, $p = 0.678$	$Q(28) = 48.87$, $p = 0.009$<br>$I^2 = 49.01\%$	0.063
Subjective trust	12 ES[20,54,108,133,142,146,147]	*max*$^+$	-0.069 [-0.19, 0.05]<br>$t(29) = -1.19$, $p = 0.242$	$Q(46) = 175.20$, $p < 0.001$<br>$I^2 = 78.54\%$	0.137
		max$^-$	-0.154 [-0.26, -0.05]<br>$t(29) = -2.99$, $p = 0.006$	$Q(46) = 140.03$, $p < 0.001$<br>$I^2 = 73.33\%$	3.737
		zero	-0.104 [-0.21, 0.00]<br>$t(29) = -2.04$, $p = 0.051$	$Q(46) = 135.72$, $p < 0.001$<br>$I^2 = 72.75\%$	0.458
**Theme 4: Social alignment with interaction partners**					
Social alignment	14 ES[53,94,110,145,149–151]	*max*$^+$	-0.003 [-0.09, 0.09]<br>$t(27) = -0.07$, $p = 0.948$	$Q(49) = 95.57$, $p < 0.001$<br>$I^2 = 53.65\%$	0.049
		max$^-$	-0.075 [-0.16, 0.01]<br>$t(27) = -1.80$, $p = 0.084$	$Q(49) = 80.73$, $p = 0.003$<br>$I^2 = 46.53\%$	0.245

Responses	Approximated effect sizes (ES)		Frequentist meta-analytic results	Frequentist heterogeneity metrics	Bayesian evidence (BF_{10})
		zero	-0.025 [-0.09, 0.04]<br>$t(27) = -0.75, p = 0.460$	$Q(49) = 63.73, p = 0.077$<br>$I^2 = 26.74\%$	0.050
**Theme 5: Personal agency and task performance**					
Perceived self-agency	1 ES[152]	max^+	0.037 [-0.11, 0.19]<br>$t(9) = 0.56, p = 0.588$	$Q(14) = 22.18, p = 0.075$<br>$I^2 = 41.94\%$	0.095
		max^-	-0.016 [-0.17, 0.13]<br>$t(9) = -0.23, p = 0.816$	$Q(14) = 22.21, p = 0.074$<br>$I^2 = 41.59\%$	0.086
		zero	0.009 [-0.13, 0.15]<br>$t(9) = 0.14, p = 0.891$	$Q(14) = 18.51, p = 0.185$<br>$I^2 = 33.95\%$	0.079
Self-disclosure	1 ES[128]	max^+	0.055 [-0.13, 0.24]<br>$t(9) = 0.68, p = 0.511$	$Q(21) = 31.79, p = 0.061$<br>$I^2 = 47.78\%$	0.104
		max^-	-0.008 [-0.19, 0.18]<br>$t(9) = -0.09, p = 0.929$	$Q(21) = 31.67, p = 0.063$<br>$I^2 = 48.40\%$	0.089
		zero	0.022 [-0.15, 0.19]<br>$t(9) = 0.30, p = 0.773$	$Q(21) = 28.03, p = 0.139$<br>$I^2 = 41.66\%$	0.081
Strategic economic behaviour	/		/	/	/
Objective task performance	11 ES[127,132,146,152]	max^+	0.055 [-0.06, 0.17]<br>$t(25) = 0.99, p = 0.332$	$Q(48) = 102.57, p < 0.001$<br>$I^2 = 53.51\%$	0.118
		max^-	-0.069 [-0.18, 0.04]<br>$t(25) = -1.32, p = 0.198$	$Q(48) = 99.02, p < 0.001$<br>$I^2 = 49.26\%$	0.145
		zero	-0.008 [-0.10, 0.08]<br>$t(25) = -0.19, p = 0.852$	$Q(48) = 70.63, p = 0.018$<br>$I^2 = 33.66\%$	0.047
**Theme 6: Interaction experiences**					
Perceive partner relational qualities	10 ES[49,54,128,142,153]	max^+	-0.183 [-0.43, 0.07]<br>$t(20) = -1.52, p = 0.143$	$Q(34) = 281.12, p < 0.001$<br>$I^2 = 92.71\%$	0.402
		max^-	-0.254 [-0.49, -0.01]<br>$t(20) = -2.21, p = 0.039$	$Q(34) = 250.39, p < 0.001$<br>$I^2 = 92.03\%$	1.250
		zero	-0.193 [-0.43, 0.04]<br>$t(20) = -1.70, p = 0.106$	$Q(34) = 244.53, p < 0.001$<br>$I^2 = 91.95\%$	0.521
Affective valence	12 ES[21,128,146,154,155]	max^+	-0.066 [-0.33, 0.20]<br>$t(13) = -0.54, p = 0.599$	$Q(25) = 134.32, p < 0.001$<br>$I^2 = 85.67\%$	0.146
		max^-	-0.262 [-0.49, -0.03]<br>$t(13) = -2.47, p = 0.028$	$Q(25) = 96.66, p < 0.001$<br>$I^2 = 81.17\%$	1.959
		zero	-0.132 [-0.36, 0.09]<br>$t(13) = -1.27, p = 0.227$	$Q(25) = 87.19, p < 0.001$<br>$I^2 = 80.47\%$	0.250
Affective arousal	/		/	/	/
Interaction satisfaction	2 ES[54,142]	max^+	0.049 [-0.18, 0.28]<br>$t(20) = 0.44, p = 0.665$	$Q(28) = 374.36, p < 0.001$<br>$I^2 = 96.05\%$	0.123
		max^-	0.004 [-0.23, 0.23]<br>$t(20) = 0.03, p = 0.974$	$Q(28) = 371.23, p < 0.001$<br>$I^2 = 96.05\%$	0.122
		zero	0.026 [-0.20, 0.25]<br>$t(20) = 0.24, p = 0.816$	$Q(28) = 368.91, p < 0.001$<br>$I^2 = 95.87\%$	0.113

**Responses**	**Approximated effect sizes (ES)**		**Frequentist meta-analytic results**	**Frequentist heterogeneity metrics**	**Bayesian evidence (BF_{10})**
Future interaction intention	2 ES[153]		0.075 [-0.12, 0.27]<br>$t(6) = 0.95$, $p = 0.380$	$Q(8) = 17.46$, $p = 0.026$<br>$I^2 = 52.37\%$	0.148
Perceived interaction naturalness	/		/	/	/
Perceived interaction enjoyment	3 ES[54,142]	max^{+}	-0.214 [-0.74, 0.31]<br>$t(8) = -0.94$, $p = 0.375$	$Q(12) = 152.88$, $p < 0.001$<br>$I^2 = 92.64\%$	0.349
		max^{-}	-0.358 [-0.83, 0.11]<br>$t(8) = -1.75$, $p = 0.119$	$Q(12) = 128.04$, $p < 0.001$<br>$I^2 = 90.92\%$	0.962
		zero	-0.287 [-0.76, 0.19]<br>$t(8) = -1.40$, $p = 0.199$	$Q(12) = 133.31$, $p < 0.001$<br>$I^2 = 91.01\%$	0.620
Subjective workload	/		/	/	/
Subjective task engagement	/		/	/	/

Supplementary Table 14. Outliers and influential cases

The results remained largely the same after excluding outliers and influential cases, except for subjective trust, affective valence, and subjective task engagement (highlighted in orange below). First, for subjective trust, the frequentist results shifted to significance ($p = 0.073$ to 0.039), despite a slight decrease in effect size from -0.109 [-0.23, 0.01] to -0.100 [-0.19, -0.01]. Bayesian evidence for the null hypothesis remained ambiguous ($BF_{10} = 0.358$ to 0.414). Second, for affective valence, the frequentist results remained non-significant ($p = 0.171$ to 0.473), while the effect size decreased from -0.226 [-0.57, 0.12] to -0.069 [-0.28, 0.14]. Bayesian evidence shifted from ambiguous to substantial support for the null hypothesis ($BF_{10} = 0.410$ to 0.114). Third, for subjective task engagement, the frequentist results remained non-significant ($p = 0.585$ to 0.151), while the effect size increased from -0.063 [-0.31, 0.19] to -0.142 [-0.35, 0.06]. Bayesian evidence shifted from substantial support for the null hypothesis to ambiguity ($BF_{10} = 0.168$ to 0.334). In addition, heterogeneity in effect sizes decreased for all responses after excluding outliers and influential cases.

We retained these outlying and influential effects in all analyses presented in the main manuscript, as they were from studies meeting predefined eligibility criteria, and their exclusion did not alter overall patterns of results. Furthermore, it has been argued that the identification of outliers or influential cases does not automatically justify their removal[156].

**Responses**	**Outliers (O) and influential cases (I)**	**Frequentist meta-analytic results**	**Frequentist heterogeneity metrics**	**Bayesian evidence (BF_{10})**
**Theme 1: Prosociality and morality**				
Prosocial behaviour	**O:** /<br>**I:** -0.982 [-1.37, -0.59][70]	-0.590 [-0.75, -0.43]<br>$t(7) = -8.98, p < 0.001$	$Q(8) = 7.33, p = 0.502$<br>$I^2 = 0\%$	2037.749
Moral engagement	**O/I:** /	/	/	/
**Theme 2: Social perceptions of interaction partners**				
Perceived social presence	**O:** 0.541 [0.07, 1.02][44]<br>**I:** -0.674 [-0.93, -0.42][65]	-0.241 [-0.38, -0.10]<br>$t(6) = -4.26, p = 0.005$	$Q(17) = 27.55, p = 0.051$<br>$I^2 = 34.57\%$	13.648
Perceived likeability	**O:** 0.718 [0.35, 1.09][102]<br>**O:** 1.030 [0.65, 1.42][102]<br>**I:** /	-0.367 [-0.55, -0.18]<br>$t(27) = -4.13, p < 0.001$	$Q(38) = 362.87, p < 0.001$<br>$I^2 = 87.03\%$	125.665
Perceived competence	**O:** 0.318 [-0.03, 0.66][63]<br>**I:** /	-0.465 [-0.61, -0.32]<br>$t(22) = -6.56, p < 0.001$	$Q(29) = 126.49$, p < 0.001<br>$I^2 = 75.74\%$	26075.208
Agency attribution	**O:** -2.055 [-2.40, -1.71][82]<br>**O:** 0.581 [0.28, 0.88][71]<br>**I:** /	-0.685 [-1.00, -0.37]<br>$t(8) = -5.02, p = 0.001$	$Q(15) = 101.41$, p < 0.001<br>$I^2 = 86.38\%$	142.439
Responsibility attribution	**O/I:** /	/	/	/
**Theme 3: Trust in interaction partners**				
Behavioural trust	**O:** 0.507 [0.16, 0.86][138]<br>**O:** 0.786 [0.22, 1.35][140]<br>**I:** /	-0.089 [-0.20, 0.02]<br>$t(15) = -1.73, p = 0.104$	$Q(21) = 27.77, p = 0.147$<br>$I^2 = 35.95\%$	0.237
Subjective trust	**O:** -0.950 [-1.26, -0.64][130]<br>**O:** 0.488 [0.19, 0.79][88]	-0.100 [-0.19, -0.01]<br>$t(23) = -2.19, p = 0.039$	$Q(32) = 72.19, p < 0.001$<br>$I^2 = 62.83\%$	0.414

Responses	Outliers (O) and influential cases (I)	Frequentist meta-analytic results	Frequentist heterogeneity metrics	Bayesian evidence (BF_{10})
	**I:** /			
**Theme 4: Social alignment with interaction partners**				
Social alignment	**O:** -0.409 [-0.71, -0.11][84]<br>**O:** 0.618 [0.17, 1.07][157]<br>**I:** /	-0.002 [-0.08, 0.08]<br>$t(19) = -0.06, p = 0.954$	$Q(33) = 38.52, p = 0.234$<br>$I^2 = 15.62\%$	0.047
**Theme 5: Personal agency and task performance**				
Perceived self-agency	**O/I:** -0.492 [-0.79, -0.19][84]	0.060 [-0.07, 0.19]<br>$t(7) = 1.13, p = 0.296$	$Q(12) = 6.93, p = 0.862$<br>$I^2 = 0\%$	0.124
Self-disclosure	**O:** 0.437 [0.12, 0.75][115]<br>**I:** /	-0.026 [-0.20, 0.15]<br>$t(7) = -0.36, p = 0.729$	$Q(19) = 19.88, p = 0.402$<br>$I^2 = 31.24\%$	0.088
Strategic economic behaviour	**O:** -0.672 [-1.19, -0.16][40]<br>**I:** /	-0.071 [-0.16, 0.01]<br>$t(12) = -1.81, p = 0.096$	$Q(22) = 27.58, p = 0.190$<br>$I^2 = 21.10\%$	0.165
Objective task performance	**O:** 1.399 [0.72, 2.08][66]<br>**O:** -0.596 [-0.90, -0.29][100]<br>**I:** /	0.003 [-0.09, 0.09]<br>$t(21) = 0.06, p = 0.950$	$Q(35) = 40.16, p = 0.252$<br>$I^2 = 16.56\%$	0.052
**Theme 6: Interaction experiences**				
Perceived partner relational qualities	**O:** -1.007 [-1.27, -0.75][81]<br>**I:** /	-0.102 [-0.33, 0.13]<br>$t(15) = -0.94, p = 0.363$	$Q(23) = 117.93, p < 0.001$<br>$I^2 = 89.70\%$	0.181
Affective valence	**O/I:** -1.320 [-1.64, -1.00][130]	-0.069 [-0.28, 0.14]<br>$t(8) = -0.75, p = 0.473$	$Q(12) = 26.44, p = 0.009$<br>$I^2 = 60.09\%$	0.114
Affective arousal	**O:** 0.437 [0.00, 0.87][73]<br>**I:** -0.442 [-0.81, -0.08][83]	*We did not re-run analyses for this response, as it lacked sufficient studies (k = 4, m = 9) after removing the outlier and influential case.*		
Interaction satisfaction	**O:** -1.068 [-1.52, -0.62][116]<br>**O:** -0.988 [-1.43, -0.54][116]<br>**O:** 1.322 [0.77, 1.88][92]<br>**I:** /	0.097 [-0.05, 0.25]<br>$t(17) = 1.37, p = 0.189$	$Q(23) = 222.85, p < 0.001$<br>$I^2 = 90.21\%$	0.187
Future interaction intention	**O/I:** /	/	/	/
Perceived interaction naturalness	**O/I:** -1.572 [-2.00, -1.14][100]	*We did not re-run analyses for this response, as it lacked sufficient studies (k = 4, m = 4) after removing the outlier and influential case.*		
Perceived interaction enjoyment	**O/I:** /	/	/	/
Subjective workload	**O/I:** /	/	/	/
Subjective task engagement	**O/I:** 0.745 [0.13, 1.36][86]	-0.142 [-0.35, 0.06]<br>$t(8) = -1.59, p = 0.151$	$Q(9) = 12.88, p = 0.168$<br>$I^2 = 35.74\%$	0.334

Supplementary Table 15. Bayesian meta-analyses under different priors

Main priors:

1. Cauchy(0, 1/√2) + inverse-Gamma(1, 0.15) + inverse-Gamma(1, 0.1)

Alternative priors used in sensitivity analyses:

2. Cauchy(0, 1/√2) + half-Cauchy(0, 0.3) + half-Cauchy(0, 0.2)
3. Student-*t*(3, 0, 1) + inverse-Gamma(1, 0.15) + inverse-Gamma(1, 0.1)
4. Student-*t*(3, 0, 1) + half-Cauchy(0, 0.3) + half-Cauchy(0, 0.2)

Bayesian meta-analytic results were largely consistent across different priors, except for the Bayes factors (BF_{10}) for perceived social presence, subjective trust, and perceived interaction enjoyment (highlighted in orange below). For perceived social presence, all four Bayesian models yielded similar 95% Credible Intervals (CrIs) that excluded zero, in line with the frequentist meta-analysis showing that participants perceived agent partners as significantly less socially present than human partners. However, BF_{10} diverged: Bayesian evidence for this effect was substantial under Priors 1 and 3, yet ambiguous under Priors 2 and 4. Priors 2 and 4 used heavy-tailed half-Cauchy priors for the heterogeneity parameters, which diluted evidence in favour of H_1 by allocating prior mass to large heterogeneity values that did not align with the data, thereby yielding a more ambiguous BF_{10}. Thus, the result that agent partners were perceived as less socially present than human partners was robust, although the strength of Bayesian evidence for this effect was sensitive to prior choice.

For subjective trust, all Bayesian 95% CrIs included zero, in line with the frequentist analysis revealing no significant difference in participants' subjective trust towards agent vs. human partners. However, BF_{10} diverged: Bayesian evidence for the null hypothesis was ambiguous under Priors 1 and 2, yet became substantial under Priors 3 and 4. Priors 3 and 4 used a wider Student-*t*(3, 0, 1) prior for effect sizes, which yielded more Bayesian evidence in favour of H_0 by diffusing prior mass over a wider range of effect sizes, including values inconsistent with the data. Thus, there was no statistical difference in participants' subjective trust towards agent vs. human partners; however, Bayesian evidence for the absence of the effect was sensitive to prior choice and was ambiguous under pre-specified main priors, suggesting whether partner type truly has no effect on subjective trust remains unclear.

Similarly, for perceived interaction enjoyment, all Bayesian 95% CrIs included zero, in line with the frequentist analysis revealing no significant difference in participants' perceived enjoyment of interaction with agent vs. human partners. However, Bayesian evidence was ambiguous under Priors 1 and 2, yet became substantial under Priors 3 and 4 due to the use of a wider Student-*t*(3, 0, 1) prior for effect sizes. Thus, whether partner type truly has no effect on perceived interaction enjoyment remains unclear.

In addition, it should be mentioned that Bayesian estimation based on Markov Chain Monte Carlo (MCMC) sampling relies on stochastic algorithms and floating-point arithmetic, so results vary slightly across different computing environments, even with the same seed. Differences in processor architecture, software versions, or parallelisation protocols can all introduce small numerical discrepancies. These do not affect inferential conclusions, but small deviations in posterior estimates and CrIs should be anticipated when replicating these analyses.

**Responses**	**Priors**	**Hedges' *g* [95% credible interval]**	**(between-study τ^2 / within-study τ^2)**	**Bayesian evidence (BF_{10})**

Responses	Priors	Hedges' *g* [95% credible interval]	(between-study τ^2 / within-study τ^2)	Bayesian evidence (BF_{10})
**Theme 1: Prosociality and morality**				
Prosocial behaviour	1	-0.650 [-0.84, -0.47]	(0.015 / 0.010)	6003.065
	2	-0.647 [-0.84, -0.46]	(0.014 / 0.011)	3381.409
	3	-0.655 [-0.84, -0.47]	(0.016 / 0.010)	6999.537
	4	-0.654 [-0.85, -0.47]	(0.014 / 0.011)	3871.350
Moral engagement	1	-0.371 [-0.49, -0.25]	(0.006 / 0.004)	1725.850
	2	-0.371 [-0.48, -0.26]	(0.003 / 0.002)	1852.961
	3	-0.373 [-0.49, -0.26]	(0.006 / 0.004)	1647.334
	4	-0.374 [-0.48, -0.26]	(0.003 / 0.002)	1766.339
**Theme 2: Social perceptions of interaction partners**				
Perceived social presence	1	-0.269 [-0.48, -0.07]	(0.043 / 0.023)	3.495
	2	-0.269 [-0.50, -0.06]	(0.046 / 0.026)	2.815
	3	-0.274 [-0.49, -0.07]	(0.042 / 0.023)	3.177
	4	-0.274 [-0.51, -0.07]	(0.047 / 0.025)	2.567
Perceived likeability	1	-0.338 [-0.53, -0.15]	(0.132 / 0.077)	41.417
	2	-0.336 [-0.53, -0.15]	(0.136 / 0.079)	34.399
	3	-0.342 [-0.53, -0.15]	(0.133 / 0.076)	35.895
	4	-0.344 [-0.54, -0.16]	(0.139 / 0.077)	37.041
Perceived competence	1	-0.449 [-0.59, -0.30]	(0.081 / 0.009)	10296.030
	2	-0.449 [-0.60, -0.30]	(0.084 / 0.008)	8760.404
	3	-0.453 [-0.60, -0.31]	(0.081 / 0.009)	10210.998
	4	-0.452 [-0.60, -0.30]	(0.084 / 0.008)	8752.483
Agency attribution	1	-0.642 [-1.04, -0.22]	(0.333 / 0.069)	16.554
	2	-0.638 [-1.05, -0.22]	(0.340 / 0.075)	15.100
	3	-0.665 [-1.07, -0.26]	(0.343 / 0.064)	18.721
	4	-0.668 [-1.07, -0.26]	(0.341 / 0.074)	16.887
Responsibility attribution	1	-0.490 [-0.69, -0.30]	(0.045 / 0.047)	512.117
	2	-0.490 [-0.70, -0.29]	(0.049 / 0.052)	360.302
	3	-0.495 [-0.69, -0.30]	(0.045 / 0.047)	530.028
	4	-0.497 [-0.71, -0.29]	(0.048 / 0.053)	382.454
**Theme 3: Trust in interaction partners**				
Behavioural trust	1	-0.023 [-0.15, 0.11]	(0.032 / 0.009)	0.077
	2	-0.023 [-0.15, 0.12]	(0.033 / 0.009)	0.078
	3	-0.023 [-0.15, 0.12]	(0.032 / 0.009)	0.065
	4	-0.023 [-0.15, 0.12]	(0.034 / 0.009)	0.064
Subjective trust	1	-0.107 [-0.23, 0.01]	(0.028 / 0.036)	0.358
	2	-0.107 [-0.23, 0.01]	(0.027 / 0.038)	0.335
	3	-0.109 [-0.23, 0.01]	(0.028 / 0.035)	0.286
	4	-0.109 [-0.23, 0.01]	(0.025 / 0.039)	0.291
**Theme 4: Social alignment with interaction partners**				
Social alignment	1	0.000 [-0.09, 0.09]	(0.013 / 0.007)	0.052
	2	0.001 [-0.09, 0.09]	(0.012 / 0.007)	0.053
	3	0.001 [-0.09, 0.10]	(0.013 / 0.007)	0.043
	4	0.001 [-0.09, 0.10]	(0.011 / 0.007)	0.043
**Theme 5: Personal agency and task performance**				

Responses	Priors	Hedges' *g* [95% credible interval]	(between-study τ^2 / within-study τ^2)	Bayesian evidence (BF_{10})
Perceived self-agency	1	0.009 [-0.15, 0.16]	(0.016 / 0.011)	0.084
	2	0.009 [-0.15, 0.17]	(0.015 / 0.012)	0.086
	3	0.009 [-0.15, 0.17]	(0.016 / 0.011)	0.070
	4	0.009 [-0.15, 0.18]	(0.015 / 0.012)	0.070
Self-disclosure	1	0.022 [-0.14, 0.19]	(0.030 / 0.007)	0.089
	2	0.022 [-0.15, 0.20]	(0.035 / 0.006)	0.093
	3	0.021 [-0.15, 0.19]	(0.030 / 0.007)	0.073
	4	0.020 [-0.16, 0.20]	(0.034 / 0.007)	0.077
Strategic economic behaviour	1	-0.083 [-0.19, 0.02]	(0.012 / 0.005)	0.246
	2	-0.083 [-0.18, 0.01]	(0.011 / 0.003)	0.249
	3	-0.083 [-0.19, 0.02]	(0.012 / 0.005)	0.204
	4	-0.083 [-0.19, 0.02]	(0.011 / 0.003)	0.205
Objective task performance	1	0.004 [-0.11, 0.12]	(0.020 / 0.015)	0.062
	2	0.005 [-0.11, 0.12]	(0.018 / 0.018)	0.060
	3	0.004 [-0.10, 0.12]	(0.020 / 0.015)	0.050
	4	0.006 [-0.10, 0.12]	(0.018 / 0.018)	0.053
**Theme 6: Interaction experiences**				
Perceive partner relational qualities	1	-0.148 [-0.38, 0.08]	(0.184 / 0.011)	0.320
	2	-0.144 [-0.37, 0.09]	(0.187 / 0.012)	0.313
	3	-0.150 [-0.38, 0.08]	(0.183 / 0.011)	0.262
	4	-0.151 [-0.38, 0.09]	(0.187 / 0.012)	0.252
Affective valence	1	-0.196 [-0.50, 0.10]	(0.164 / 0.020)	0.410
	2	-0.196 [-0.51, 0.10]	(0.173 / 0.020)	0.401
	3	-0.206 [-0.52, 0.10]	(0.169 / 0.018)	0.363
	4	-0.207 [-0.53, 0.10]	(0.178 / 0.019)	0.366
Affective arousal	1	-0.078 [-0.30, 0.15]	(0.029 / 0.016)	0.168
	2	-0.077 [-0.32, 0.18]	(0.031 / 0.019)	0.173
	3	-0.080 [-0.31, 0.16]	(0.029 / 0.017)	0.143
	4	-0.080 [-0.33, 0.18]	(0.033 / 0.020)	0.152
Interaction satisfaction	1	0.073 [-0.12, 0.27]	(0.085 / 0.079)	0.154
	2	0.073 [-0.12, 0.27]	(0.091 / 0.081)	0.146
	3	0.075 [-0.12, 0.27]	(0.085 / 0.079)	0.129
	4	0.076 [-0.12, 0.28]	(0.089 / 0.081)	0.123
Future interaction intention	1	0.104 [-0.10, 0.31]	(0.014 / 0.023)	0.212
	2	0.104 [-0.10, 0.31]	(0.011 / 0.027)	0.218
	3	0.108 [-0.09, 0.31]	(0.013 / 0.022)	0.177
	4	0.105 [-0.10, 0.32]	(0.012 / 0.027)	0.185
Perceived interaction naturalness	1	-0.291 [-0.91, 0.30]	(0.501 / NA)	0.585
	2	-0.277 [-0.86, 0.35]	(0.506 / NA)	0.577
	3	-0.321 [-0.98, 0.34]	(0.510 / NA)	0.561
	4	-0.322 [-0.94, 0.33]	(0.506 / NA)	0.548
Perceived interaction enjoyment	1	-0.179 [-0.57, 0.23]	(0.192 / 0.055)	0.355
	2	-0.173 [-0.57, 0.24]	(0.204 / 0.062)	0.382
	3	-0.189 [-0.59, 0.24]	(0.187 / 0.058)	0.328
	4	-0.180 [-0.61, 0.27]	(0.205 / 0.064)	0.333

Responses	Priors	Hedges' *g* [95% credible interval]	(between-study τ^2 / within-study τ^2)	Bayesian evidence (BF_{10})
Subjective workload	1	-0.143 [-0.42, 0.15]	(0.049 / 0.028)	0.320
	2	-0.145 [-0.45, 0.17]	(0.060 / 0.031)	0.329
	3	-0.154 [-0.45, 0.15]	(0.050 / 0.029)	0.275
	4	-0.154 [-0.47, 0.17]	(0.059 / 0.033)	0.280
Subjective task engagement	1	-0.072 [-0.29, 0.16]	(0.040 / 0.017)	0.168
	2	-0.071 [-0.29, 0.18]	(0.044 / 0.019)	0.173
	3	-0.077 [-0.29, 0.17]	(0.039 / 0.017)	0.140
	4	-0.071 [-0.30, 0.18]	(0.043 / 0.020)	0.142

Supplementary Table 16. Bayesian meta-analyses for different response types in human-agent vs. human-human interactions

It should be mentioned that Bayesian estimation based on MCMC sampling relies on stochastic algorithms and floating-point arithmetic, so results vary slightly across different computing environments, even with the same seed. Differences in processor architecture, software versions, or parallelisation protocols can all introduce small numerical discrepancies. These do not affect inferential conclusions, but small deviations in posterior estimates and CrIs should be anticipated when replicating these analyses.

Response	*g*_Bayes	95% CrI	τ^2 (bs/ws)	$\hat{R} \leq 1.01$	ESS ≥ 1000	BF_{10}	*k*	*m*	*g*_Freq	95% CI
**Theme 1: Prosociality and morality**										
Prosocial behaviour	-0.650	[-0.84, -0.47]	0.015/0.010	Y	Y	6003.065	9	10	-0.648	[-0.82, -0.48]
Moral engagement	-0.371	[-0.49, -0.25]	0.006/0.004	Y	Y	1725.850	8	14	-0.376	[-0.48, -0.27]
**Theme 2: Social perceptions of interaction partners**										
Perceived social presence	-0.269	[-0.48, -0.07]	0.043/0.023	Y	Y	3.495	9	20	-0.284	[-0.52, -0.05]
Perceived likeability	-0.338	[-0.53, -0.15]	0.132/0.077	Y	Y	41.417	28	41	-0.352	[-0.54, -0.16]
Perceived competence	-0.449	[-0.59, -0.30]	0.081/0.009	Y	Y	10296.030	23	31	-0.457	[-0.61, -0.30]
Agency attribution	-0.642	[-1.04, -0.22]	0.333/0.069	Y	Y	16.554	11	18	-0.705	[-1.16, -0.25]
Responsibility attribution	-0.490	[-0.69, -0.30]	0.045/0.047	Y	Y	512.117	12	18	-0.511	[-0.72, -0.30]
**Theme 3: Trust in interaction partners**										
Behavioural trust	-0.023	[-0.15, 0.11]	0.032/0.009	Y	Y	0.077	18	24	-0.020	[-0.16, 0.12]
Subjective trust	-0.107	[-0.23, 0.01]	0.028/0.036	Y	Y	0.358	25	35	-0.109	[-0.23, 0.01]
**Theme 4: Social alignment with interaction partners**										
Social alignment	0.000	[-0.09, 0.09]	0.013/0.007	Y	Y	0.052	22	36	-0.004	[-0.10, 0.09]
**Theme 5: Personal agency and task performance**										
Perceived self-agency	0.009	[-0.15, 0.16]	0.016/0.011	Y	Y	0.084	9	14	0.010	[-0.14, 0.16]
Self-disclosure	0.022	[-0.14, 0.19]	0.030/0.007	Y	Y	0.089	9	21	0.025	[-0.16, 0.21]
Strategic economic behaviour	-0.083	[-0.19, 0.02]	0.012/0.005	Y	Y	0.246	13	24	-0.086	[-0.18, 0.01]
Objective task performance	0.004	[-0.11, 0.12]	0.020/0.015	Y	Y	0.062	23	38	0.008	[-0.10, 0.12]
**Theme 6: Interaction experiences**										
Perceived partner relational qualities	-0.148	[-0.38, 0.08]	0.184/0.011	Y	Y	0.320	17	25	-0.157	[-0.40, 0.09]
Affective valence	-0.196	[-0.50, 0.10]	0.164/0.020	Y	Y	0.410	10	14	-0.226	[-0.57, 0.12]
Affective arousal	-0.078	[-0.30, 0.15]	0.029/0.016	Y	Y	0.168	6	11	-0.087	[-0.34, 0.17]
Interaction satisfaction	0.073	[-0.12, 0.27]	0.085/0.079	Y	Y	0.154	19	27	0.085	[-0.12, 0.29]
Future interaction intention	0.104	[-0.10, 0.31]	0.014/0.023	Y	Y	0.212	6	7	0.105	[-0.10, 0.31]
Perceived interaction naturalness	-0.291	[-0.91, 0.30]	0.501/NA	Y	Y	0.585	5	5	-0.368	[-1.24, 0.50]
Perceived interaction enjoyment	-0.179	[-0.57, 0.23]	0.192/0.055	Y	Y	0.355	7	10	-0.182	[-0.69, 0.32]

Response	*g*_Bayes	95% CrI	τ^2 (bs/ws)	$\hat{R} \leq$ 1.01	ESS ≥ 1000	BF_{10}	*k*	*m*	*g*_Freq	95% CI
Subjective workload	-0.143	[-0.42, 0.15]	0.049/0.028	Y	Y	0.320	5	14	-0.157	[-0.54, 0.23]
Subjective task engagement	-0.072	[-0.29, 0.16]	0.040/0.017	Y	Y	0.168	10	11	-0.063	[-0.31, 0.19]

Note. Results in this table were based on the following priors: Cauchy(0, 1/√2) prior for the effect size, inverse-Gamma(1, 0.15) prior for between-study variance, and inverse-Gamma(1, 0.1) prior for within-study variance. Sensitivity analyses using alternative priors are reported in Supplementary Table 15. Each model was run using 4 chains with 10,000 iterations (5,000 warmup) per chain; the target acceptance probability during Stan's adaption period was set at 0.99 and the maximum tree depth was set at 10. Specifically, *g*_Bayes is the pooled Hedges' *g* from Bayesian meta-analysis, and 95% CrI is the corresponding 95% credible interval. Hedges' *g* (*g*_Freq) and 95% CI from frequentist analysis are also presented to facilitate comparison between two approaches. τ^2 (bs/ws) denotes the between-study and within-study variances. $\hat{R} \leq 1.01$ (Y) and ESS ≥ 1000 (Y) for all parameters indicate good model convergence. BF_{10} indicates the strength of Bayesian evidence in favour of H_1 over H_0. *k* is the number of studies; *m* is the number of effect sizes.

Supplementary Table 17. Meta-analytic results for response themes in human-agent vs. human-human interactions

The main manuscript primarily presented meta-analytic results for different types of human responses. These findings are consistent with the results of the theme-level meta-analyses. Notably, although the theme-level analysis yielded a statistically significant pooled effect for interaction experiences, the magnitude of this effect was small (Hedges' g = -0.127, 95% CI [-0.25, 0.00]) with high heterogeneity (I^2 = 91.15%). Bayesian meta-analysis indicated ambiguous evidence in support of the null effect (BF_{10} = 0.587). After excluding outliers, the theme-level effect was no longer statistically significant. In addition, none of the response types within the theme of "interaction experiences" showed reliable differences between human-agent and human-human interactions.

**Response themes**	**Hedges' *g***	**95% CI**	***t***	***t_p***	***Q***	***Q_p***	**I^2 (%)**	**BF_{10} under main priors**	**BF_{10} under alternative priors**	***k***	***m***	**Hedges' *g* [95% CI] after excluding outliers and influential cases**
Prosociality and morality	-0.465	[-0.54, -0.39]	-12.43	< 0.001	25.53	0.324	1.22	9133076.187	*a.* 6367802.402<br>*b.* 9431603.931<br>*c.* 6493181.325	17	24	-0.446 [-0.53, -0.37]
Social perceptions of interaction partners	-0.436	[-0.55, -0.32]	-7.68	< 0.001	1012.83	< 0.001	87.07	34753254.678	*a.* 27738559.199<br>*b.* 35642478.483<br>*c.* 29285554.739	50	128	-0.450 [-0.54, -0.35]
Trust in interaction partners	-0.077	[-0.17, 0.02]	-1.63	0.113	164.72	< 0.001	72.37	0.222	*a.* 0.206<br>*b.* 0.183<br>*c.* 0.175	32	59	-0.082 [-0.16, -0.01]
Social alignment	-0.004	[-0.10, 0.09]	-0.08	0.937	52.90	0.027	33.11	0.052	*a.* 0.053<br>*b.* 0.043<br>*c.* 0.043	22	36	-0.002 [-0.08, 0.08]
Personal agency and task performance	-0.025	[-0.09, 0.04]	-0.83	0.408	153.03	< 0.001	39.43	0.043	*a.* 0.047<br>*b.* 0.035<br>*c.* 0.039	51	97	-0.020 [-0.07, 0.03]
Interaction experiences	-0.127	[-0.25, 0.00]	-2.07	0.043	811.91	< 0.001	91.15	0.587	*a.* 0.561<br>*b.* 0.496<br>*c.* 0.464	54	124	-0.093 [-0.20, 0.01]

Note. This table presents results primarily from frequentist meta-analysis. Hedges' *g*, 95% CI, *t*-value and associated *p*-value were estimated via random-effects meta-analysis. *Q* is Cochrane's *Q*-statistic for testing heterogeneity. I^2 is the proportion of total variance attributable to true heterogeneity rather than

sampling error. BF_{10} is presented to provide complementary Bayesian evidence for H_1 over H_0, and is shown under both main and alternative prior distributions. Main priors include: Cauchy(0, 1/√2) + inverse-Gamma(1, 0.15) + inverse-Gamma(1, 0.1). Alternative priors include: *a.* Cauchy(0, 1/√2) + half-Cauchy(0, 0.3) + half-Cauchy(0, 0.2); *b.* Student-*t*(3, 0, 1) + inverse-Gamma(1, 0.15) + inverse-Gamma(1, 0.1); *c.* Student-*t*(3, 0, 1) + half-Cauchy(0, 0.3) + half-Cauchy(0, 0.2). Hedges' *g* and 95% CI were additionally estimated after excluding outliers and influential cases. *k* is the number of studies including in meta-analysis. *m* is the number of effect sizes included.

Supplementary Fig. 1. Funnel plots

Note. Funnel plots were generated for each response type with at least ten studies to visually assess potential publication bias. Each plot displays individual effect sizes (Hedges' g) plotted against their corresponding standard errors. Panels are shown in the following order from left to right and top to bottom: *a.* perceived likeability; *b.* perceived competence; *c.* agency attribution; *d.* responsibility attribution; *e.* behavioural trust; *f.* subjective trust; *g.* social alignment; *h.* strategic economic behaviour; *i.* objective task performance; *j.* perceived partner relational qualities; *k.* affective valence; *l.* interaction satisfaction; *m.* subjective task engagement. The number of included studies and effects varies by response type and is reported in the main meta-analytic results (Table 3).

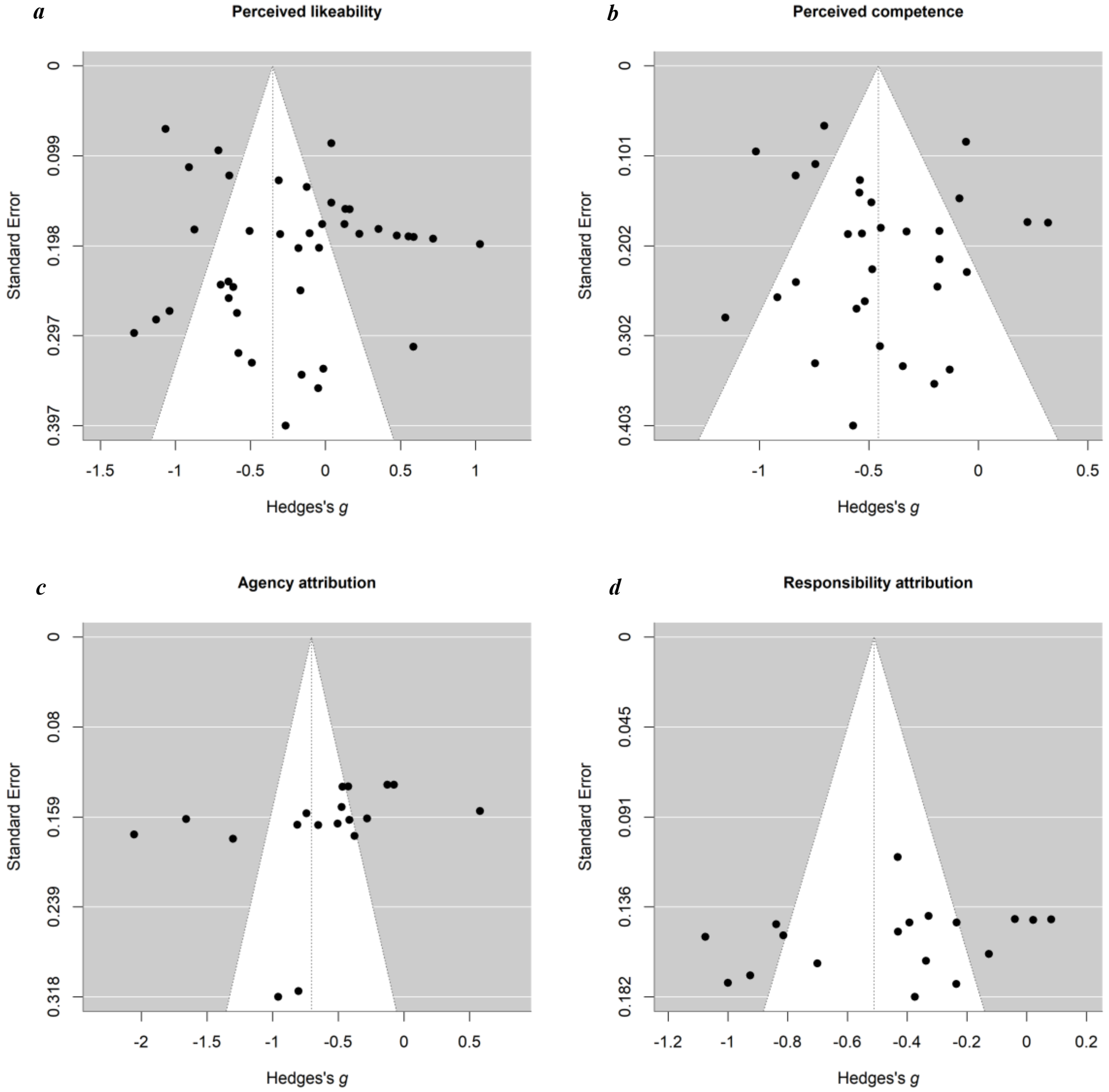

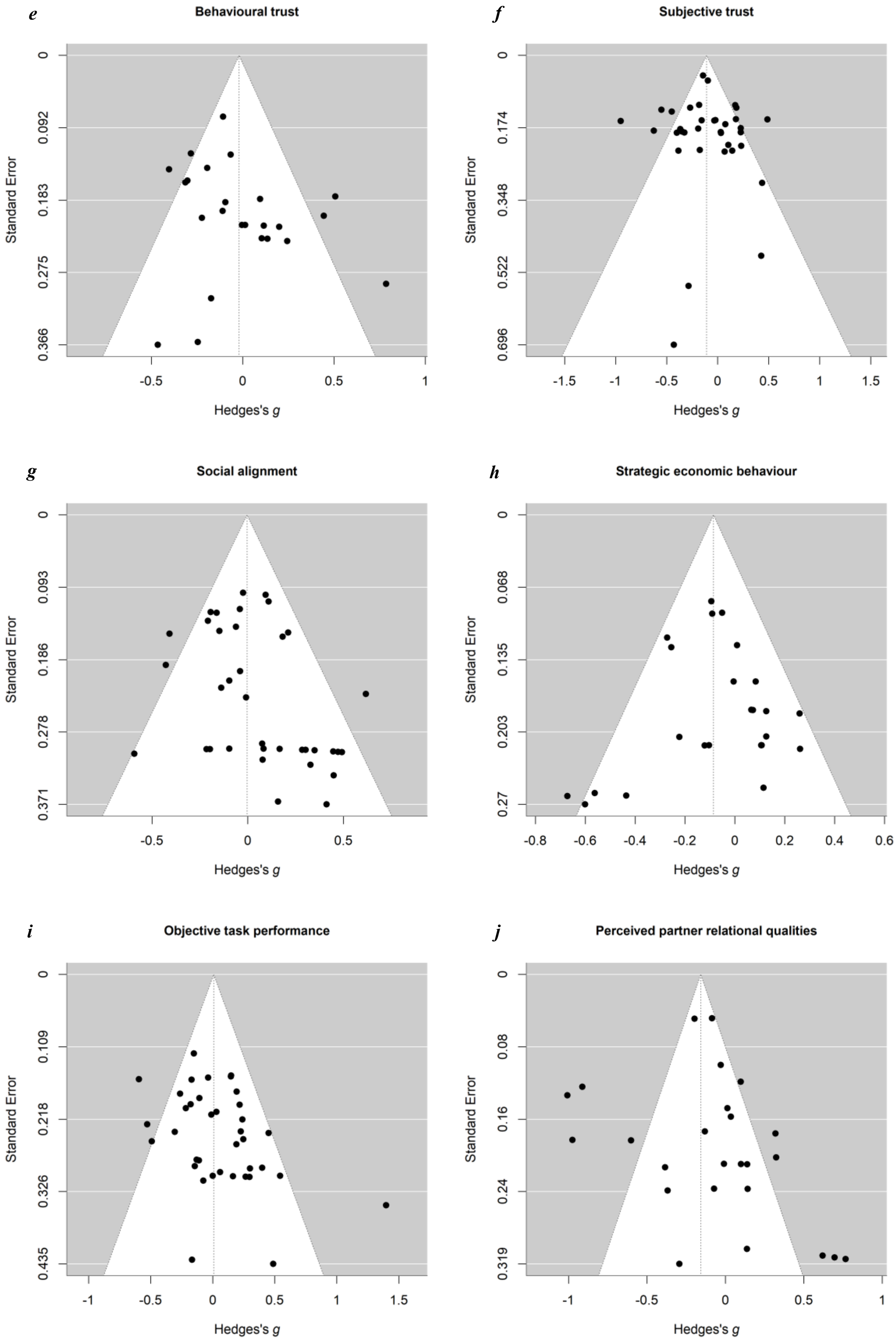
e
Behavioural trust
Standard Error
Hedges's g
f
Subjective trust
Standard Error
Hedges's g
g
Social alignment
Standard Error
Hedges's g
h
Strategic economic behaviour
Standard Error
Hedges's g
i
Objective task performance
Standard Error
Hedges's g
j
Perceived partner relational qualities
Standard Error
Hedges's g

k

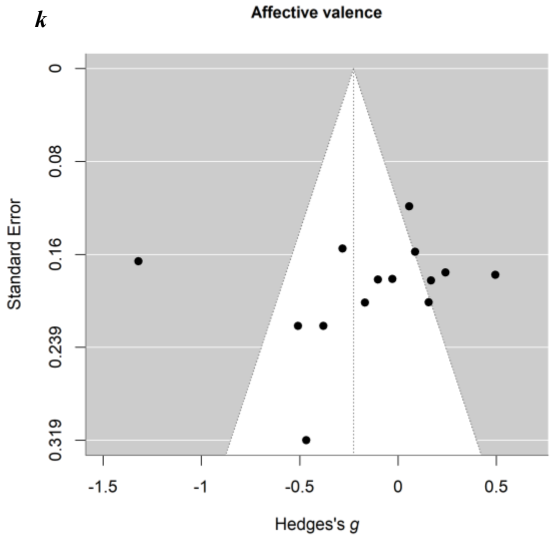
Affective valence
Standard Error
0
0.08
0.16
0.239
0.319
-1.5
-1
-0.5
0
0.5
Hedges's g

l

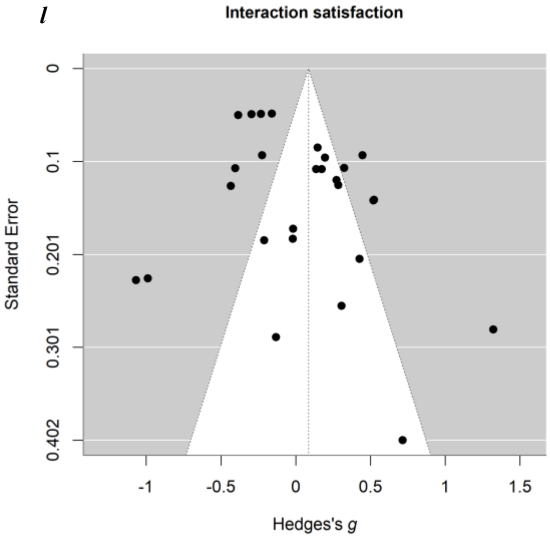
Interaction satisfaction
Standard Error
0
0.1
0.201
0.301
0.402
-1
-0.5
0
0.5
1
1.5
Hedges's g

m

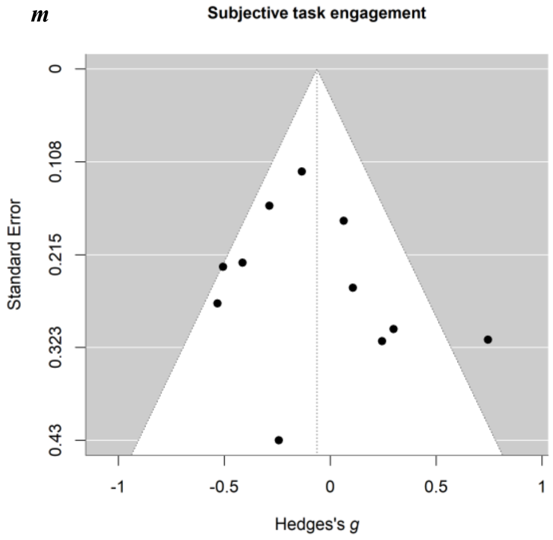
Subjective task engagement
Standard Error
0
0.108
0.215
0.323
0.43
-1
-0.5
0
0.5
1
Hedges's g

Supplementary Note 1. Response classification

We performed a posteriori classification of the diverse human responses investigated across studies. We started with a conceptual-to-empirical approach to classification development[158], applying Krpan's taxonomy[159] (which summarises a comprehensive set of affective, cognitive, and behavioural responses to robots) to guide our classification. To our knowledge, this is currently the only formal classification of human responses to interactive intelligent agents. This taxonomy was developed by collecting participants' recalled or imagined feelings, thoughts, and behaviours during robot interactions, organising all responses into 149 distinct items, and employing exploratory factor analysis to identify three overarching dimensions—positive, negative, and competence—that underlie human responses[159]. Our extracted responses, however, did not align with the taxonomy's 149-response list, nor did the three dimensions emerge. This discrepancy may be due to our review encompassing interactive intelligent agents beyond robots, and to human responses based on recollections of past interactions or interaction expectations differing from the real-time or experimentally induced responses examined in empirical studies.

We therefore shifted to an empirical-to-conceptual approach[158], allowing our classification scheme to arise directly from the dataset. We thoroughly reviewed the descriptions and measures of all human responses extracted from the studies and inductively classified them into distinct response types. We further iteratively grouped conceptually aligned response types into six emergent themes, with a residual category for unclassified responses. The six themes include prosociality and morality, social perceptions of partners, trust in partners, social alignment, personal agency and task performance, and interaction experiences.

Supplementary References

1. Brysbaert, M. How Many Participants Do We Have to Include in Properly Powered Experiments? A Tutorial of Power Analysis with Reference Tables. *J. Cogn.* **2**, 16 (2019).
2. Borenstein, M., Hedges, L. V., Higgins, J. P. T. & Rothstein, H. R. *Introduction to Meta-Analysis*. (Wiley, 2009). doi:10.1002/9780470743386.
3. Lakens, D. Calculating and reporting effect sizes to facilitate cumulative science: a practical primer for t-tests and ANOVAs. *Front. Psychol.* **4**, (2013).
4. Roberts, B. R. T., MacLeod, C. M. & Fernandes, M. A. The Enactment Effect: A Systematic Review and Meta-Analysis of Behavioral, Neuroimaging, and Patient Studies. *Psychol. Bull.* **148**, 397–434 (2022).
5. Drevon, D., Fursa, S. R. & Malcolm, A. L. Intercoder Reliability and Validity of WebPlotDigitizer in Extracting Graphed Data. *Behav. Modif.* **41**, 323–339 (2017).
6. Maassen, E., Van Assen, M. A. L. M., Nuijten, M. B., Olsson-Collentine, A. & Wicherts, J. M. Reproducibility of individual effect sizes in meta-analyses in psychology. *PLOS ONE* **15**, e0233107 (2020).
7. Viechtbauer, W. Assembling Data for a Meta-Analysis of Standardized Mean Differences. *The metafor Package: A Meta-Analysis Package for R* https://www.metafor-project.org/doku.php/tips:assembling_data_smd#fnt__1.
8. *Cochrane Handbook for Systematic Reviews of Interventions*. (Wiley-Blackwell, Hoboken, NJ, 2019).
9. Rowland, C. A. The effect of testing versus restudy on retention: A meta-analytic review of the testing effect. *Psychol. Bull.* **140**, 1432–1463 (2014).
10. Wilson, D. B. Practical Meta Analysis Effect Size Calculator. https://www.campbellcollaboration.org/calculator/equations#binary-dependent-variable-a-2-by-2-frequency-table (2023).
11. Pan, S. C. & Rickard, T. C. Transfer of test-enhanced learning: Meta-analytic review and synthesis. *Psychol. Bull.* **144**, 710–756 (2018).
12. Sambrook, T. D. & Goslin, J. A neural reward prediction error revealed by a meta-analysis of ERPs using great grand averages. *Psychol. Bull.* **141**, 213–235 (2015).
13. Diel, A., Weigelt, S. & Macdorman, K. F. A Meta-analysis of the Uncanny Valley's Independent and Dependent Variables. *ACM Trans. Hum.-Robot Interact.* **11**, 1–33 (2022).
14. Cásedas, L., Pirruccio, V., Vadillo, M. A. & Lupiáñez, J. Does Mindfulness Meditation Training Enhance Executive Control? A Systematic Review and Meta-Analysis of Randomized Controlled Trials in Adults. *Mindfulness* **11**, 411–424 (2020).
15. Wampold, B. E. *et al.* A meta-analysis of outcome studies comparing bona fide psychotherapies: Empiricially, 'all must have prizes.' *Psychol. Bull.* **122**, 203–215 (1997).
16. Xu, K., Chen, M. & You, L. The Hitchhiker's Guide to a Credible and Socially Present Robot: Two Meta-Analyses of the Power of Social Cues in Human–Robot Interaction. *Int. J. Soc. Robot.* **15**, 269–295 (2023).
17. Fox, J. *et al.* Avatars Versus Agents: A Meta-Analysis Quantifying the Effect of Agency on Social Influence. *Human–Computer Interact.* **30**, 401–432 (2015).
18. Morris, S. B. & DeShon, R. P. Combining effect size estimates in meta-analysis with repeated measures and independent-groups designs. *Psychol. Methods* **7**, 105–125 (2002).
19. Abril, T., Oliveira, J. & Gamito, P. Construction and effect of relationships with agents in a virtual reality environment. *Virtual Real.* **27**, 3665–3678 (2023).
20. Alarcon, G. M., Capiola, A., Hamdan, I. A., Lee, M. A. & Jessup, S. A. Differential biases in human-human versus human-robot interactions. *Appl. Ergon.* **106**, 103858 (2023).

21. Appel, J., Von Der Pütten, A., Krämer, N. C. & Gratch, J. Does Humanity Matter? Analyzing the Importance of Social Cues and Perceived Agency of a Computer System for the Emergence of Social Reactions during Human-Computer Interaction. *Adv. Hum.-Comput. Interact.* **2012**, 1–10 (2012).
22. Babel, F., Kraus, J. M. & Baumann, M. Development and Testing of Psychological Conflict Resolution Strategies for Assertive Robots to Resolve Human–Robot Goal Conflict. *Front. Robot. AI* **7**, 591448 (2021).
23. Bah, J.-M., Mostafaoui, G. & Cohen, L. A kinematic study on social intention during a human-robot interaction. in *2022 IEEE International Conference on Development and Learning (ICDL)* 202–207 (IEEE, London, United Kingdom, 2022). doi:10.1109/ICDL53763.2022.9962213.
24. Bailenson, J. N., Blascovich, J., Beall, A. C. & Loomis, J. M. Interpersonal Distance in Immersive Virtual Environments. *Pers. Soc. Psychol. Bull.* **29**, 819–833 (2003).
25. Banks, J. & Van Ouytsel, J. Cybersex with human- and machine-cued partners: Gratifications, shortcomings, and tensions. *Technol. Mind Behav.* **1**, (2020).
26. Bartneck, C., Reichenbach, J. & Carpenter, J. The carrot and the stick: The role of praise and punishment in human–robot interaction. *Interact. Stud. Soc. Behav. Commun. Biol. Artif. Syst.* **9**, 179–203 (2008).
27. Belanche, D., Casaló, L. V., Flavián, C. & Schepers, J. Robots or frontline employees? Exploring customers' attributions of responsibility and stability after service failure or success. *J. Serv. Manag.* **31**, 267–289 (2020).
28. Bergmann, K., Branigan, H. P. & Kopp, S. Exploring the Alignment Space – Lexical and Gestural Alignment with Real and Virtual Humans. *Front. ICT* **2**, (2015).
29. Blankendaal, R., Bosse, T., Gerritsen, C., de Jong, T. & de Man, J. Are Aggressive Agents as Scary as Aggressive Humans? in *Proceedings of the 14th International Conference on Autonomous Agents and Multiagent Systems (AAMAS 2015)* 553–561 (International Foundation for Autonomous Agents and Multiagent Systems (IFAAMAS), Istanbul, Turkey, 2015).
30. Bouquet, C. A. *et al.* Joint action with human and robotic co-actors: Self-other integration is immune to the perceived humanness of the interacting partner. *Q. J. Exp. Psychol.* **77**, 70–89 (2024).
31. Bowman, N. D. & Banks, J. Social and Entertainment Gratifications of Videogame Play Comparing Robot, AI, and Human Partners. in *2019 28th IEEE International Conference on Robot and Human Interactive Communication (RO-MAN)* 1–6 (IEEE, New Delhi, India, 2019). doi:10.1109/RO-MAN46459.2019.8956256.
32. Bunlon, F., Gazeau, J.-P., Colloud, F., Marshall, P. J. & Bouquet, C. A. Joint action with a virtual robotic vs. human agent. *Cogn. Syst. Res.* **52**, 816–827 (2018).
33. Čaić, M., Avelino, J., Mahr, D., Odekerken-Schröder, G. & Bernardino, A. Robotic Versus Human Coaches for Active Aging: An Automated Social Presence Perspective. *Int. J. Soc. Robot.* **12**, 867–882 (2020).
34. Caruana, N. & McArthur, G. The mind minds minds: The effect of intentional stance on the neural encoding of joint attention. *Cogn. Affect. Behav. Neurosci.* **19**, 1479–1491 (2019).
35. Chen, N., Hu, X. & Zhai, Y. Effects of morality and reputation on sharing behaviors in human-robot teams. *Front. Psychol.* **14**, 1280127 (2023).
36. Choi, S., Liu, S. Q. & Mattila, A. S. "How may i help you?" Says a robot: Examining language styles in the service encounter. *Int. J. Hosp. Manag.* **82**, 32–38 (2019).
37. Cominelli, L. *et al.* Promises and trust in human–robot interaction. *Sci. Rep.* **11**, 9687 (2021).

38. Cowan, B. R., Branigan, H. P., Obregón, M., Bugis, E. & Beale, R. Voice anthropomorphism, interlocutor modelling and alignment effects on syntactic choices in human−computer dialogue. *Int. J. Hum.-Comput. Stud.* **83**, 27–42 (2015).
39. De Carolis, B. & Novielli, N. Recognizing signals of social attitude in interacting with Ambient Conversational Systems. *J. Multimodal User Interfaces* **8**, 43–60 (2014).
40. De Kleijn, R., Van Es, L., Kachergis, G. & Hommel, B. Anthropomorphization of artificial agents leads to fair and strategic, but not altruistic behavior. *Int. J. Hum.-Comput. Stud.* **122**, 168–173 (2019).
41. De Melo, C. M. & Terada, K. Cooperation with autonomous machines through culture and emotion. *PLOS ONE* **14**, e0224758 (2019).
42. De Visser, E. J. *et al.* Almost human: Anthropomorphism increases trust resilience in cognitive agents. *J. Exp. Psychol. Appl.* **22**, 331–349 (2016).
43. Desideri, L., Ottaviani, C., Malavasi, M., Di Marzio, R. & Bonifacci, P. Emotional processes in human-robot interaction during brief cognitive testing. *Comput. Hum. Behav.* **90**, 331–342 (2019).
44. Edwards, A., Edwards, C., Westerman, D. & Spence, P. R. Initial expectations, interactions, and beyond with social robots. *Comput. Hum. Behav.* **90**, 308–314 (2019).
45. Edwards, C., Edwards, A., Albrehi, F. & Spence, P. Interpersonal impressions of a social robot versus human in the context of performance evaluations. *Commun. Educ.* **70**, 165–182 (2021).
46. Frank, D.-A. & Otterbring, T. Being seen… by human or machine? Acknowledgment effects on customer responses differ between human and robotic service workers. *Technol. Forecast. Soc. Change* **189**, 122345 (2023).
47. Garvey, A. M., Kim, T. & Duhachek, A. Bad News? Send an AI. Good News? Send a Human. *J. Mark.* **87**, 10–25 (2023).
48. Giroux, M., Kim, J., Lee, J. C. & Park, J. Artificial Intelligence and Declined Guilt: Retailing Morality Comparison Between Human and AI. *J. Bus. Ethics* **178**, 1027–1041 (2022).
49. Go, E. & Sundar, S. S. Humanizing chatbots: The effects of visual, identity and conversational cues on humanness perceptions. *Comput. Hum. Behav.* **97**, 304–316 (2019).
50. Gonzalez-Billandon, J. *et al.* Can a Robot Catch You Lying? A Machine Learning System to Detect Lies During Interactions. *Front. Robot. AI* **6**, 64 (2019).
51. Gratch, J., DeVault, D. & Lucas, G. The Benefits of Virtual Humans for Teaching Negotiation. in *Intelligent Virtual Agents* (eds Traum, D. et al.) vol. 10011 283–294 (Springer International Publishing, Cham, 2016).
52. Mozgai, S., Lucas, G. & Gratch, J. To Tell the Truth: Virtual Agents and Morning Morality. in *Intelligent Virtual Agents* (eds Beskow, J. et al.) vol. 10498 283–286 (Springer International Publishing, Cham, 2017).
53. Guadagno, R. E., Blascovich, J., Bailenson, J. N. & Mccall, C. Virtual Humans and Persuasion: The Effects of Agency and Behavioral Realism. *Media Psychol.* **10**, 1–22 (2007).
54. Guadagno, R. E., Swinth, K. R. & Blascovich, J. Social evaluations of embodied agents and avatars. *Comput. Hum. Behav.* **27**, 2380–2385 (2011).
55. Haring, K. S. *et al.* Robot Authority in Human-Machine Teams: Effects of Human-Like Appearance on Compliance. in *Virtual, Augmented and Mixed Reality. Applications and Case Studies* (eds Chen, J. Y. C. & Fragomeni, G.) vol. 11575 63–78 (Springer International Publishing, Cham, 2019).
56. Harriott, C. E., Zhang, T. & Adams, J. A. Assessing physical workload for human–robot peer-based teams. *Int. J. Hum.-Comput. Stud.* **71**, 821–837 (2013).

57. Hertz, N. & Wiese, E. Influence of Agent Type and Task Ambiguity on Conformity in Social Decision Making. *Proc. Hum. Factors Ergon. Soc. Annu. Meet.* **60**, 313–317 (2016).
58. Hinds, P., Roberts, T. & Jones, H. Whose Job Is It Anyway? A Study of Human-Robot Interaction in a Collaborative Task. *Hum.-Comput. Interact.* **19**, 151–181 (2004).
59. Ho, A., Hancock, J. & Miner, A. S. Psychological, Relational, and Emotional Effects of Self-Disclosure After Conversations With a Chatbot. *J. Commun.* **68**, 712–733 (2018).
60. Hoffman, G. *et al.* Robot Presence and Human Honesty: Experimental Evidence. in *Proceedings of the Tenth Annual ACM/IEEE International Conference on Human-Robot Interaction* 181–188 (ACM, Portland Oregon USA, 2015). doi:10.1145/2696454.2696487.
61. Holthöwer, J. & Van Doorn, J. Robots do not judge: service robots can alleviate embarrassment in service encounters. *J. Acad. Mark. Sci.* **51**, 767–784 (2023).
62. Hoorn, J. F. & Huang, I. S. The media inequality, uncanny mountain, and the singularity is far from near: Iwaa and Sophia robot versus a real human being. *Int. J. Hum.-Comput. Stud.* **181**, 103142 (2024).
63. Horstmann, A. C., Gratch, J. & Krämer, N. C. I just wanna blame somebody, not something! Reactions to a computer agent giving negative feedback based on the instructions of a person. *Int. J. Hum.-Comput. Stud.* **154**, 102683 (2021).
64. Huang, H.-H., Konishi, N., Shibusawa, S. & Kawagoe, K. Can a Virtual Listener Replace a Human Listener in Active Listening Conversation? in *Proceedings of the International Workshop on Emotion Representations and Modelling for Companion Technologies* 33–39 (ACM, Seattle Washington USA, 2015). doi:10.1145/2829966.2829971.
65. Jerčić, P., Wen, W., Hagelbäck, J. & Sundstedt, V. The Effect of Emotions and Social Behavior on Performance in a Collaborative Serious Game Between Humans and Autonomous Robots. *Int. J. Soc. Robot.* **10**, 115–129 (2018).
66. Jois, H. & Wagner, A. R. What Happens When Robots Punish? Evaluating Human Task Performance During Robot-Initiated Punishment. *ACM Trans. Hum.-Robot Interact.* **10**, 1–18 (2021).
67. Kahn, P. H. *et al.* Will People Keep the Secret of a Humanoid Robot?: Psychological Intimacy in HRI. in *Proceedings of the Tenth Annual ACM/IEEE International Conference on Human-Robot Interaction* 173–180 (ACM, Portland Oregon USA, 2015). doi:10.1145/2696454.2696486.
68. Kalashnikova, N., Hutin, M., Vasilescu, I. & Devillers, L. Do We Speak to Robots Looking Like Humans As We Speak to Humans? A Study of Pitch in French Human-Machine and Human-Human Interactions. in *International Cconference on Multimodal Interaction* 141–145 (ACM, Paris France, 2023). doi:10.1145/3610661.3617990.
69. Kalashnikova, N., Hutin, M., Vasilescu, I. & Devillers, L. The Effect of Human-Likeliness in French Robot-Directed Speech: A Study of Speech Rate and Fluency. in *Text, Speech, and Dialogue* (eds Ekštein, K., Pártl, F. & Konopík, M.) vol. 14102 249–257 (Springer Nature Switzerland, Cham, 2023).
70. Karpus, J., Krüger, A., Verba, J. T., Bahrami, B. & Deroy, O. Algorithm exploitation: Humans are keen to exploit benevolent AI. *iScience* **24**, 102679 (2021).
71. Kawai, Y. *et al.* Anthropomorphism-based causal and responsibility attributions to robots. *Sci. Rep.* **13**, 12234 (2023).
72. Khalighinejad, N., Bahrami, B., Caspar, E. A. & Haggard, P. Social Transmission of Experience of Agency: An Experimental Study. *Front. Psychol.* **7**, (2016).
73. Kiilavuori, H., Peltola, M. J., Sariola, V. & Hietanen, J. K. Being watched by a humanoid robot and a human: Effects on affect-related psychophysiological responses. *Biol. Psychol.* **175**, 108451 (2022).

74. Kiilavuori, H., Sariola, V., Peltola, M. J. & Hietanen, J. K. Making eye contact with a robot: Psychophysiological responses to eye contact with a human and with a humanoid robot. *Biol. Psychol.* **158**, 107989 (2021).
75. Kim, M., Kwon, T. & Kim, K. Can Human–Robot Interaction Promote the Same Depth of Social Information Processing as Human–Human Interaction? *Int. J. Soc. Robot.* **10**, 33–42 (2018).
76. Kim, T. W. & Duhachek, A. Artificial Intelligence and Persuasion: A Construal-Level Account. *Psychol. Sci.* **31**, 363–380 (2020).
77. Kim, T. W., Jiang, L., Duhachek, A., Lee, H. & Garvey, A. Do You Mind if I Ask You a Personal Question? How AI Service Agents Alter Consumer Self-Disclosure. *J. Serv. Res.* **25**, 649–666 (2022).
78. Kim, T., Lee, H., Kim, M. Y., Kim, S. & Duhachek, A. AI increases unethical consumer behavior due to reduced anticipatory guilt. *J. Acad. Mark. Sci.* **51**, 785–801 (2023).
79. Krach, S. *et al.* Can Machines Think? Interaction and Perspective Taking with Robots Investigated via fMRI. *PLoS ONE* **3**, e2597 (2008).
80. Kulms, P. & Kopp, S. More Human-Likeness, More Trust?: The Effect of Anthropomorphism on Self-Reported and Behavioral Trust in Continued and Interdependent Human-Agent Cooperation. in *Proceedings of Mensch und Computer 2019* 31–42 (ACM, Hamburg Germany, 2019). doi:10.1145/3340764.3340793.
81. Leiño Calleja, D., Schepers, J. & Nijssen, E. J. Some agents are more similar than others: customer orientation of frontline robots and employees. *J. Serv. Manag.* **34**, 27–49 (2023).
82. Leo, X. & Huh, Y. E. Who gets the blame for service failures? Attribution of responsibility toward robot versus human service providers and service firms. *Comput. Hum. Behav.* **113**, 106520 (2020).
83. Liao, J. & Huang, J. Think like a robot: How interactions with humanoid service robots affect consumers' decision strategies. *J. Retail. Consum. Serv.* **76**, 103575 (2024).
84. Liao, W., Oh, Y. J., Feng, B. & Zhang, J. Understanding the Influence Discrepancy Between Human and Artificial Agent in Advice Interactions: The Role of Stereotypical Perception of Agency. *Commun. Res.* **50**, 633–664 (2023).
85. Liu, Y., Yan, W., Hu, B., Lin, Z. & Song, Y. Chatbots or Humans? Effects of Agent Identity and Information Sensitivity on Users' Privacy Management and Behavioral Intentions: A Comparative Experimental Study between China and the United States. *Int. J. Human–Computer Interact.* **40**, 5632–5647 (2024).
86. Lin, T.-H., Ng, S. & Sebo, S. Benefits of an Interactive Robot Character in Immersive Puzzle Games. in *2022 31st IEEE International Conference on Robot and Human Interactive Communication (RO-MAN)* 37–44 (IEEE, Napoli, Italy, 2022). doi:10.1109/RO-MAN53752.2022.9900828.
87. Liu, N. & Pu, Q. Can Smart Voice Assistant Induce Social Facilitation Effect? A Preliminary Study. in *Cross-Cultural Design. User Experience of Products, Services, and Intelligent Environments* (ed. Rau, P.-L. P.) vol. 12192 616–624 (Springer International Publishing, Cham, 2020).
88. Maehigashi, A., Tsumura, T. & Yamada, S. Experimental Investigation of Trust in Anthropomorphic Agents as Task Partners. in *Proceedings of the 10th International Conference on Human-Agent Interaction* 302–305 (ACM, Christchurch New Zealand, 2022). doi:10.1145/3527188.3563921.
89. Maggioni, M. A. & Rossignoli, D. If it looks like a human and speaks like a human ... Communication and cooperation in strategic Human–Robot interactions. *J. Behav. Exp. Econ.* **104**, 102011 (2023).

90. Mell, J., Lucas, G. & Gratch, J. Prestige Questions, Online Agents, and Gender-Driven Differences in Disclosure. in *Intelligent Virtual Agents* (eds Beskow, J. et al.) vol. 10498 273–282 (Springer International Publishing, Cham, 2017).
91. Meng, J. & Dai, Y. (Nancy). Emotional Support from AI Chatbots: Should a Supportive Partner Self-Disclose or Not? *J. Comput.-Mediat. Commun.* **26**, 207–222 (2021).
92. Merkle, M. Customer responses to service robots comparing human-robot interaction with human-human interaction. in *Proceedings of the 52nd Hawaii International Conference on System Sciences* 1396–1405 (IEEE Computer Society, 2019).
93. Merritt, T., Ong, C., Chuah, T. L. & McGee, K. Did You Notice? Artificial Team-Mates Take Risks for Players. in *Intelligent Virtual Agents* (eds Vilhjálmsson, H. H., Kopp, S., Marsella, S. & Thórisson, K. R.) vol. 6895 338–349 (Springer Berlin Heidelberg, Berlin, Heidelberg, 2011).
94. Mirbabaie, M. *et al.* Understanding Collaboration with Virtual Assistants – The Role of Social Identity and the Extended Self. *Bus. Inf. Syst. Eng.* **63**, 21–37 (2021).
95. Naito, M., Rea, D. J. & Kanda, T. Hey Robot, Tell It to Me Straight: How Different Service Strategies Affect Human and Robot Service Outcomes. *Int. J. Soc. Robot.* **15**, 969–982 (2023).
96. Ng, Y.-L. When communicative AIs are cooperative actors: a prisoner's dilemma experiment on human–communicative artificial intelligence cooperation. *Behav. Inf. Technol.* **42**, 2141–2151 (2023).
97. Nishio, S., Ogawa, K., Kanakogi, Y., Itakura, S. & Ishiguro, H. Do Robot Appearance and Speech Affect People's Attitude? Evaluation Through the Ultimatum Game. in *Geminoid Studies* (eds Ishiguro, H. & Dalla Libera, F.) 263–277 (Springer Singapore, Singapore, 2018). doi:10.1007/978-981-10-8702-8_16.
98. Numata, T. *et al.* Achieving affective human–virtual agent communication by enabling virtual agents to imitate positive expressions. *Sci. Rep.* **10**, 5977 (2020).
99. Ossadnik, J., Muehlfeld, K. & Goerke, L. Man or machine – or something in between? Social responses to voice assistants at work and their effects on job satisfaction. *Comput. Hum. Behav.* **149**, 107919 (2023).
100. Palanica, A., Thommandram, A. & Fossat, Y. Adult Verbal Comprehension Performance is Better from Human Speakers than Social Robots, but only for Easy Questions. *Int. J. Soc. Robot.* **11**, 359–369 (2019).
101. Pavone, G., Meyer-Waarden, L. & Munzel, A. Rage Against the Machine: Experimental Insights into Customers' Negative Emotional Responses, Attributions of Responsibility, and Coping Strategies in Artificial Intelligence–Based Service Failures. *J. Interact. Mark.* **58**, 52–71 (2023).
102. Pickard, M. D., Schuetzler, R., Valacich, J. S. & Wood, D. A. Innovative Accounting Interviewing: A Comparison of Real and Virtual Accounting Interviewers. *Account. Rev.* **95**, 339–366 (2020).
103. Plaks, J. E., Bustos Rodriguez, L. & Ayad, R. Identifying psychological features of robots that encourage and discourage trust. *Comput. Hum. Behav.* **134**, 107301 (2022).
104. Poinsot, K., Gorisse, G., Christmann, O., Fleury, S. & Richir, S. Effect of Social Actors Perceived Agency on Social Presence in Computer-Mediated Communication. *Adv. Hum.-Comput. Interact.* **2022**, 1–12 (2022).
105. Riether, N., Hegel, F., Wrede, B. & Horstmann, G. Social facilitation with social robots? in *Proceedings of the seventh annual ACM/IEEE international conference on Human-Robot Interaction* 41–48 (ACM, Boston Massachusetts USA, 2012). doi:10.1145/2157689.2157697.
106. Roozen, I., Raedts, M. & Yanycheva, A. Are Retail Customers Ready for Service Robot Assistants? *Int. J. Soc. Robot.* **15**, 15–25 (2023).

107. Russo, P. A., Duradoni, M. & Guazzini, A. How self-perceived reputation affects fairness towards humans and artificial intelligence. *Comput. Hum. Behav.* **124**, 106920 (2021).
108. Ryoo, Y., Jeon, Y. A. & Kim, W. The blame shift: Robot service failures hold service firms more accountable. *J. Bus. Res.* **171**, 114360 (2024).
109. Sandoval, E. B., Brandstetter, J., Obaid, M. & Bartneck, C. Reciprocity in Human-Robot Interaction: A Quantitative Approach Through the Prisoner's Dilemma and the Ultimatum Game. *Int. J. Soc. Robot.* **8**, 303–317 (2016).
110. Sahaï, A. *et al.* Modulations of one's sense of agency during human–machine interactions: A behavioural study using a full humanoid robot. *Q. J. Exp. Psychol.* **76**, 606–620 (2023).
111. Sciutti, A. *et al.* Robots can be perceived as goal-oriented agents. *Interact. Stud. Soc. Behav. Commun. Biol. Artif. Syst.* **14**, 329–350 (2013).
112. Shahverdi, P. *et al.* Emotionally Specific Backchanneling in Social Human-Robot Interaction and Human-Human Interaction. in *2023 IEEE/RSJ International Conference on Intelligent Robots and Systems (IROS)* 4059–4064 (IEEE, Detroit, MI, USA, 2023). doi:10.1109/IROS55552.2023.10341823.
113. Shen, H. & Wang, M. Improving Interaction Experience through Lexical Convergence: The Prosocial Effect of Lexical Alignment in Human-Human and Human-Computer Interactions. *Int. J. Human–Computer Interact.* **38**, 28–41 (2022).
114. Shin, H., Bunosso, I. & Levine, L. R. The influence of chatbot humour on consumer evaluations of services. *Int. J. Consum. Stud.* **47**, 545–562 (2023).
115. Sundar, S. S. & Kim, J. Machine Heuristic: When We Trust Computers More than Humans with Our Personal Information. in *Proceedings of the 2019 CHI Conference on Human Factors in Computing Systems* 1–9 (ACM, Glasgow Scotland Uk, 2019). doi:10.1145/3290605.3300768.
116. Singh, S., Olson, E. D. & Tsai, C.-H. (Ken). Use of service robots in an event setting: Understanding the role of social presence, eeriness, and identity threat. *J. Hosp. Tour. Manag.* **49**, 528–537 (2021).
117. Smyk, N. J., Weiss, S. M. & Marshall, P. J. Sensorimotor Oscillations During a Reciprocal Touch Paradigm With a Human or Robot Partner. *Front. Psychol.* **9**, 2280 (2018).
118. Söderlund, M. Employee encouragement of self-disclosure in the service encounter and its impact on customer satisfaction. *J. Retail. Consum. Serv.* **53**, 102001 (2020).
119. Stock, R. M. & Merkle, M. Customer Responses to Robotic Innovative Behavior Cues During the Service Encounter. in *ICIS 2018 Proceedings* (San Francisco, 2018).
120. Stock-Homburg, R. & Hannig, M. Is There a Privacy Paradox in the Workplace? in (Association for Information Systems, India, 2020).
121. Tatsukawa, K., Takahashi, H., Yoshikawa, Y. & Ishiguro, H. Interpersonal Closeness Correlates With Social Influence on Color Perception Task Using Human and Artificial Agents. *Front. ICT* **5**, 24 (2018).
122. Takahashi, H. *et al.* Different impressions of other agents obtained through social interaction uniquely modulate dorsal and ventral pathway activities in the social human brain. *Cortex* **58**, 289–300 (2014).
123. Terada, K. & Yamada, S. Mind-Reading and Behavior-Reading against Agents with and without Anthropomorphic Features in a Competitive Situation. *Front. Psychol.* **8**, 1071 (2017).
124. Torta, E., Van Dijk, E., Ruijten, P. A. M. & Cuijpers, R. H. The Ultimatum Game as Measurement Tool for Anthropomorphism in Human–Robot Interaction. in *Social Robotics* (eds Herrmann, G. et al.) vol. 8239 209–217 (Springer International Publishing, Cham, 2013).

125. Tsfasman, M. *et al.* Towards a Real-time Measure of the Perception of Anthropomorphism in Human-robot Interaction. in *Proceedings of the 2nd ACM Multimedia Workshop on Multimodal Conversational AI* 13–18 (ACM, Virtual Event China, 2021). doi:10.1145/3475959.3485394.
126. Tu, Y., Xiao, L. & Yang, Z. The Impact of Commercial Chatbots on Customer Relationship Quality: The Mediating Role of Social Perceptions and Psychological Distance*. in *2023 WRC Symposium on Advanced Robotics and Automation (WRC SARA)* 219–224 (IEEE, Beijing, China, 2023). doi:10.1109/WRCSARA60131.2023.10261817.
127. Tuvo, E., Ricciardelli, P. & Ciardo, F. The Effect of Anthropomorphism on Diffusion or Responsibility in HRI. in *Social Robotics* (eds Cavallo, F. et al.) vol. 13818 488–497 (Springer Nature Switzerland, Cham, 2022).
128. Von Der Pütten, A. M., Krämer, N. C., Gratch, J. & Kang, S.-H. "It doesn't matter what you are!" Explaining social effects of agents and avatars. *Comput. Hum. Behav.* **26**, 1641–1650 (2010).
129. Walliser, J., Tulk, S., Hertz, N., Issler, E. & Wiese, E. Effects of Perspective Taking on Implicit Attitudes and Performance in Economic Games. in *Social Robotics* (eds Tapus, A., André, E., Martin, J.-C., Ferland, F. & Ammi, M.) vol. 9388 684–693 (Springer International Publishing, Cham, 2015).
130. Wang, C., Li, Y., Fu, W. & Jin, J. Whether to trust chatbots: Applying the event-related approach to understand consumers' emotional experiences in interactions with chatbots in e-commerce. *J. Retail. Consum. Serv.* **73**, 103325 (2023).
131. Xiao, C., Wu, W., Zhang, J. & Xu, L. Treat robots as humans? Perspective choice in human-human and human-robot spatial language interaction. *Spat. Cogn. Comput.* **23**, 309–329 (2023).
132. Xiao, C., Xu, L., Sui, Y. & Zhou, R. Do People Regard Robots as Human-Like Social Partners? Evidence From Perspective-Taking in Spatial Descriptions. *Front. Psychol.* **11**, 578244 (2021).
133. Xu, J., Bryant, D. G. & Howard, A. Would You Trust a Robot Therapist? Validating the Equivalency of Trust in Human-Robot Healthcare Scenarios. in *2018 27th IEEE International Symposium on Robot and Human Interactive Communication (RO-MAN)* 442–447 (IEEE, Nanjing, 2018). doi:10.1109/ROMAN.2018.8525782.
134. Xu, J., Bryant, D. G., Chen, Y.-P. & Howard, A. Robot therapist versus human therapist: Evaluating the effect of corrective feedback on human motor performance. in *2018 International Symposium on Medical Robotics (ISMR)* 1–6 (IEEE, Atlanta, GA, USA, 2018). doi:10.1109/ISMR.2018.8333308.
135. Yu, C., Schermerhorn, P. & Scheutz, M. Adaptive eye gaze patterns in interactions with human and artificial agents. *ACM Trans. Interact. Intell. Syst.* **1**, 1–25 (2012).
136. Yu, S., Xiong, J. (Jill) & Shen, H. The rise of chatbots: The effect of using chatbot agents on consumers' responses to request rejection. *J. Consum. Psychol.* **34**, 35–48 (2024).
137. Yu, S. & Zhao, L. Emojifying chatbot interactions: An exploration of emoji utilization in human-chatbot communications. *Telemat. Inform.* **86**, 102071 (2024).
138. Zhang, G., Chong, L., Kotovsky, K. & Cagan, J. Trust in an AI versus a Human teammate: The effects of teammate identity and performance on Human-AI cooperation. *Comput. Hum. Behav.* **139**, 107536 (2023).
139. Zhou, Y., Fei, Z., He, Y. & Yang, Z. How Human–Chatbot Interaction Impairs Charitable Giving: The Role of Moral Judgment. *J. Bus. Ethics* **178**, 849–865 (2022).
140. Zonca, J., Folsø, A. & Sciutti, A. Social Influence Under Uncertainty in Interaction with Peers, Robots and Computers. *Int. J. Soc. Robot.* **15**, 249–268 (2023).

141. Yang, Y. & Konrath, S. A systematic review and meta-analysis of the relationship between economic inequality and prosocial behaviour. *Nat. Hum. Behav.* **7**, 1899–1916 (2023).
142. Pozharliev, R. *et al.* Attachment styles moderate customer responses to frontline service robots: Evidence from affective, attitudinal, and behavioral measures. *Psychol. Mark.* **38**, 881–895 (2021).
143. Bailenson, J. N., Blascovich, J., Beall, A. C. & Loomis, J. M. Interpersonal Distance in Immersive Virtual Environments. *Pers. Soc. Psychol. Bull.* **29**, 819–833 (2003).
144. Krämer, N. C., Leiße, L.-M., Hollingshead, A. & Gratch, J. Evaluated by a Machine. Effects of Negative Feedback by a Computer or Human Boss. in *Intelligent Virtual Agents* (eds Beskow, J. et al.) vol. 10498 235–238 (Springer International Publishing, Cham, 2017).
145. Tsai, W. S., Lun, D., Carcioppolo, N. & Chuan, C. Human versus chatbot: Understanding the role of emotion in health marketing communication for vaccines. *Psychol. Mark.* **38**, 2377–2392 (2021).
146. Fahim, M. A. A., Khan, M. M. H., Jensen, T., Albayram, Y. & Coman, E. Do Integral Emotions Affect Trust? The Mediating Effect of Emotions on Trust in the Context of Human-Agent Interaction. in *Designing Interactive Systems Conference 2021* 1492–1503 (ACM, Virtual Event USA, 2021). doi:10.1145/3461778.3461997.
147. Alarcon, G. M., Gibson, A. M., Jessup, S. A. & Capiola, A. Exploring the differential effects of trust violations in human-human and human-robot interactions. *Appl. Ergon.* **93**, 103350 (2021).
148. Wu, J., Paeng, E., Linder, K., Valdesolo, P. & Jr, J. C. B. Trust and Cooperation in Human-Robot Decision Making. in *The 2016 AAAI Fall Symposium Series: Artificial Intelligence for Human-Robot Interaction* 110–116 (AI Access Foundation, 2016).
149. Mills, P. F., Harry, B., Stevens, C. J., Knoblich, G. & Keller, P. E. Intentionality of a co-actor influences sensorimotor synchronisation with a virtual partner. *Q. J. Exp. Psychol.* **72**, 1478–1492 (2019).
150. Corti, K. & Gillespie, A. Co-constructing intersubjectivity with artificial conversational agents: People are more likely to initiate repairs of misunderstandings with agents represented as human. *Comput. Hum. Behav.* **58**, 431–442 (2016).
151. Torre, I., Linard, A., Steen, A., Tumová, J. & Leite, I. Should Robots Chicken?: How Anthropomorphism and Perceived Autonomy Influence Trajectories in a Game-theoretic Problem. in *Proceedings of the 2021 ACM/IEEE International Conference on Human-Robot Interaction* 370–379 (ACM, Boulder CO USA, 2021). doi:10.1145/3434073.3444687.
152. Ciardo, F., Beyer, F., De Tommaso, D. & Wykowska, A. Attribution of intentional agency towards robots reduces one's own sense of agency. *Cognition* **194**, 104109 (2020).
153. Davis, J. L. *et al.* Role-taking and robotic form: an exploratory study of social connection in human-robot interaction. *Int. J. Hum.-Comput. Stud.* **178**, 103094 (2023).
154. Mende, M., Scott, M. L., Van Doorn, J., Grewal, D. & Shanks, I. Service Robots Rising: How Humanoid Robots Influence Service Experiences and Elicit Compensatory Consumer Responses. *J. Mark. Res.* **56**, 535–556 (2019).
155. Jessup, S. A., Afb, W.-P. & Gibson, A. M. Investigating the Effect of Trust Manipulations on Affect over Time in Human-Human versus Human-Robot Interactions. in *Proceedings of the Annual Hawaii International Conference on System Sciences* 553–562 (IEEE Computer Society, 2020).
156. Viechtbauer, W. & Cheung, M. W.-L. Outlier and influence diagnostics for meta-analysis. *Res. Synth. Methods* **1**, 112–125 (2010).

157. Bailenson, J. N., Blascovich, J., Beall, A. C. & Loomis, J. M. Interpersonal Distance in Immersive Virtual Environments. *Pers. Soc. Psychol. Bull.* **29**, 819–833 (2003).
158. Nickerson, R. C., Varshney, U. & Muntermann, J. A method for taxonomy development and its application in information systems. *Eur. J. Inf. Syst.* **22**, 336–359 (2013).
159. Krpan, D., Booth, J. E. & Damien, A. The positive–negative–competence (PNC) model of psychological responses to representations of robots. *Nat. Hum. Behav.* **7**, 1933–1954 (2023).
