# Supplementary material for: A systematic review and meta-analysis of psychological and behavioural responses in human-agent vs. human-human interactions
Source: Commun Psychol. 2026 May 5;4:102. doi: 10.1038/s44271-026-00466-z (PMC13351021; doi:10.1038/s44271-026-00466-z)
Supplement: Supplementary file 1 — Transparent Peer Review file [file 44271_2026_466_MOESM1_ESM.pdf]

nature portfolio

Peer Review File

A systematic review and meta-analysis of psychological and behavioural responses in human-agent vs. human-human interactions

Corresponding Author: Ms Jianan Zhou

This file contains all editorial decision letters in order by version, followed by all author rebuttals in order by version.

Version 0:

Decision Letter:

** Please ensure you delete the link to your author homepage in this e-mail if you wish to forward it to your coauthors **

Dear Ms Zhou,

Thank you for your patience during the peer-review process. Your manuscript titled "Psychological and behavioural responses in human-agent vs. human-human interactions: a systematic review and meta-analysis" has now been seen by 2 reviewers, and I include their comments at the end of this message. They find your work of interest but raised some important points. We are interested in the possibility of publishing your study in Communications Psychology, but would like to consider your responses to these concerns and assess a revised manuscript before we make a final decision on publication.

We therefore invite you to revise and resubmit your manuscript, along with a point-by-point response to the reviewers. Please highlight all changes in the manuscript text file.

Editorially, we consider it crucial that the theoretical framework that a priori motivated your study should be clearly explained in the revised manuscript. Please also clarify the methodological details that the reviewers have raised such as research quality assessment.

As you revise the manuscript in response to these issues, please also implement all requests in the attached Mandatory Revision Requests document. All requirements listed in this document need to be fully met, or the work will be returned to you for further revisions without peer review. This workflow is in place to increase the likelihood that the paper will be accepted for publication. It reduces the number of rounds of revision (and review) and ensures that the reviewers vet a version of the article that is compliant with journal policies. If you have any questions regarding the required revisions, please contact the journal prior to resubmission to avoid a negative outcome.

We are committed to providing a fair and constructive peer-review process. Please don't hesitate to contact us if you wish to discuss the revision in more detail.

Please submit the following items:

- Revised manuscript
- Point-by-point response to the referees' comments
- Mandatory Revision Requests Table (attached).
- Cover letter (as a separate document)

via this link: Link Redacted .

** This url links to your confidential home page and associated information about manuscripts you may have submitted or are reviewing for us. If you wish to forward this email to co-authors, please delete the link to your homepage first **

We hope to receive your revised paper within 8 weeks; please let us know if you aren't able to submit it within this time so that we can discuss how best to proceed. If we don't hear from you, and the revision process takes significantly longer, we may close your file. In this event, we will still be happy to reconsider your paper at a later date, provided it still presents a significant contribution to the literature at that stage.

We look forward to seeing the revised manuscript and thank you for the opportunity to review your work.

Best regards,

Troby Lui

Troby Lui, PhD
Associate Editor
Communications Psychology

REVIEWER EXPERTISE:
Reviewer #1: human-AI interaction, systematic review/meta-analysis
Reviewer #2: human-AI interaction

REVIEWER REPORTS:

Reviewer #1 (Remarks to the Author):

What are the major claims of the paper?
- Reduced prosocial and moral engagement evident in HAI vs HHI
- Creation of a functional vs social model of HAI, whereby, despite differences in social characteristics of interactions, the results indicate that the functional outcomes of HAI interactions did not suffer.
- So, readers come away with the sense that HAI is more procedural than emotional (unlike HHI), but still effective in terms of achieving shared interactional objectives.
- In bringing together the literature of HAI, the paper also addresses theoretical disparity, specifically, for models describing differences psychological processes at play between HHI and HAI.

Are they novel and will they be of interest to others in the community and the wider field? If the conclusions are not original, it would be helpful if you could provide relevant references.
- Novelty: meta-analysis enables comparability of the psychology of HAI and HAI, to inform future agent design
- Paper is well researched and carries a strong critical voice throughout the literature review, highlighting areas of coherence and disparity for future works to investigate
- Builds on previous narrative reviews in this space by taking an empirical, data driven approach
- There is a strong sense of transparency throughout the paper, whereby exclusions are explained in terms of statistical boundaries or limitations (e.g., using the Q-test outcome to identify and omit moderators from the meta-regression analysis)

Is the work convincing, and if not, what further evidence would be required to strengthen the conclusions?
- Clear definitions of key variables, such as 'agent' and 'interaction' help to clarify where this work sits in the context of HAI research
- Inclusion of both frequentist and Bayesian analysis strengthens the validity of the outcomes generated
- The creation of a checklist following methodological revision (reverting to empirical -> conceptual) is well motivated and provides a useful resource for future review/meta-analysis works in this domain. That being said, I was surprised not to see a more thorough research validity category included in the 'Data & reporting rigour' section. Using a previously validated tool is one way to improve validity, but other researcher reported methods such as IRR, manipulation checks, research team discussions, should also be taken into consideration.
- Could the authors describe one study from their sample to convey, with clarity, what a typical HAI vs HHI comparison study comprises? A single case study would enable the reader to clearly grasp the type and format of experiment under assessment.

On a more subjective note, do you feel that the paper will influence thinking in the field? Please feel free to raise any further questions and concerns about the paper.
- Line 52: change deficit to 'differences' as deficit implies some baseline social understanding that has not been achieved
- Lines 114-117: This mission statement outlining the intentions of the paper is hard to follow because the variables of interest are defined with the exact same levels: experimental context (agent vs human) and partner type (agent vs human).

Could the authors please revise this section, simplifying the paragraph structure and clearly defining the key variables for additional clarity.
- Please restate the research questions in the discussion and map these onto the discussion points raised. This will establish greater coherence between the research motivation and outcomes described.
- Line 166: include the sample size median as it looks like the distribution is non-normal
- Line 185: please clarify whom you refer to with 'We' and state their related expertise
- Though is may be a moot point, there remains a distinct lack of commercial and publicly visible agents in the West, so perhaps differences in social and moral HAI assessments are artefacts of an ongoing novelty effect? That is, people attribute lower levels of these characteristics as their familiarity with and frequency of interaction with agents remains sparse.
- I feel like there was a missed opportunity to discuss experimental variable trends (i.e., those that were clearly more popular than others) and those that were poorly defined and conceptualised, as indicated by broad CIs. As you have good oversight of the research field, you should also comment on areas for future research based on these more general observations.
- Though they are described in the Method, I would advise that another Table be added to define and describe the partner and setting variables included in the meta-analysis, particularly given the results and consequent theoretical significance of these variables.
- Fig 2 caption: change 'Marks' to 'Ticks'
- Fig 2: Please pool effect sizes per 'Response theme' and display these pooled effect sizes. This will help to visually support the analysis key claims
- Table 3: This is quite unwieldy to read and may be more useful as supplementary material. Could you generate an Orchard plot to include in the paper to represent the data in the table. See here for more info on how to create Orchard plots in R: https://daniel1noble.github.io/orchaRd/
- Line 518: change 'personal agency' to 'perceived self-agency' for consistency
- You say that the other authors checked a random sample of 15% of the coded studies, do you have a citation to support this process?

We would also be grateful if you could comment on the appropriateness and validity of any statistical analysis, as well the ability of a researcher to reproduce the work, given the level of detail provided.
- Having published a meta-analysis and attended workshops in meta-analysis in R I can say that the authors have done their due diligence in the analysis
- They have also supported their frequentist outcomes with equivalent Bayesian analyses, providing additional rigour to the insights generated.
- The process by which 'response types' were validated, initially to be mapped onto an existing framework only to revert to a data-driven process, was informative because, the agile nature of this process can be daunting and uncertain for researchers. I wondered then if the authors might reflect on this process in the discussion, so that future readers feel similarly empowered to change research tactic to suit their objectives.

Reviewer #2 (Remarks to the Author):

Summary and overall evaluation
Thank you for the opportunity to read and review this interesting manuscript. The authors present a systematic review and a meta-analysis of experimental studies comparing interactions with agents (HAI) and human partners (HHI), who performed similarly. The investigated outcomes cover a broad range of variables inductively grouped into six categories: prosociality and morality, social perceptions of interaction partners, trust in interaction partners, social alignment with interaction partners, personal agency and task performance, and interaction experiences.

The authors found significant differences between HAI and HHI in prosocial behavior/moral engagement, perceived competence, likability, and social presence (HAI < HHI). No significant differences emerged regarding social alignment, trust, personal agency, task performance, or interaction experience. Effect size heterogeneity was high in most categories, which is why comprehensive moderator analyses were conducted.

The authors conclude that functional behaviors and interactive experiences are largely similar between HAI and HHI. In contrast, social attributions and moral concerns are reduced in HAI, suggesting that, compared to human partners, agents lack intrinsic value.

The paper will be of high interest to HCI researchers as well as communication scientists (keyword: HMC), psychologists, economists, and others. With the rise of AI-based agents and the narrowing perceptual gap between agents and humans, it becomes increasingly important to examine whether and how perceptions, attitudes, and behaviors differ.

While I find the paper well-written, methodologically rigorous, and practically relevant, I have some concerns about its theoretical relevance, theoretical grounding, and the implications of the findings for theory and practice.

Introduction and theoretical background
The paragraph on empirical evidence (p. 2) is quite short and includes few references. Please provide references for statements such as “inconsistent constructs, divergent methodologies, and isolated investigations” and “Whereas some studies report equivalent responses […], others reveal substantial deficits or qualitatively distinct responses”. A more thorough literature review would strengthen the relevance of the paper.

While the diversity of theoretical approaches is mentioned (p. 2), it remains unclear which theory or theoretical framework

guides this study. What assumptions or expectations do you have? Without this, the study appears highly exploratory. Please clarify your theoretical stance.

Consider integrating the following relevant reviews and meta-analyses (p. 2f.):
Huang, G., & Wang, S. (2023). Is artificial intelligence more persuasive than humans? A meta-analysis. Journal of Communication, 73(6), 552–562. https://doi.org/10.1093/joc/jqad024
This article focuses on the persuasive potential of AI communicators (including chatbots, social robots, virtual influencers, etc., see supplementary material), and uses similar theoretical approaches (Media Equation, MAIN model, anthropomorphism). Depending on how the term "agent" is understood here, the claim on p. 16 ("It is the first meta-analysis to compare a wide range of individuals' psychological and behavioral responses […]") may need revision.

There are several reviews comparing interactions with agents having anthropomorphic (vs. machine-like, technical) features employing similar theoretical reasoning as the review by Xu et al. (2023), which you already cite on p. 2; it is up to you to include them in your review. Including at least some of these would strengthen the theoretical and empirical foundation of your review:
Abercrombie, G., Curry, A. C., Dinkar, T., & Talat, Z. (2023). Mirages: On anthropomorphism in dialogue systems (No. arXiv:2305.09800). arXiv. https://doi.org/10.48550/arXiv.2305.09800
Blut, M., Wang, C., Wünderlich, N. V., & Brock, C. (2021). Understanding anthropomorphism in service provision: A meta-analysis of physical robots, chatbots, and other AI. Journal of the Academy of Marketing Science, 49, 632–658. https://doi.org/10.1007/s11747-020-00762-y
Klein, S. H. (2025). The effects of human-like social cues on social responses towards text-based conversational agents—A meta-analysis. Humanities and Social Sciences Communications, 12(1), 1322. https://doi.org/10.1057/s41599-025-05618-w
Mara, M., Appel, M., & Gnambs, T. (2022). Human-like robots and the uncanny valley: A meta-analysis of user responses based on the Godspeed scales. Zeitschrift Für Psychologie, 230(1), 33–46. https://doi.org/10.31234/osf.io/d4qc3
Oliveira, R., Arriaga, P., Santos, F. P., Mascarenhas, S., & Paiva, A. (2021). Towards prosocial design: A scoping review of the use of robots and virtual agents to trigger prosocial behaviour. Computers in Human Behavior, 114, 106547. https://doi.org/10.1016/j.chb.2020.106547
Roesler, E., Manzey, D., & Onnasch, L. (2021). A meta-analysis on the effectiveness of anthropomorphism in human-robot interaction. Science Robotics, 6(58), 5425. https://doi.org/10.1126/scirobotics.abj5425

While I appreciate that key concepts have been defined (e.g., agent, interaction), the theoretical derivation of the research questions, categories, and moderators would benefit from a more thorough literature review. I understand that, given the numerous variables and space limitations, the authors may have chosen not to include this. However, I believe it is still feasible to do so in a concise manner.

The conceptual framework of the study, including the independent variable, the six categories as well as the moderator categories, could be visualized in a figure for clarity. Including example variables for each category and the moderators would help readers grasp the structure quickly.

Minor comment:
Add the abbreviation MAIN to Sundar's Modality-Agency-Interactivity-Navigability model.

Results
RQ1 reads: "Which psychological and/or behavioral responses have been investigated in HAI compared to HHI?". In the results section, the "compared to HHI" part does not appear to be sufficiently addressed. If the intention was to explore which responses have been investigated in the field (rather than comparing them), consider rephrasing RQ1 for clarity.

The dependent variables are currently defined in Table 1 and at the beginning of each respective results paragraph. How the moderators were defined remains unclear at times (e.g., partner appearance difference, p. 6). Although the authors have provided explanations on pp. 19f. and a detailed table on how they coded each variable in the Supplement, this information should be included in the main document (or at least referred to in the respective section on p. 6). A dedicated section that derives outcome variables and moderators from previous literature (e.g., Why was moderator XY included in the review?), with definitions and operationalizations, within a theoretical or conceptual framework (ending with the conceptual overview figure proposed above) would be ideal.

Consider adding descriptive statistics about the included papers (e.g., ratio of journal articles vs. conference papers) and sample characteristics (e.g., country of origin, mean age, gender distribution) on p. 4. This information is interesting for readers as it provides an overview of the papers and samples that were finally included.

The methods section (in combination with the large Supplement) seems sufficiently detailed to reproduce the analysis. While I am not an expert in Bayesian statistics, I have experience in conducting frequentist meta-analysis. The statistical analysis (i.e., random-effects meta-analysis with the metafor package) seems sound. In addition, I appreciate the application of both frequentist and Bayesian approaches.

Minor comments:
- p. 4: second paragraph in the results section: "[…] psychological and behavioral responses […]"
- Are the summary statistics on p. 4 weighted to take into account different sample sizes (mean age, share of female participants)?
- p. 6: Consider adding the moderator levels of partner appearance difference and interaction task in brackets for clarity.

- p. 7: What is the difference between face-to-face vs. computer-mediated (interaction medium)?
- Use minus signs (–) for negative numbers, not hyphens (-).
- p. 8, Fig. 1: In the field “Records excluded (n = 10,640)”, consider adding “after screening titles and abstracts” for transparency.

Discussion
The discussion begins with a rather long summary of the (many) results (p. 15). Interpretation in light of previous literature is largely missing. Please refer back to the prior reviews cited in the introduction and discuss how your findings align or diverge from them.
The introduction states: “This abundance of existing theoretical frameworks underscores the need for a cross-disciplinary, systematic understanding of human responses in HAI vs. HHI, and identifying factors influencing their similarities or differences” (p. 2). Please revisit this point in the discussion and provide more detailed explanations of how your findings address this need (p. 17). You additionally suggest expanding existing frameworks (Media Equation, Threshold Model of Social Influence) (p. 17). How exactly? There are already developments such as MASA (Modality-Agency-Sociality-Anthropomorphism) that account for different social cues. Please position your contribution relative to these.

Is there prior research affirming or contradicting the statement “this suggests that the cognitive mechanisms underlying core collaborative processes like trust and social alignment may be morphology-agnostic” (p. 17)? Please cite and discuss.

The argument regarding intrinsic value (p. 1, 17), and different contexts (p. 17) may benefit from stronger theoretical grounding. Which theoretical approaches could explain this?

How do you expect response differences between HAI and HHI to evolve with the rise of generative AI? Will they diminish or grow stronger? What does this mean for theory, research, and practice? Please provide a reasoned outlook.

The interpretation of moderator effects appears somewhat generic. Please name and discuss the most important moderators with implications for theory, research, and practice.

Methods
The authors’ claims are convincing with respect to the methodological rigour they applied when conducting the data collection and analysis. Every step of the process is explained transparently (e.g., search strings are provided, and the authors have developed a new tool for assessing study quality).

Minor things:
- According to Viechtbauer (2025), the Knapp-Hartung adjustment is not available for the rma.mv() functon(https://cran.r-project.org/web/packages/metafor/metafor.pdf, p. 177). Please clarify where you used it and where you did not.
- As publication bias is one major concern in meta-analyses, consider including one or two sentences on how you checked for publication bias and whether there were issues in the main results section.

General
The authors have not preregistered their study, although specific protocols exist for systematic reviews and meta-analyses, such as PRISMA-P (https://www.prisma-statement.org/protocols). I do not know why the authors did not preregister their study – I just want to say that fear of deviations should not be the reason. For instance, PRISMA-P entails a standard template for reporting changes and amendments. In the meantime, I appreciate the availability of data and analysis code on osf very much.

The PRISMA checklist was added as as part of the large Supplement, and as a separate Supplement, one is sufficient.

I feel that there is much more information worth reporting in this review, including all the collected data about the papers and samples, as well as the remaining moderator analyses. One elegant way to enhance distribution and reproducibility might be to publish a distinct paper on the data (data descriptor), but this is simply a suggestion.

* TRANSPARENT PEER REVIEW: Communications Psychology uses a transparent peer review system. This means that we publish the editorial decision letters including Reviewers' comments to the authors and the author rebuttal letters online as a supplementary peer review file. However, on author request, confidential information and data can be removed from the published reviewer reports and rebuttal letters prior to publication. If your manuscript has been previously reviewed at another journal, those Reviewers' comments would not form part of the published peer review file.

** Visit Nature Research's author and referees' website at www.nature.com/authors for information about policies, services and author benefits**

Communications Psychology is committed to improving transparency in authorship. As part of our efforts in this direction, we are now requesting that all authors identified as ‘corresponding author’ create and link their Open Researcher and Contributor Identifier (ORCID) with their account on the Manuscript Tracking System prior to acceptance. ORCID helps the scientific community achieve unambiguous attribution of all scholarly contributions. You can create and link your ORCID

from the home page of the Manuscript Tracking System by clicking on ‘Modify my Springer Nature account’ and following the instructions in the link below. Please also inform all co-authors that they can add their ORCIDs to their accounts and that they must do so prior to acceptance.
https://www.springernature.com/gp/researchers/orcid/orcid-for-nature-research

For more information please visit http://www.springernature.com/orcid

If you experience problems in linking your ORCID, please contact the Platform Support Helpdesk.

Version 1:

Decision Letter:

** Please ensure you delete the link to your author homepage in this e-mail if you wish to forward it to your coauthors **

Dear Ms Zhou,

Your manuscript titled "A systematic review and meta-analysis of psychological and behavioural responses in human-agent vs. human-human interactions" has now been seen by our reviewers, whose comments appear below. In light of their advice I am delighted to say that we are happy, in principle, to publish a suitably revised version in Communications Psychology.

We therefore invite you to revise your paper one last time to address the remaining concerns of our reviewers and a list of editorial requests. At the same time we ask that you edit your manuscript to comply with our format requirements and to maximise the accessibility and therefore the impact of your work.

EDITORIAL REQUESTS:

Please review our specific editorial comments and requests regarding your manuscript in the attached "Editorial Requests Table". Please outline your response to each request in the right hand column. Please upload the completed table with your manuscript files as a Related Manuscript file.

If you have any questions or concerns about any of our requests, please do not hesitate to contact me.

SUBMISSION INFORMATION:

In order to accept your paper, we require the files listed here https://www.nature.com/documents/commsj-file-checklist.pdf .

OPEN ACCESS:

Communications Psychology is a fully open access journal. Articles are made freely accessible on publication. For further information about article processing charges, open access funding, and advice and support from Nature Research, please visit https://www.nature.com/commspsychol/open-access

At acceptance, you will be provided with instructions for completing the open access licence agreement on behalf of all authors. This grants us the necessary permissions to publish your paper. Additionally, you will be asked to declare that all required third party permissions have been obtained, and to provide billing information in order to pay the article-processing charge (APC).

* TRANSPARENT PEER REVIEW: Communications Psychology uses a transparent peer review system. On author request, confidential information and data can be removed from the published reviewer reports and rebuttal letters prior to publication. If you are concerned about the release of confidential data, please let us know specifically what information you would like to have removed. Please note that we cannot incorporate redactions for any other reasons.

* CODE AVAILABILITY: All Communications Psychology manuscripts must include a section titled "Code Availability" at the end of the methods section. We require that the custom analysis code supporting your conclusions is made available in a publicly accessible repository at this stage; please choose a repository that generates a digital object identifier (DOI) for the code; the link to the repository and the DOI must be included in the Code Availability statement. Publication as Supplementary Information will not suffice.

* DATA AVAILABILITY:
All Communications Psychology manuscripts must include a section titled "Data Availability" at the end of the Methods section. More information on this policy, is available in the Editorial Requests Table and at http://www.nature.com/authors/policies/data/data-availability-statements-data-citations.pdf.

Please use the following link to submit the above items:
Link Redacted
** This url links to your confidential home page and associated information about manuscripts you may have submitted or be reviewing for us. If you wish to forward this email to co-authors, please delete the link to your homepage first **

We hope to hear from you within two weeks; please let us know if you need more time.

Best regards,

Troby Lui

Troby Lui, PhD
Associate Editor
Communications Psychology

REVIEWERS' COMMENTS:

Reviewer #1 (Remarks to the Author):

Dear authors,

Thank you for addressing the comments provided. I've read through the manuscript again and I believe it is more coherent and thorough than the original submission.

As well as improving the data visualisations depicting the outcomes of your analysis you have fleshed out the theoretical and practical implications of the work, giving it greater authority in this problem space. There are a great many academics and practitioners in the field that will benefit from the results detailed.

Thus, I'm happy to accept the submission in its revised form.

Reviewer #2 (Remarks to the Author):

I appreciate the revisions the authors have made to the manuscript and believe it has improved considerably in terms of theoretical rationale, literature review, and discussion. The clarity of the manuscript has also benefited from the newly added information on the posteriori classification, the inclusion of moderators, and the overview figure. Please double-check the sentence “However, these syntheses largely included studies in which participants evaluated agents without interacting with them” (lines 714/715). My understanding is that the proportion of laboratory and vignette studies was more balanced, so the current phrasing may overstate the dominance of vignette studies.
The discussion section is now much more substantial and well developed. Adding a brief conclusion summarizing the main results and key implications of the study would further strengthen the manuscript and help readers quickly grasp its primary contributions.

** Visit Nature Research's author and referees' website at www.nature.com/authors for information about policies, services and author benefits**

Open Access This Peer Review File is licensed under a Creative Commons Attribution 4.0 International License, which permits use, sharing, adaptation, distribution and reproduction in any medium or format, as long as you give appropriate credit to the original author(s) and the source, provide a link to the Creative Commons license, and indicate if changes were made.
In cases where reviewers are anonymous, credit should be given to 'Anonymous Referee' and the source.
The images or other third party material in this Peer Review File are included in the article's Creative Commons license, unless indicated otherwise in a credit line to the material. If material is not included in the article's Creative Commons license and your intended use is not permitted by statutory regulation or exceeds the permitted use, you will need to obtain permission directly from the copyright holder.
To view a copy of this license, visit https://creativecommons.org/licenses/by/4.0/

Response Letter

A systematic review and meta-analysis of psychological and behavioural responses in human-agent vs. human-human interactions (COMMSPSYCHOL-25-0770A)

Dear Editor and Reviewers,

Thank you for your time and effort in reviewing our manuscript and for the constructive and insightful comments. We have carefully addressed each comment and revised the manuscript accordingly.

In the resubmitted version, changes are highlighted in red. Our point-by-point responses to the two reviewers are provided below.

We would also like to note that we have updated the section order to Introduction, Methods, Results, and Discussion, and written out most abbreviations (e.g., HHI, HAI, HCI, and GenAI) in full to conform to the journal's requirements. Additionally, all figures and tables have been moved to the end of the manuscript to improve readability. These formatting changes are not highlighted in red, as they have been implemented throughout the text.

We are grateful for the thoughtfulness of the comments we have received, and believe they have greatly helped to strengthen the study. We appreciate your consideration of our revised manuscript and are happy to respond to any further questions.

Sincerely,
Authors

Responses to Reviewer #1

Comments	Responses
The creation of a checklist following methodological revision (reverting to empirical -> conceptual) is well motivated and provides a useful resource for future review/meta-analysis works in this domain. That being said, I was surprised not to see a more thorough research validity category included in the 'Data & reporting rigour' section. Using a previously validated tool is one way to improve validity, but other researcher reported methods such as IRR, manipulation checks, research team discussions, should also be taken into consideration.	Thank you for this thoughtful suggestion regarding our research quality assessment checklist. We agree that incorporating the indicators of research validity, such as manipulation checks and data coding rigour, is essential for a comprehensive evaluation.<br><br>**For manipulation checks**: We acknowledge their role in ensuring internal validity. However, their applicability and reporting varied substantially in the included studies. In some studies, manipulation checks were necessary to verify partner type (human or agent). Whereas in others (e.g., those involving physical robots), the manipulation was highly salient; thus, formal checks were

	often omitted in practice. In addition, among studies that reported manipulation checks, the specific items used varied, and reporting on how failed manipulation checks were handled was often inconsistent or absent. Because of this, we found it difficult to operationalise “manipulation checks” as a standardised checklist item with categorical ratings (Yes/Partial/No).<br><br>**For IRR and research team consensus**: We found that, in the included studies, most behavioural outcomes (including linguistic ones) were derived using established methods from prior research and did not involve complex manual coding. Because intensive manual coding was restricted to a very small subset of studies, we opted not to include a dedicated item for data coding procedures in the quality checklist.<br><br>Nevertheless, we recognise that as the field matures, these indicators will become increasingly critical. We have added a discussion of these validity indicators (Lines 827-836) and explicitly mention them as critical considerations for future reviews.
Could the authors describe one study from their sample to convey, with clarity, what a typical HAI vs HHI comparison study comprises? A single case study would enable the reader to clearly grasp the type and format of experiment under assessment.	As suggested, we have now added an example study in the Methods (Lines 245-254). This example study illustrates the type of studies synthesised in our review, in which partner type (agent vs. human) is manipulated while functional equivalence between partners is maintained through identical scripts and matched delivery.
Line 52: change deficit to 'differences' as deficit implies some baseline social understanding that has not been achieved	We have replaced “deficits” with “differences” (Line 80).
Lines 114-117: This mission statement outlining the intentions of the paper is hard to follow because the variables of interest are defined with the exact same levels: experimental context (agent vs human) and partner type (agent vs human). Could the authors please revise this section, simplifying the paragraph structure and	We have simplified the wording of the mission statement to improve clarity and readability (Lines 143-148).

clearly defining the key variables for additional clarity.	
Please restate the research questions in the discussion and map these onto the discussion points raised. This will establish greater coherence between the research motivation and outcomes described.	We have restructured the Discussion section by restating the four research questions and mapping the discussion points onto each question (p. 14-15).
Line 166: include the sample size median as it looks like the distribution is non-normal	As suggested, we have added the median to the sample size characteristics. Additionally, while re-checking the data used to calculate these descriptive statistics, we identified and corrected a transcription error in one study's sample size; the means and SDs have been updated accordingly (Lines 446-449).
Line 185: please clarify whom you refer to with 'We' and state their related expertise	We have clarified the author roles and expertise involved in the response classification. Specifically, we now state that the classification was conducted by the first author, who has an interdisciplinary background in psychology, human-computer interaction, and AI-based system design, and supervised by two senior co-authors with relevant expertise. This information has been added to the Methods section to improve transparency (Lines 280-284).
Though is may be a moot point, there remains a distinct lack of commercial and publicly visible agents in the West, so perhaps differences in social and moral HAI assessments are artefacts of an ongoing novelty effect? That is, people attribute lower levels of these characteristics as their familiarity with and frequency of interaction with agents remains sparse.	We thank the reviewer for highlighting the potential role of exposure effects. We have now incorporated this point, alongside the rise of generative AI, in the Implications (Lines 875-891). We speculate that increased exposure to these agents may attenuate perceived deficits in social attributes but is unlikely to fully eliminate differences in prosociality and morality.
I feel like there was a missed opportunity to discuss experimental variable trends (i.e., those that were clearly more popular than others) and those that were poorly defined and conceptualised, as indicated by broad CIs. As you have good oversight of the research field, you should also comment on areas for future research based on these more general observations.	We appreciate this constructive comment and agree that discussing experimental variable trends can provide useful insight. We have now added discussion of the uneven distribution of response types, measurement heterogeneity, and the need for further attention to specific response types (Lines 665-685).
Though they are described in the Method, I would advise that another Table be added	As recommended, we have added a new table (Table 1; p.30-31) presenting the

to define and describe the partner and setting variables included in the meta-analysis, particularly given the results and consequent theoretical significance of these variables.	moderators and their coding scheme. This table defines each moderator variable and specifies their coding levels to improve clarity.
Fig 2 caption: change 'Marks' to 'Ticks'	We have changed "Marks" to "Ticks" in this figure caption (Line 1508).
Fig 2: Please pool effect sizes per 'Response theme' and display these pooled effect sizes. This will help to visually support the analysis key claims	We thank the reviewer for this constructive suggestion. We have conducted additional theme-level meta-analyses and displayed the pooled theme-level effects in this figure as diamond markers (p. 41). Full results of these theme-level meta-analyses are provided in Supplementary Table 17.
Table 3: This is quite unwieldy to read and may be more useful as supplementary material. Could you generate an Orchard plot to include in the paper to represent the data in the table. See here for more info on how to create Orchard plots in R: https://daniel1noble.github.io/orchaRd/	As suggested, we have now visualised the subgroup analysis results using Orchard plots. To retain key statistical information, the corresponding meta-regression results are presented alongside the plots (p. 35-38).
Line 518: change 'personal agency' to 'perceived self-agency' for consistency	We have changed the wording to "perceived self-agency" here (Line 896).
You say that the other authors checked a random sample of 15% of the coded studies, do you have a citation to support this process?	This procedure was informed by prior review work that employed independent reviewer checks on a 15% subset of studies (Line 259). We have now added this paper as a citation to support this approach.
The process by which 'response types' were validated, initially to be mapped onto an existing framework only to revert to a data-driven process, was informative because, the agile nature of this process can be daunting and uncertain for researchers. I wondered then if the authors might reflect on this process in the discussion, so that future readers feel similarly empowered to change research tactic to suit their objectives.	We appreciate this insightful suggestion and have added a reflection on this methodological shift in the Discussion (see Lines 808-816).

Responses to Reviewer #2

Comments	Responses
The paragraph on empirical evidence (p. 2) is quite short and includes few references. Please provide references for statements such as "inconsistent constructs, divergent	As suggested, we have now added relevant references to better support these claims and strengthened the overview of the empirical literature (Lines 63-82).

methodologies, and isolated investigations" and "Whereas some studies report equivalent responses […], others reveal substantial deficits or qualitatively distinct responses". A more thorough literature review would strengthen the relevance of the paper.	
While the diversity of theoretical approaches is mentioned (p. 2), it remains unclear which theory or theoretical framework guides this study. What assumptions or expectations do you have? Without this, the study appears highly exploratory. Please clarify your theoretical stance.	We thank the reviewer for this critical feedback and agree that clarifying the theoretical stance of our work strengthens its conceptual positioning. Existing theoretical perspectives offer overlapping yet partially conflicting predictions regarding the extent to which humans engage with agents in ways resembling interaction with other humans. Our aim was not to defend or dismiss any single theoretical framework. Consequently, we did not adopt a single framework or advance specific a priori hypotheses, as doing so risked constraining the synthesis.<br><br>Instead, we adopted a theory-integrative approach that situates the synthesis within the broader theoretical landscape. Specifically, rather than presuming uniform equivalence or systematic divergence, we treated response comparability as an empirical question to be investigated across distinct psychological and behavioural constructs. This approach allowed our review to quantify the extent of similarity and difference across response types and to identify empirical boundary conditions under which these patterns emerge. We have added a paragraph in the Introduction (Lines 158-167) to clarify this theoretical stance.
Consider integrating the following relevant reviews and meta-analyses (p. 2f.):<br>• *Huang, G., & Wang, S. (2023). Is artificial intelligence more persuasive than humans? A meta-analysis. Journal of Communication, 73(6), 552–562. https://doi.org/10.1093/joc/jqad024*<br>This article focuses on the persuasive potential of AI communicators (including chatbots, social robots, virtual influencers, etc., see supplementary material), and uses	We are grateful to the reviewer for highlighting these relevant reviews and meta-analyses. Huang and Wang (2023) provided an important meta-analytic synthesis of the persuasive potential of AI communicators and found that AI were as persuasive as humans in overall persuasion outcomes. Their operationalisation of persuasion encompassed a broader range

similar theoretical approaches (Media Equation, MAIN model, anthropomorphism). Depending on how the term “agent” is understood here, the claim on p. 16 (“It is the first meta-analysis to compare a wide range of individuals’ psychological and behavioral responses […]”) may need revision.<br><br>There are several reviews comparing interactions with agents having anthropomorphic (vs. machine-like, technical) features employing similar theoretical reasoning as the review by Xu et al. (2023), which you already cite on p. 2; it is up to you to include them in your review. Including at least some of these would strengthen the theoretical and empirical foundation of your review:<br>• *Abercrombie, G., Curry, A. C., Dinkar, T., & Talat, Z. (2023). Mirages: On anthropomorphism in dialogue systems (No. arXiv:2305.09800). arXiv.* https://doi.org/10.48550/arXiv.2305.09800<br>• *Blut, M., Wang, C., Wünderlich, N. V., & Brock, C. (2021). Understanding anthropomorphism in service provision: A meta-analysis of physical robots, chatbots, and other AI. Journal of the Academy of Marketing Science, 49, 632–658.* https://doi.org/10.1007/s11747-020-00762-y<br>• *Klein, S. H. (2025). The effects of human-like social cues on social responses towards text-based conversational agents—A meta-analysis. Humanities and Social Sciences Communications, 12(1), 1322.* https://doi.org/10.1057/s41599-025-05618-w<br>• *Mara, M., Appel, M., & Gnambs, T. (2022). Human-like robots and the uncanny valley: A meta-analysis of user responses based on the Godspeed scales. Zeitschrift Für Psychologie, 230(1), 33–46.* https://doi.org/10.31234/osf.io/d4qc3<br>• *Oliveira, R., Arriaga, P., Santos, F. P., Mascarenhas, S., & Paiva, A. (2021). Towards prosocial design: A scoping review of the use of robots and virtual agents to trigger prosocial behaviour. Computers in Human Behavior, 114, 106547.* https://doi.org/10.1016/j.chb.2020.106547<br>• *Roesler, E., Manzey, D., & Onnasch, L. (2021). A meta-analysis on the effectiveness of anthropomorphism in human-robot interaction. Science Robotics, 6(58), 5425.* https://doi.org/10.1126/scirobotics.abj5425	of responses than persuasion in the conventional sense. For example, some included studies (listed in the supplementary material) assessed evaluative or experiential responses (e.g., trustworthiness, credibility, pleasantness, affinity, and communication competence). In addition, many included studies used paradigms in which participants evaluated AI- or human-generated outputs without engaging in interaction with a communicative partner.<br><br>In contrast, our review focused specifically on human responses in interaction contexts. We differentiated between distinct response types and examined their convergence or divergence between human-agent and human-human interactions. Therefore, our work provided a more fine-grained, cross-disciplinary synthesis of interaction-based responses. Nevertheless, we have removed the wording “first meta-analysis” to ensure a more cautious phrasing.<br>We fully agree that incorporating and discussing Huang and Wang (2023) and the other suggested work can strengthen the theoretical and empirical positioning of our review. We have now added related reviews and meta-analyses into the Introduction and Discussion sections.

While I appreciate that key concepts have been defined (e.g., agent, interaction), the theoretical derivation of the research questions, categories, and moderators would benefit from a more thorough literature review. I understand that, given the numerous variables and space limitations, the authors may have chosen not to include this. However, I believe it is still feasible to do so in a concise manner.	As suggested, we have added a paragraph in the section "The current review" to clarify the rationale for the research questions (Lines 158-167). In addition, we have clarified the selection of moderators based on prior literature (Lines 191-206).<br><br>Regarding outcome variables, response types and themes were classified a posteriori using an empirical-to-conceptual approach, rather than being predefined based on previous literature. This approach allowed the classification scheme to emerge directly from the extracted data and ensured that it reflected the range and diversity of human responses examined across studies. The classification process is described in the Methods and has now been clarified to explain and justify this approach (Lines 270-274).
The conceptual framework of the study, including the independent variable, the six categories as well as the moderator categories, could be visualized in a figure for clarity. Including example variables for each category and the moderators would help readers grasp the structure quickly.	We appreciate this suggestion and agree that a conceptual overview can improve clarity and readability. Accordingly, we have added a new figure (Fig. 1; p. 39) presenting the conceptual framework of the review. It will be placed at the end of the Introduction section (Line 207). This figure illustrates the independent variable (partner type), dependent variables (psychological and behavioural responses), and potential moderators. Because the response types and themes were classified post hoc rather than defined a priori, specific examples were not included in the framework figure.
Add the abbreviation MAIN to Sundar's Modality-Agency-Interactivity-Navigability model.	We have revised the wording to "the Modality-Agency-Interactivity-Navigability (MAIN) model" (Line 98).
RQ1 reads: "Which psychological and/or behavioral responses have been investigated in HAI compared to HHI?". In the results section, the "compared to HHI" part does not appear to be sufficiently addressed. If the intention was to explore which responses have been investigated in the field (rather than comparing them), consider rephrasing RQ1 for clarity.	We have revised RQ1 (Lines 149-150) to clarify that it identifies which psychological and behavioural responses have been investigated in studies that directly compare human-agent and human-human interactions. This revision makes explicit that RQ1 maps the scope of comparative evidence rather than implying an outcome comparison.

The dependent variables are currently defined in Table 1 and at the beginning of each respective results paragraph. How the moderators were defined remains unclear at times (e.g., partner appearance difference, p. 6). Although the authors have provided explanations on pp. 19f. and a detailed table on how they coded each variable in the Supplement, this information should be included in the main document (or at least referred to in the respective section on p. 6). A dedicated section that derives outcome variables and moderators from previous literature (e.g., Why was moderator XY included in the review?), with definitions and operationalizations, within a theoretical or conceptual framework (ending with the conceptual overview figure proposed above) would be ideal.	We thank the reviewer for this thoughtful suggestion. To improve clarity and transparency, we have added a new table (Table 1; p. 30-31) presenting all moderator variables along with definitions and coding scheme. We have also added a figure (Fig. 1; p. 39) presenting the conceptual framework of this review.<br><br>In addition, we have revised the end of the Introduction to clarify the selection of moderators based on prior literature (Lines 191-206).<br><br>Regarding outcome variables, response types and themes were classified a posteriori using an empirical-to-conceptual approach, rather than being predefined based on previous literature. The classification process is described in the Methods and has now been clarified to explain and justify this approach (Lines 270-274).
Consider adding descriptive statistics about the included papers (e.g., ratio of journal articles vs. conference papers) and sample characteristics (e.g., country of origin, mean age, gender distribution) on p. 4. This information is interesting for readers as it provides an overview of the papers and samples that were finally included.	As suggested, we have added these descriptive statistics at the beginning of the Results section (Lines 437-457).
p. 4: second paragraph in the results section: “[…] psychological and behavioral responses […]”	We have corrected the typographical error here (Line 436).
Are the summary statistics on p. 4 weighted to take into account different sample sizes (mean age, share of female participants)?	Thank you for this question. In the original version, those statistics were unweighted. We agree that sample-size-weighted means are more appropriate for summarising participant characteristics and have therefore revised the reported mean age and proportion of female participants accordingly (Lines 450-451). We did not calculate weighted SDs, as within-study variance information (e.g., age SDs) was often not reported across studies.

p. 6: Consider adding the moderator levels of partner appearance difference and interaction task in brackets for clarity.	We have now added the specific levels of these two moderators in brackets (Lines 578-579, 586).
p. 7: What is the difference between face-to-face vs. computer-mediated (interaction medium)?	We have clarified the coding levels of interaction medium in Table 1 (p. 31). Face-to-face refers to physically co-present interaction with partners, whereas computer-mediated refers to interaction with partners via a computer interface without physical co-presence. When the agent partner was a physically present robot, even if its face was displayed on a screen, the interaction medium was coded as face-to-face. Virtual reality-mediated refers to interaction occurring within virtual reality environments.
Use minus signs (−) for negative numbers, not hyphens (-).	We have revised the negative numbers in Tables 3 and 5 to use minus signs instead of hyphens (p. 34-38).
p. 8, Fig. 1: In the field "Records excluded (n = 10,640)", consider adding "after screening titles and abstracts" for transparency.	We have revised the wording in this figure to "Records excluded after screening titles and abstracts (n = 10,640)" (p. 40).
The discussion begins with a rather long summary of the (many) results (p. 15). Interpretation in light of previous literature is largely missing. Please refer back to the prior reviews cited in the introduction and discuss how your findings align or diverge from them.	As suggested, we have now discussed our findings in relation to prior reviews and meta-analyses, highlighting areas of alignment and divergence (Lines 663-766).
The introduction states: "This abundance of existing theoretical frameworks underscores the need for a cross-disciplinary, systematic understanding of human responses in HAI vs. HHI, and identifying factors influencing their similarities or differences" (p. 2). Please revisit this point in the discussion and provide more detailed explanations of how your findings address this need (p. 17). You additionally suggest expanding existing frameworks (Media Equation, Threshold Model of Social Influence) (p. 17). How exactly? There are already developments such as MASA (Modality-Agency-Sociality-Anthropomorphism) that account for different social cues. Please position your contribution relative to these.	We appreciate this constructive feedback and have expanded the theoretical implications accordingly. We have clarified how our review provides a cross-disciplinary, systematic understanding of human responses in human-agent vs. human-human interactions. Also, we have discussed our main and moderator findings relative to existing theoretical frameworks, including the Media Equation, Threshold Model of Social Influence, and cue-based frameworks (Lines 839-861).

Is there prior research affirming or contradicting the statement "this suggests that the cognitive mechanisms underlying core collaborative processes like trust and social alignment may be morphology-agnostic" (p. 17)? Please cite and discuss.	We have revised this sentence to clarify that we refer to trust and social alignment themselves as collaborative mechanisms, rather than cognitive mechanisms underlying them. We have also added discussion of related findings from prior meta-analyses (Lines 864-872).
The argument regarding intrinsic value (p. 1, 17), and different contexts (p. 17) may benefit from stronger theoretical grounding. Which theoretical approaches could explain this?	We have revised this paragraph to strengthen the theoretical grounding of these arguments by situating them within the moral agency-patiency framework and integrating related reviews and meta-analyses (Lines 917-935).
How do you expect response differences between HAI and HHI to evolve with the rise of generative AI? Will they diminish or grow stronger? What does this mean for theory, research, and practice? Please provide a reasoned outlook.	As suggested, we have added a reasoned outlook on how similarities and differences between HAI and HHI may evolve with the rise of generative AI (Lines 875-891).
The interpretation of moderator effects appears somewhat generic. Please name and discuss the most important moderators with implications for theory, research, and practice.	We have clarified the theoretical implications of the moderator findings (Lines 854-872). Also, we have expanded the practical implications by outlining how these findings inform agent design (Lines 943-969).
According to Viechtbauer (2025), the Knapp-Hartung adjustment is not available for the rma.mv() functon(https://cran.r-project.org/web/packages/metafor/metafor.pdf, p. 177). Please clarify where you used it and where you did not.	Thank you for pointing this out. The mention of the Knapp-Hartung adjustment in the manuscript was an oversight from an earlier plan. As we later employed the three-level meta-analysis, this adjustment was no longer applied. We have corrected the wording (Lines 357-358).<br><br>To avoid such issues, we have thoroughly reviewed the Data Analysis to ensure that the description of the analyses fully aligns with our R code.
As publication bias is one major concern in meta-analyses, consider including one or two sentences on how you checked for publication bias and whether there were issues in the main results section.	We have now added a brief description of how publication bias was assessed and whether any issues were detected (Lines 636-646).
The authors have not preregistered their study, although specific protocols exist for systematic reviews and meta-analyses, such as PRISMA-P (https://www.prisma-statement.org/protocols). I do not know	We thank the reviewer for highlighting the importance of preregistration. The analysis plan was documented in the first author's PhD milestone assessment report, but a formal protocol or preregistration was not

why the authors did not preregister their study – I just want to say that fear of deviations should not be the reason. For instance, PRISMA-P entails a standard template for reporting changes and amendments. In the meantime, I appreciate the availability of data and analysis code on osf very much.	conducted at the study's outset. To ensure transparency and reproducibility, we made all data, study materials, and analysis code publicly available via the OSF repository. We fully agree that preregistration is a critical practice for reducing bias and intend to incorporate it into our future research workflows.
The PRISMA checklist was added as as part of the large Supplement, and as a separate Supplement, one is sufficient.	We have retained the PRISMA checklist within the Supplementary Information and deleted the separate version.
I feel that there is much more information worth reporting in this review, including all the collected data about the papers and samples, as well as the remaining moderator analyses. One elegant way to enhance distribution and reproducibility might be to publish a distinct paper on the data (data descriptor), but this is simply a suggestion.	We appreciate this constructive suggestion. We agree that a dedicated data descriptor paper would be a valuable avenue for disseminating the compiled datasets and enhancing their reuse. As part of future updates to the review that incorporate emerging work on human interaction with GenAI systems, we will certainly consider preparing a data descriptor integrating these datasets.
